# Supplementary material for: Coagulation factor IX analysis in bioreactor cell culture supernatant predicts quality of the purified product
Source: Commun Biol. 2021 Mar 23;4:390. doi: 10.1038/s42003-021-01903-x (PMC7988164; doi:10.1038/s42003-021-01903-x)
Supplement: Supplementary file 2 — Supplementary Information [file 42003_2021_1903_MOESM2_ESM.pdf]

Supplementary Information

Coagulation factor IX analysis in bioreactor cell culture supernatant predicts quality of the purified product

Lucia F. Zacchi[1¥], Dinora Roche Recinos[1,2¥], Cassandra L. Pegg[3], Toan K. Phung[3], Mark Napoli[2], Campbell Aitken[2], Vanessa Sandford[2], Stephen M. Mahler[1], Yih Yean Lee[2*], Benjamin L. Schulz[1,3*], Christopher B. Howard[1*]

[1]ARC Training Centre for Biopharmaceutical Innovation, Australian Institute for Bioengineering and Nanotechnology, The University of Queensland, St. Lucia, QLD 4072, Australia.

[2] CSL Limited; Parkville Victoria, 3052, Australia

[3] School of Chemistry and Molecular Biosciences, The University of Queensland, St Lucia, Queensland, 4072, Australia

[¥] These authors contributed equally.

[*]Corresponding authors: YihYean.Lee@csl.com.au, b.schulz@uq.edu.au, c.howard2@uq.edu.au

Supplementary Material

Supplementary Tables

S1. CHO host cell proteins co-purifying with rFIX in samples from bioreactors H1 and/or H2 identified by DDA.

Names	Entry	Protein names	Organism	Calcium Binding	Subcellular location
Common in H1 and H2	**P00740**	**Coagulation factor IX**	**Human**	**Yes**	**S**
	**G3H8V5**	**Carboxypeptidase**	**CHO**	**Yes**	**S**
	**G3HHV4**	**Thrombospondin-1**	**CHO**	**Yes**	**S**
	G3I8R9	*Endoplasmic reticulum chaperone BiP*	*CHO*		*IC(S)*
	G3H354	Heat shock protein HSP 90-alpha	CHO		IC
	G3INX0	*Histone H2B*	*CHO*		*IC*
	G3H6V7	Lipoprotein lipase	CHO	Yes	S
	G3GYP9	*Peroxiredoxin-1*	*CHO*	*Yes*	*IC*
	G3I664	Procollagen C-endopeptidase enhancer 1	CHO		S
	G3IBF4	Serine protease HTRA1	CHO		S
	G3HQL6	Thioredoxin reductase 1, cytoplasmic	CHO		IC
	G3HNJ3	*Clusterin*	*CHO*		*S*
	G3GYG0	Vitamin K-dependent protein S	CHO	Yes	S
Only in H1	G3HQY6	Lipase	CHO		IC
Only in H2	G3HG36	Glutamine synthetase	CHO		IC
	G3HLB3	*Glutamine synthetase*	*CHO*		*IC*
	G3I027	Heat shock protein 75 kDa, mitochondrial	CHO		M
	G3IF52	Nucleobindin-2	CHO	Yes	S
	G3I3H2	60S acidic ribosomal protein P2	CHO		IC
	G3IFL2	*Multifunctional protein ADE2*	*CHO*		*N/F*
	G3HUC4	Sushi, von Willebrand factor type A	CHO	Yes	S
	G3GRV0	Kelch domain-containing protein 4	CHO		N/F
	G3INW9	Histone H2A	CHO		IC
	G3HMD1	Glyceraldehyde-3-phosphate dehydrogenase	CHO		IC
	G3I3U5	Nidogen-1	CHO	Yes	S
	G3HMI3	Semaphorin-3C	CHO		S
	G3HSL4	Elongation factor 2	CHO		IC
	G3HQM6	Endoplasmin	CHO		S
	G3GY17	Cullin-associated NEDD8-dissociated protein 1	CHO		IC
	G3HB04	Protein disulfide-isomerase A6	CHO		IC(S)
	G3H609	Glutathione reductase, mitochondrial	CHO		M
	G3IDS2	F-actin-capping protein subunit alpha	CHO		IC
	G3IH63	Myosin-9	CHO		IC
	G3HBI1	Peroxidasin-like	CHO		S

G3ID82	Beta-actin-like protein 2	CHO		IC
G3H0C2	Proteasome subunit alpha type	CHO		IC
G3HG95	Lamin-A/C	CHO		IC
G3IBK2	Filamin-A	CHO		IC
G3I5H3	Elongation factor 1-delta	CHO		IC
G3HXV5	*Nucleolar phosphoprotein p130*	*CHO*		*IC*
G3H3Q1	Pyruvate kinase	CHO		IC
G3GWR8	Proteasome endopeptidase complex	CHO		IC
G3I486	Heterochromatin protein 1-binding protein 3	CHO		IC
G3HCW9	PRDX2	CHO		IC
G3IK13	*Eukaryotic translation initiation factor 3 subunit C eIF3c*	*CHO*		*IC*
G3GZ90	Calumenin	CHO	Yes	IC(S)
G3H1W4	Tubulointerstitial nephritis antigen-like	CHO		IC
G3HSF3	Proteasome subunit alpha type	CHO		IC
G3I9G7	Proteasome subunit alpha type	CHO		IC

S=secretory; IC=intracellular; IC(S)=intracellular and possibly secreted; M=mitochondrial; N/F not found. Data was obtained from Uniprot. When no information was available on GO or calcium binding for the CHO proteins, the table was completed using data from the human homologs.

Bold: proteins significantly more abundant in H1 rFIX sample compared to H2 rFIX sample (DIA-MS and MSstats, $P < 10^{-5}$).

Italics: proteins significantly more abundant in H2 rFIX sample compared to H1 rFIX sample (DIA-MS and MSstats, $P < 10^{-5}$).

Supplementary Figures

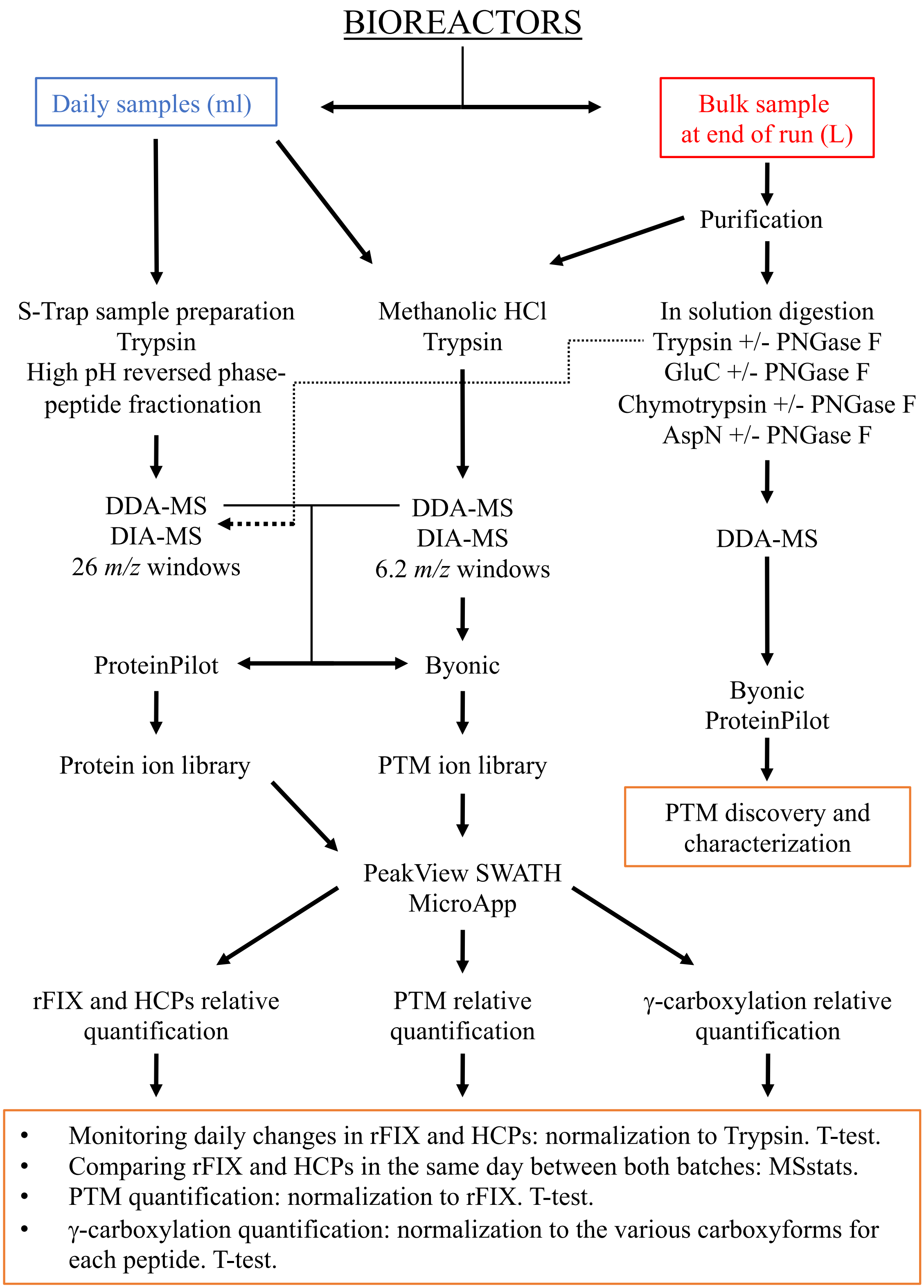

Supplementary Figure S1. Overview of the proteomic workflows used in this study.

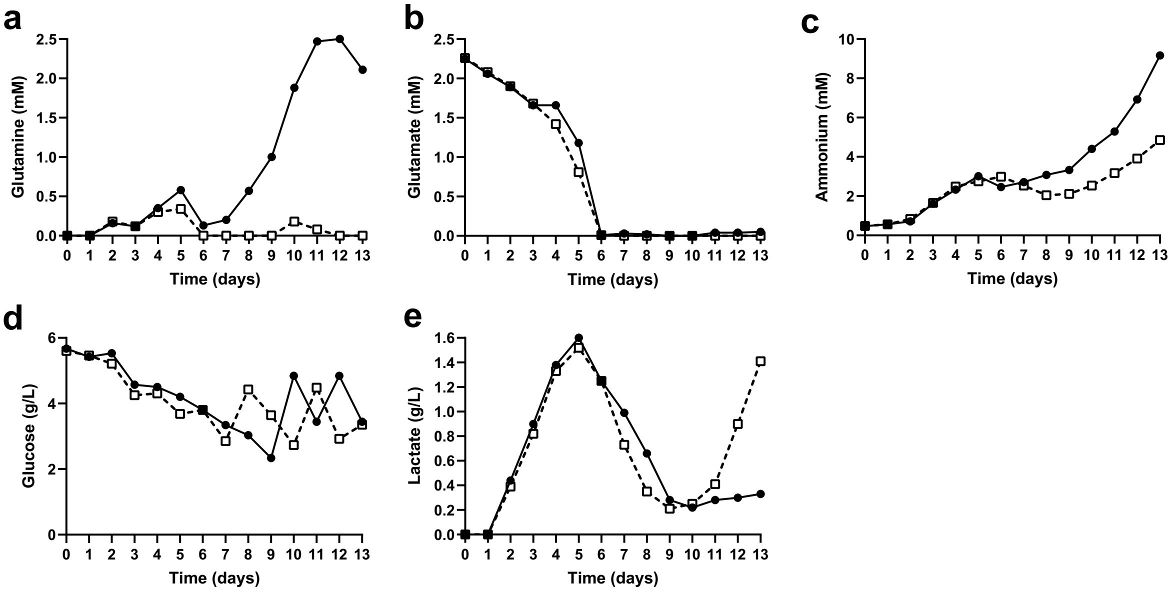

Supplementary Figure S2. Metabolic profile of CHO cells expressing rFIX in both fed batch conditions. CHO cells expressing rFIX and PACE/Furin were grown in fed batch bioreactor mode with either EfficientyFeed A (H1, solid line and black circles) or EfficientFeed B (H2, dotted line and open square). The following metabolites were measured: **a** glutamine, **b** glutamate, **c** ammonium, **d** glucose, and **e** lactate.

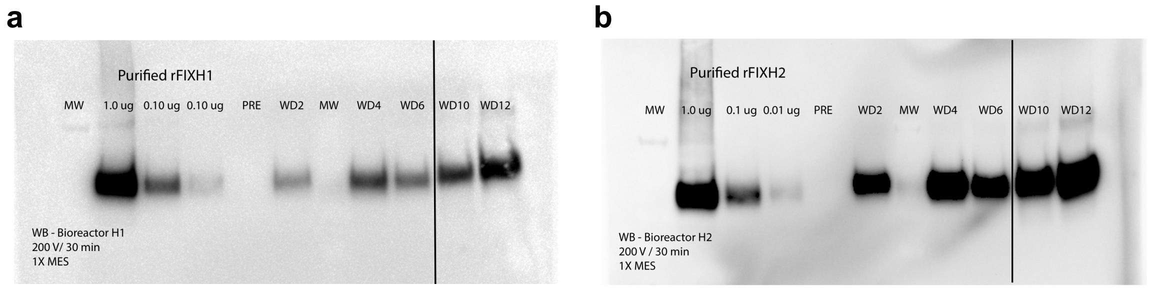

Supplementary Figure S3. Western blot of rFIX during bioreactor operation. a Bioreactor H1 and **b** bioreactor H2. Equal volumes of supernatant from H1 and H2 bioreactors at days 2, 4, 6, 10, and 12 were loaded onto the gel. Ten-fold dilutions of purified rFIX (1 mg/ml) from each bioreactor was used as a standard. PRE, medium before inoculation. rFIX showed the expected MW of ~ 57 kDa.

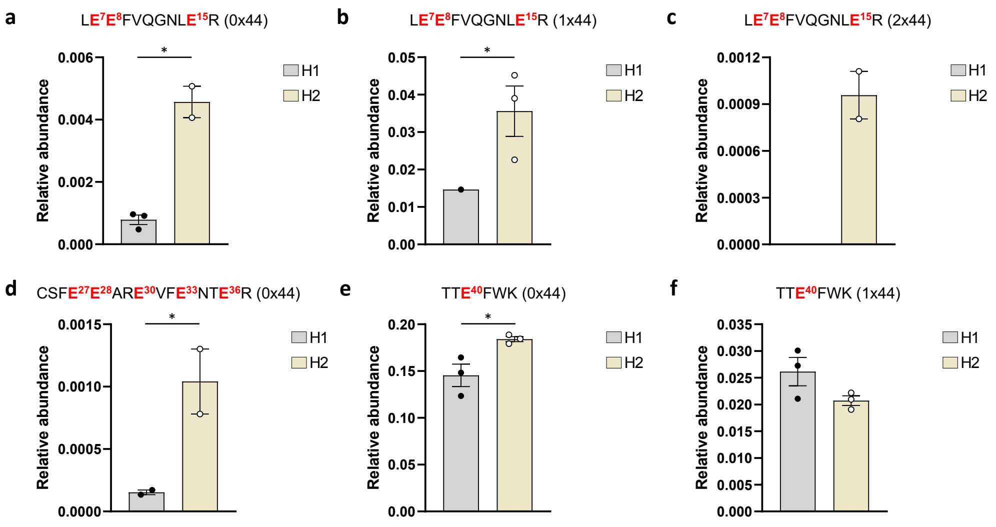

Supplementary Figure S4. Relative abundance of γ-carboxylated peptides from rFIX after purification. The abundance of select rFIX γ-carboxylated peptides (underivatized) was measured by DIA-MS in H1 and H2 bioreactors after rFIX purification. Graphs depict the mean +/- SEM of the abundance of each carboxypeptide relative to rFIX in purified samples from bioreactor H1 (black) or H2 (white). One-tailed t-est: * $p < 0.05$. N = 2 - 3 independent technical replicates. Shown are: **a-c** different carboxyforms of LE^{7}E^{8}FVQGNLE^{15}R, **a** uncarboxylated (0x44) (P = 0.0015), **b** mono γ-carboxylated (1x44) (P = 9.9×10^{-5}), or **c** di γ-carboxylated (2x44) at E$^{7/8/15}$; **d** uncarboxylated CSFE^{27}E^{28}ARE^{30}VFE^{33}NTE^{36}R (0x44) (P = 0.0382), **e-f** different carboxyforms of TTE^{40}FWK, **e** uncarboxylated (0x44) (P =0.0172) or **f** mono γ-carboxylated (1x44) at E^{40} (P = 0.0632). Individual data points are indicated in black (H1 bioreactor) or white circles (H2 bioreactor).

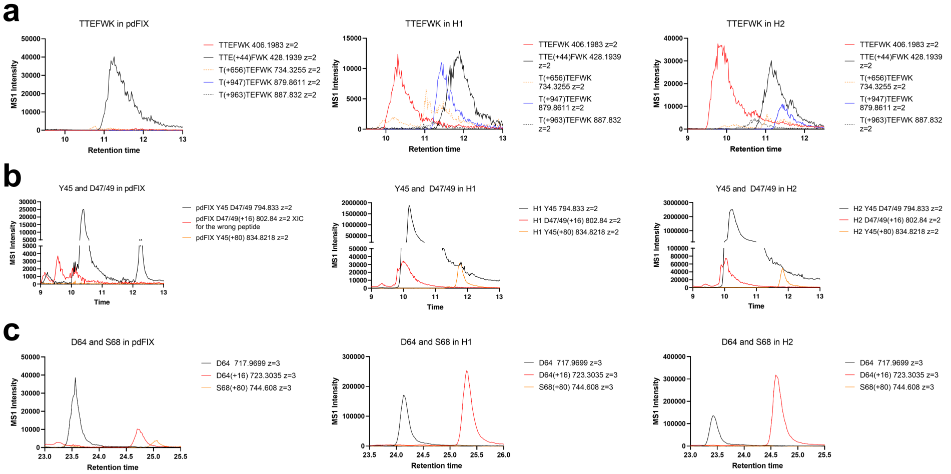

d

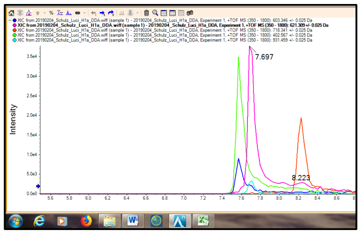

	m/z	RT	Glycoform	z
(green)	402.5665	7.7461	VSVSQTSKLTR	3
(blue)	603.3461	7.7811	VSVSQTSKLTR	2
(pink)	621.309	7.7191	VSVSQTS[+656.22761]KLTR	3
(cyan)	931.459	7.7191	VSVSQTS[+656.22761]KLTR	2
(red)	718.3408	8.2172	VSVSQTS[+947.32303]KLTR	3

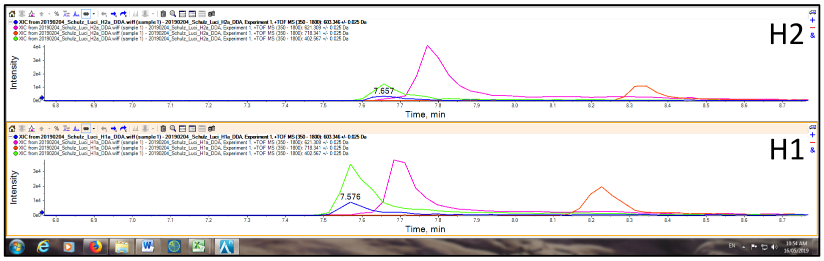

e

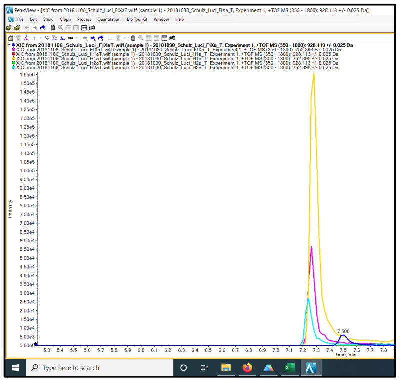

	m/z	Glycoform	z	FIX
(yellow)	752.898	IIPHHNYNAAINK	2	H1
(red)	928.113	IIPHHN(+2204)YNAAINK	4	H1
(cyan)	752.898	IIPHHNYNAAINK	2	H2
(green)	928.113	IIPHHN(+2204)YNAAINK	4	H2
(pink)	752.898	IIPHHNYNAAINK	2	pdFIX
(blue)	928.113	IIPHHN(+2204)YNAAINK	4	pdFIX

Supplementary Figure S5. MS1 precursor intensity for select peptides and glycopeptides of interest. a T^{38}TE^{40}FWK. **b** FWKQY^{45}VD^{47}GDQCE. **c** D^{64}DINS^{68}YECWCPFGFEGK. **d** VSVSQTS^{141}KLTR. **e** IIPHHN^{258}YNAAINK.

	% coverage			
	**Trypsin +/- PNGase F**	**Glu-C +/- PNGase F**	**Chymotrypsin +/- PNGase F**	**AspN +/- PNGase F**
**H1 rFIX**	83.95	84.6	77.01	58.53
**H2 rFIX**	76.14	81.78	77.87	43.17
**Plasma derived FIX**	72.45	69.85	65.94	N/A

rFIX H1 digested with trypsin or trypsin + PNGase F

MGRVNMIMAESPGLITICLLGYLLSAECTVFLDHENANKILNRVKRYNSGKLEEFVQGNLERECMEEKCSFEEAREV
FENTERTTEFWKQYVDGDQCESNPCLNGGSCKDDINSYECWCPFGFEGKNCELDVTCNIKNGRCEQFCKNSADNKVV
CSCTEGYRLAENQKSCEPAVPFPCGRVSVSQTSKLTRAETVFPDVDYVNSTEAETILDNITQSTQSFNDFTRVVGGE
DAKPGQFPWQVVLNGKVDAFCGGSIVNEKWIVTAAHCVETGVKITVVAGEHNIEETEHTEQKRNVIRIIPHHNYNAA
INKYNHDIALLELDEPLVLNSYVTPICIADKEYTNIFLKFGSGYVSGWGRVFHKGRSALVLQYLRVPLVDRATCLRS
TKFTIYNNMFCAGFHEGGRDSCQGDSGGPHVTEVEGTSFLTGIISWGEECAMKGKYGIYTKVSRYVNWIKEKTKLT

rFIX H2 digested with trypsin or trypsin + PNGase F

MGRVNMIMAESPGLITICLLGYLLSAECTVFLDHENANKILNRVKRYNSGKLEEFVQGNLERECMEEKCSFEEAREV
FENTERTTEFWKQYVDGDQCESNPCLNGGSCKDDINSYECWCPFGFEGKNCELDVTCNIKNGRCEQFCKNSADNKVV
CSCTEGYRLAENQKSCEPAVPFPCGRVSVSQTSKLTRAETVFPDVDYVNSTEAETILDNITQSTQSFNDFTRVVGGE
DAKPGQFPWQVVLNGKVDAFCGGSIVNEKWIVTAAHCVETGVKITVVAGEHNIEETEHTEQKRNVIRIIPHHNYNAA
INKYNHDIALLELDEPLVLNSYVTPICIADKEYTNIFLKFGSGYVSGWGRVFHKGRSALVLQYLRVPLVDRATCLRS
TKFTIYNNMFCAGFHEGGRDSCQGDSGGPHVTEVEGTSFLTGIISWGEECAMKGKYGIYTKVSRYVNWIKEKTKLT

Plasma derived FIX digested with trypsin or trypsin + PNGase F

MQRVNMIMAESPGLITICLLGYLLSAECTVFLDHENANKILNRPKRYNSGKLEEFVQGNLERECMEEKCSFEEAREV
FENTERTTEFWKQYVDGDQCESNPCLNGGSCKDDINSYECWCPFGFEGKNCELDVTCNIKNGRCEQFCKNSADNKVV
CSCTEGYRLAENQKSCEPAVPFPCGRVSVSQTSKLTRAETVFPDVDYVNSTEAETILDNITQSTQSFNDFTRVVGGE
DAKPGQFPWQVVLNGKVDAFCGGSIVNEKWIVTAAHCVETGVKITVVAGEHNIEETEHTEQKRNVIRIIPHHNYNAA
INKYNHDIALLELDEPLVLNSYVTPICIADKEYTNIFLKFGSGYVSGWGRVFHKGRSALVLQYLRVPLVDRATCLRS
TKFTIYNNMFCAGFHEGGRDSCQGDSGGPHVTEVEGTSFLTGIISWGEECAMKGKYGIYTKVSRYVNWIKEKTKLT

rFIX H1 digested with Glu-C or Glu-C + PNGase F

MGRVNMIMAESPGLITICLLGYLLSAECTVFLDHENANKILNRVKRYNSGKLEEFVQGNLERECMEEKCSFEEAREV
FENTERTTEFWKQYVDGDQCESNPCLNGGSCKDDINSYECWCPFGFEGKNCELDVTCNIKNGRCEQFCKNSADNKVV
CSCTEGYRLAENQKSCEPAVPFPCGRVSVSQTSKLTRAETVFPDVDYVNSTEAETILDNITQSTQSFNDFTRVVGGE
DAKPGQFPWQVVLNGKVDAFCGGSIVNEKWIVTAAHCVETGVKITVVAGEHNIEETEHTEQKRNVIRIIPHHNYNAA
INKYNHDIALLELDEPLVLNSYVTPICIADKEYTNIFLKFGSGYVSGWGRVFHKGRSALVLQYLRVPLVDRATCLRS
TKFTIYNNMFCAGFHEGGRDSCQGDSGGPHVTEVEGTSFLTGIISWGEECAMKGKYGIYTKVSRYVNWIKEKTKLT

rFIX H2 digested with Glu-C or Glu-C + PNGase F

MGRVNMIMAESPGLITICLLGYLLSAECTVFLDHENANKILNRVKRYNSGKLEEFVQGNLERECMEEKCSFEEAREV
FENTERTTEFWKQYVDGDQCESNPCLNGGSCKDDINSYECWCPFGFEGKNCELDVTCNIKNGRCEQFCKNSADNKVV
CSCTEGYRLAENQKSCEPAVPFPCGRVSVSQTSKLTRAETVFPDVDYVNSTEAETILDNITQSTQSFNDFTRVVGGE
DAKPGQFPWQVVLNGKVDAFCGGSIVNEKWIVTAAHCVETGVKITVVAGEHNIEETEHTEQKRNVIRIIPHHNYNAA
INKYNHDIALLELDEPLVLNSYVTPICIADKEYTNIFLKFGSGYVSGWGRVFHKGRSALVLQYLRVPLVDRATCLRS
TKFTIYNNMFCAGFHEGGRDSCQGDSGGPHVTEVEGTSFLTGIISWGEECAMKGKYGIYTKVSRYVNWIKEKTKLT

Plasma derived FIX digested with Glu-C or Glu-C + PNGase F

MQRVNMIMAESPGLITICLLGYLLSAECTVFLDHENANKILNRPKRYNSGKLEEFVQGNLERECMEEKCSFEEAREV
FENTERTTEFWKQYVDGDQCESNPCLNGGSCKDDINSYECWCPFGFEGKNCELDVTCNIKNGRCEQFCKNSADNKVV
CSCTEGYRLAENQKSCEPAVPFPCGRVSVSQTSKLTRAETVFPDVDYVNSTEAETILDNITQSTQSFNDFTRVVGGE
DAKPGQFPWQVVLNGKVDAFCGGSIVNEKWIVTAAHCVETGVKITVVAGEHNIEETEHTEQKRNVIRIIPHHNYNAA
INKYNHDIALLELDEPLVLNSYVTPICIADKEYTNIFLKFGSGYVSGWGRVFHKGRSALVLQYLRVPLVDRATCLRS
TKFTIYNNMFCAGFHEGGRDSCQGDSGGPHVTEVEGTSFLTGIISWGEECAMKGKYGIYTKVSRYVNWIKEKTKLT

rFIX H1 digested with Chymotrypsin or Chymotrypsin + PNGase F

MGRVNMIMAESPGLITICLLGYLLSAECTVFLDHENANKILNRVKRYNSGKLEEFVQGNLERECMEEKCSFEEAREV
FENTERTTEFWKQYVDGDQCESNPCLNGGSCKDDINSYECWCPFGFEGKNCELDVTCNIKNGRCEQFCKNSADNKVV
CSCTEGYRLAENQKSCEPAVPFPCGRVSVSQTSKLTRAETVFPDVDYVNSTEAETILDNITQSTQSFNDFTRVVGGE
DAKPGQFPWQVVLNGKVDAFCGGSIVNEKWIVTAAHCVETGVKITVVAGEHNIEETEHTEQKRNVIRIIPHHNYNAA
INKYNHDIALLELDEPLVLNSYVTPICIADKEYTNIFLKFGSGYVSGWGRVFHKGRSALVLQYLRVPLVDRATCLRS
TKFTIYNNMFCAGFHEGGRDSCQGDSGGPHVTEVEGTSFLTGIISWGEECAMKGKYGIYTKVSRYVNWIKEKTKLT

rFIX H2 digested with Chymotrypsin or Chymotrypsin + PNGase F

MGRVNMIMAESPGLITICLLGYLLSAECTVFLDHENANKILNRVKRYNSGKLEEFVQGNLERECMEEKCSFEEAREV
FENTERTTEFWKQYVDGDQCESNPCLNGGSCKDDINSYECWCPFGFEGKNCELDVTCNIKNGRCEQFCKNSADNKVV
CSCTEGYRLAENQKSCEPAVPFPCGRVSVSQTSKLTRAETVFPDVDYVNSTEAETILDNITQSTQSFNDFTRVVGGE
DAKPGQFPWQVVLNGKVDAFCGGSIVNEKWIVTAAHCVETGVKITVVAGEHNIEETEHTEQKRNVIRIIPHHNYNAA
INKYNHDIALLELDEPLVLNSYVTPICIADKEYTNIFLKFGSGYVSGWGRVFHKGRSALVLQYLRVPLVDRATCLRS
TKFTIYNNMFCAGFHEGGRDSCQGDSGGPHVTEVEGTSFLTGIISWGEECAMKGKYGIYTKVSRYVNWIKEKTKLT

Plasma derived FIX digested with Chymotrypsin or Chymotrypsin + PNGase F

MQRVNMIMAESPGLITICLLGYLLSAECTVFLDHENANKILNRPKRYNSGKLEEFVQGNLERECMEEKCSFEEAREV
FENTERTTEFWKQYVDGDQCESNPCLNGGSCKDDINSYECWCPFGFEGKNCELDVTCNIKNGRCEQFCKNSADNKVV
CSCTEGYRLAENQKSCEPAVPFPCGRVSVSQTSKLTRAETVFPDVDYVNSTEAETILDNITQSTQSFNDFTRVVGGE
DAKPGQFPWQVVLNGKVDAFCGGSIVNEKWIVTAAHCVETGVKITVVAGEHNIEETEHTEQKRNVIRIIPHHNYNAA
INKYNHDIALLELDEPLVLNSYVTPICIADKEYTNIFLKFGSGYVSGWGRVFHKGRSALVLQYLRVPLVDRATCLRS
TKFTIYNNMFCAGFHEGGRDSCQGDSGGPHVTEVEGTSFLTGIISWGEECAMKGKYGIYTKVSRYVNWIKEKTKLT

rFIX from H1 digested with AspN or AspN + PNGase F

MGRVNMIMAESPGLITICLLGYLLSAECTVFLDHENANKILNRVKRYNSGKLEEFVQGNLERECMEEKCSFEEAREV
FENTERTTEFWKQYVDGDQCESNPCLNGGSCKDDINSYECWCPFGFEGKNCELDVTCNIKNGRCEQFCKNSADNKVV
CSCTEGYRLAENQKSCEPAVPFPCGRVSVSQTSKLTRAETVFPDVDYVNSTEAETILDNITQSTQSFNDFTRVVGGE
DAKPGQFPWQVVLNGKVDAFCGGSIVNEKWIVTAAHCVETGVKITVVAGEHNIEETEHTEQKRNVIRIIPHHNYNAA
INKYNHDIALLELDEPLVLNSYVTPICIADKEYTNIFLKFGSGYVSGWGRVFHKGRSALVLQYLRVPLVDRATCLRS
TKFTIYNNMFCAGFHEGGRDSCQGDSGGPHVTEVEGTSFLTGIISWGEECAMKGKYGIYTKVSRYVNWIKEKTKLT

rFIX from H2 digested with AspN or AspN + PNGase F

MQRVNMIMAESPGLITICLLGYLLSAECTVFLDHENANKILNRPKRYNSGKLEEFVQGNLERECMEEKCSFEEAREV
FENTERTTEFWKQYVDGDQCESNPCLNGGSCKDDINSYECWCPFGFEGKNCELDVTCNIKNGRCEQFCKNSADNKVV
CSCTEGYRLAENQKSCEPAVPFPCGRVSVSQTSKLTRAETVFPDVDYVNSTEAETILDNITQSTQSFNDFTRVVGGE
DAKPGQFPWQVVLNGKVDAFCGGSIVNEKWIVTAAHCVETGVKITVVAGEHNIEETEHTEQKRNVIRIIPHHNYNAA
INKYNHDIALLELDEPLVLNSYVTPICIADKEYTNIFLKFGSGYVSGWGRVFHKGRSALVLQYLRVPLVDRATCLRS
TKFTIYNNMFCAGFHEGGRDSCQGDSGGPHVTEVEGTSFLTGIISWGEECAMKGKYGIYTKVSRYVNWIKEKTKLT

Supplementary Figure S6. FIX sequence coverage from DDA searches in ProteinPilot (SCIEX) of purified rFIX from bioreactors H1 and H2 or pdFIX digested with several single proteases +/- PNGase F. The table indicates the percentage of coverage. The sequences below show the coverage using the following color coding that indicates confidence assignment: Green: high; yellow: medium; red: low.

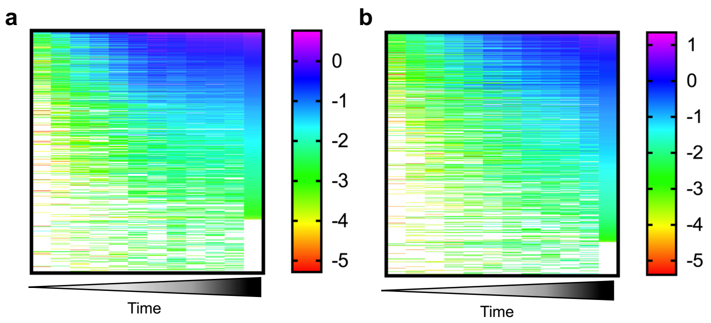

Supplementary Figure S7. Host cell proteins (HCP) changes in relative abundance during culture. Heatmaps depicting the change in normalized relative abundance of HCPs through time (Day 1 to 13) in bioreactors H1 **a** and H2 **b**. Secreted HCPs were quantified by DIA-MS, normalized to trypsin, and log10 transformed. Each line represents each quantified HCP (N = 2 – 3, independent technical replicates).

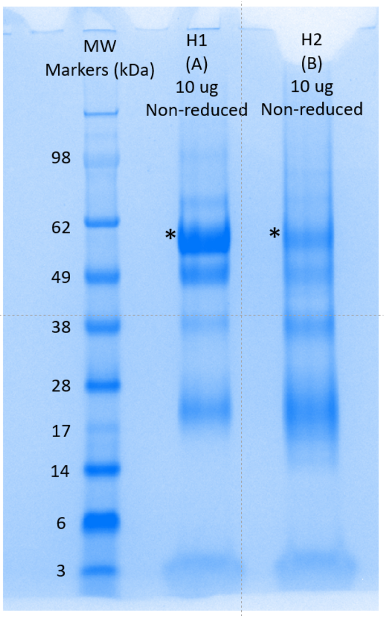

Supplementary Figure S8. Coomassie Blue stained non-reducing SDS-PAGE of purified rFIX from H1 and H2 fed-batch bioreactors. 10 µg total purified protein from H1 and H2 bioreactors were loaded in the gel. The asterisks show rFIX (estimated MW ~ 57 kDa).
