# Supplementary material for: Coagulation factor IX analysis in bioreactor cell culture supernatant predicts quality of the purified product
Source: Commun Biol. 2021 Mar 23;4:390. doi: 10.1038/s42003-021-01903-x (PMC7988164; doi:10.1038/s42003-021-01903-x)
Supplement: Supplementary file 3 — Description of Additional Supplementary Files [file 42003_2021_1903_MOESM3_ESM.pdf]

Description of Additional Supplementary Files

File name: Supplementary Data S1
Description: Viability and metabolic data for CHO cells expressing rFIX from bioreactors H1 and H2.

File name: Supplementary Data S2
Description: Comparison of the relative abundance of proteins before (supernatant) and after purification.

File name: Supplementary Data S3
Description: PeakView output of the quantification of FIX PTMs abundance during bioreactor operation and in the purified material. FDR measurements, fragment ion intensity measurements, peptide intensity measurements, protein intensity measurement, and peptide intensity measurement filtered based on FDR.

File name: Supplementary Data S4
Description: PeakView output of the quantification of methylated rFIX GLA domain. This file contains several tabs: A) Byonic searches for methyl-carboxy rFIX characterization, focused on the GLA domain. B) Ion library used to measure methyl-carboxy rFIX GLA domain during bioreactor operation and in the purified material. C-F) PeakView output of the quantification of methylcarboxy rFIX abundance during bioreactor operation and in the purified material, using either 1. XIC 6 min or 2. XIC 2 min, C) FDR measurements, D) Fragment ion intensity measurements, E) Peptide intensity measurements, F) Peptide intensity measurement filtered based on FDR. G) Statistical comparisons.

File name: Supplementary Data S5
Description: Output of the Byonic searches for rFIX characterization of PTMs using Trypsin, GluC, AspN, or Chymotrypsin, and +/- PNGase F.

File name: Supplementary Data S6
Description: Annotated MS/MS spectra for H1 rFIX. Annotated MS/MS spectra from Byonic of all the post-translationally modified peptides used for the characterization of H1 rFIX digested with multiple single proteases and +/- PNGase F.

File name: Supplementary Data S7
Description: Annotated MS/MS spectra for H2 rFIX. Annotated MS/MS spectra from Byonic of all the post-translationally modified peptides used for the characterization of H2 rFIX digested with multiple single proteases and +/- PNGase F.

File name: Supplementary Data S8
Description: Annotated MS/MS spectra for pdFIX. Annotated MS/MS spectra from Byonic of all the posttranslationally modified peptides used for the characterization of pdFIX digested with multiple single proteases and +/- PNGase F.

File name: Supplementary Data S9
Description: Annotated MS/MS spectra for PTM quantification. Annotated MS/MS spectra from Byonic of all the post-translationally modified peptides used for the quantification of PTMs in rFIX by DIA-MS.

File name: Supplementary Data S10
Description: Annotated MS/MS spectra for GLA methyl quantification. Annotated MS/MS spectra from Byonic of all the post-translationally modified peptides used for the quantification of methylated GLA peptides in rFIX by DIA-MS.

File name: Supplementary Data S11
Description: ProteinPilot output of search for peptides and proteins in a purified sample from bioreactor H1.

File name: Supplementary Data S12
Description: Ion library used to measure select FIX PTMs abundance during bioreactor operation and in the purified material.

File name: Supplementary Data S13
Description: ProteinPilot output of combined searches for peptides and proteins in the fractionated and unfractionated supernatant and purified samples from bioreactors H1 and H2.

File name: Supplementary Data S14
Description: Ion library used to measure the abundance of rFIX and host-cell proteins during bioreactor operation and in the purified material.

File name: Supplementary Data S15
Description: PeakView output of the quantification of rFIX and host-cell proteins abundance during bioreactor operation and in the purified material. FDR measurements, fragment ion intensity measurements, peptide intensity measurements, protein intensity measurements, protein intensity measurement recalculated based on FDR, and FDR-filtered protein intensity measurement normalized to trypsin.

File name: Supplementary Data S16
Description: GO and network analyses of the proteins that were differentially abundant in the supernatant of bioreactor H1 vs H2 at day 13.

File name: Supplementary Data S17
Description:. Glycan databases for searching in Byonic: N-glycan database 50 and 57 common biantennary glycans from Byonic combined with 14 additional N-glycoforms with LacNAc extensions, and Oglycan database with 11 O-glycans.

File name: Supplementary Data S18
Description: Output of the Byonic searches of DDA files of pdFIX and H1 and H2 rFIX digested with

trypsin and selected PSMs used for rFIX PTM quantification by DIA-MS.

File name: Supplementary Data S19
Description: ProteinPilot output of search for peptides and proteins in a purified sample from bioreactor H2.

File name: Supplementary Data S20
Description: Python script. Python script to recalculate peptide and protein abundance with peptide level 1% FDR cut-off.

File name: Supplementary Data S21
Description:. Output of the Byonic searches of DDA files of methylated H1 and H2 rFIX digested with trypsin, and selected PSMs used for rFIX GLA g-carboxylation quantification by DIA-MS.

File name: Supplementary Data S22
Description: Description of DDA and DIA-MS files uploaded in ProteomeXchange with the dataset identifier PXD018229.

File name: Supplementary Data S23
Description: Multiple t-test result. Results of the statistical analysis of rFIX/trypsin values during bioreactor operation. Multiple t-tests, with the two-stage linear set-up procedure of Benjamini, Krieger, and Yekutieli and Q = 1%, in GraphPad Prism.

File name: Supplementary Data S24
Description: Original quantitative data – Prism files.
