# Supplementary material for: Coagulation factor IX analysis in bioreactor cell culture supernatant predicts quality of the purified product
Source: Commun Biol. 2021 Mar 23;4:390. doi: 10.1038/s42003-021-01903-x (PMC7988164; doi:10.1038/s42003-021-01903-x)
Supplement: Supplementary file 9 — Supplementary Data S6 [file 42003_2021_1903_MOESM9_ESM.pdf]

20190701_Co4Ra2NeuGcOglyLacNAc_20190509_BenSchulz_Luci_H1a_Ch-P.wiff_Byonic_D359_D364 1
20190701_Co4Ra2NeuGcOglyLacNAc_20190509_BenSchulz_Luci_H1a_Ch-P.wiff_Byonic_D359[+16] 2
20190701_Co4Ra2NeuGcOglyLacNAc_20190509_BenSchulz_Luci_H1a_Ch-P.wiff_Byonic_D364[+16] 3
20190701_Co4Ra2NeuGcOglyLacNAc_20190509_BenSchulz_Luci_H1a_Ch.wiff_Byonic_E33_E36_E40 4
20190701_Co4Ra2NeuGcOglyLacNAc_20190509_BenSchulz_Luci_H1a_Ch.wiff_Byonic_E33_E36_E40[+44] 5

20190701_Co4Ra2NeuGcOglyLacNAc_20190509_BenSchulz_Luci_H1a_Ch.wiff_Byonic_E33_E36_E40[+44]x2 6
20190701_Co4Ra2NeuGcOglyLacNAc_20190509_BenSchulz_Luci_H1a_Ch.wiff_Byonic_T179 7
20190701_Co4Ra2NeuGcOglyLacNAc_20190509_BenSchulz_Luci_H1a_Ch.wiff_Byonic_T179[+947] 8
20190701_Co4Ra2NeuGcOglyLacNAc_20190509_BenSchulz_Luci_H1a_Ch.wiff_Byonic_T179[+963] 9
Common4rare2NeuGc50_20181106_Schulz_Luci_H1aG.wiff_20181116_Byonic(1)__S53[+426]_S61[+802]_D64[+16]_S68[+146] 10

Common4rare2NeuGc50_20181106_Schulz_Luci_H1aG.wiff_20181116_Byonic(1)_D49[+16] 11
Common4rare2NeuGc50_20181106_Schulz_Luci_H1aG.wiff_20181116_Byonic(1)_D292[+16] 12
Common4rare2NeuGc50_20181106_Schulz_Luci_H1aG.wiff_20181116_Byonic(1)_E7_E9_E15_[+44]x2 13
Common4rare2NeuGc50_20181106_Schulz_Luci_H1aG.wiff_20181116_Byonic(1)_N258 14
Common4rare2NeuGc50_20181106_Schulz_Luci_H1aG.wiff_20181116_Byonic(1)_N258[+1] 15

Common4rare2NeuGc50_20181106_Schulz_Luci_H1aG.wiff_20181116_Byonic(1)_S53[+426]_S61[+802]_D64[+16]_D65[+16]_S 16
Common4rare2NeuGc50_20181106_Schulz_Luci_H1aGP_wiff_20181116_Byonic_D292[+16] 17
Common4rare2NeuGc50_20181106_Schulz_Luci_H1aGP.wiff_20181116_Byonic_N157[+1] 18
Common4rare2NeuGc50_20181106_Schulz_Luci_H1aGP.wiff_20181116_Byonic_N167[+1} 19
Common4rare2NeuGc50_20181106_Schulz_Luci_H1aGP.wiff_20181116_Byonic_S141[+656] 20

Common4rare2NeuGc50_20181106_Schulz_Luci_H1aGP.wiff_20181116_Byonic_S158[+947] 21
Common4rare2NeuGc50_20181106_Schulz_Luci_H1aGP.wiff_20181116_Byonic_Y155[+80] 22
Common4rare2NeuGc50_20181106_Schulz_Luci_H1aTP.wiff_20181119_Byonic_D64 23
Common4rare2NeuGc50_20181106_Schulz_Luci_H1aTP.wiff_20181119_Byonic_D64[+16] 24
Common4rare2NeuGc50_20181106_Schulz_Luci_H1aTP.wiff_20181119_Byonic_D85[+16] 25

Common4rare2NeuGc50_20181106_Schulz_Luci_H1aTP.wiff_20181119_Byonic_D186 26
Common4rare2NeuGc50_20181106_Schulz_Luci_H1aTP.wiff_20181119_Byonic_D186[+16] 27
Common4rare2NeuGc50_20181106_Schulz_Luci_H1aTP.wiff_20181119_Byonic_D203 28
Common4rare2NeuGc50_20181106_Schulz_Luci_H1aTP.wiff_20181119_Byonic_D203[+16] 29
Common4rare2NeuGc50_20181106_Schulz_Luci_H1aTP.wiff_20181119_Byonic_E7_E9_E15 30

Common4rare2NeuGc50_20181106_Schulz_Luci_H1aTP.wiff_20181119_Byonic_E7_E9_E15{+44] 31
Common4rare2NeuGc50_20181106_Schulz_Luci_H1aTP.wiff_20181119_Byonic_E7_E9_E15{+44]x2 32
Common4rare2NeuGc50_20181106_Schulz_Luci_H1aTP.wiff_20181119_Byonic_E7_E9_E15{+44]x3 33
Common4rare2NeuGc50_20181106_Schulz_Luci_H1aTP.wiff_20181119_Byonic_S141[+947] 34
Common4rare2NeuGc50_20181106_Schulz_Luci_H1aTP.wiff_20181119_Byonic_T38[+656] 35

Common4rare2NeuGc50_20181106_Schulz_Luci_H1aTP.wiff_20181119_Byonic_T38[+947] 36
Common4rare2NeuGc50_20181106_Schulz_Luci_H1aTP.wiff_20181119_Byonic_T38[+963] 37
Common4rare2NeuGc50_20181106_Schulz_Luci_H1bG.wiff_20181116_Byonic D47[+16] 38
Common4rare2NeuGc50_20181106_Schulz_Luci_H1bG.wiff_20181116_Byonic_D85 39
Common4rare2NeuGc50_20181106_Schulz_Luci_H1bG.wiff_20181116_Byonic_S53[+426]_S61[+802]_D64[+16] 40

Common4rare2NeuGc50_20181106_Schulz_Luci_H1bG.wiff_20181116_Byonic_S53[+426]_S61[+802] 41
Common4rare2NeuGc50_20181106_Schulz_Luci_H1bG.wiff_20181116_Byonic_S53[+426]_S61[+818]_D64[+16] 42
Common4rare2NeuGc50_20181106_Schulz_Luci_H1bG.wiff_20181116_Byonic_S110[+426]_T112[+802] 43
Common4rare2NeuGc50_20181106_Schulz_Luci_H1bG.wiff_20181116_Byonic_s123_s141_potential 44
Common4rare2NeuGc50_20181106_Schulz_Luci_H1bG.wiff_20181116_Byonic_S141 45

Common4rare2NeuGc50_20181106_Schulz_Luci_H1bG.wiff_20181116_Byonic_Y45_D47_D49 ____ 46
Common4rare2NeuGc50_20181106_Schulz_Luci_H1bG.wiff_20181116_Byonic_Y45[+80} ____ 47
Common4rare2NeuGc50_20181106_Schulz_Luci_H1bG.wiff_20181116_Byonic_Y45[+80}sulfo_immonium ____ 48
Common4rare2NeuGc50_20181106_Schulz_Luci_H1bGP.wiff_20181116_Byonic_D104 ____ 49
Common4rare2NeuGc50_20181106_Schulz_Luci_H1bGP.wiff_20181116_Byonic_D104{+16] ____ 50

Common4rare2NeuGc50_20181106_Schulz_Luci_H1bGP.wiff_20181116_Byonic_D276-D292 51
Common4rare2NeuGc50_20181106_Schulz_Luci_H1bGP.wiff_20181116_Byonic_D276[+16] 52
Common4rare2NeuGc50_20181106_Schulz_Luci_H1bGP.wiff_20181116_Byonic_s365_672 T371 426 potential 53
Common4rare2NeuGc50_20181106_Schulz_Luci_H1bGP.wiff_20181116_Byonic_Y155[+80] 54
Common4rare2NeuGc50_20181106_Schulz_Luci_H1bT.wiff_20181119_Byonic_D203 55

Common4rare2NeuGc50_20181106_Schulz_Luci_H1bT.wiff_20181119_Byonic_D203[+16] 56
Common4rare2NeuGc50_20181106_Schulz_Luci_H1bT.wiff_20181119_Byonic_S141[+656] 57
ProteinPilot_E40 58
ProteinPilot_E40[+44] 59
ProteinPilot_S158[+656] 60

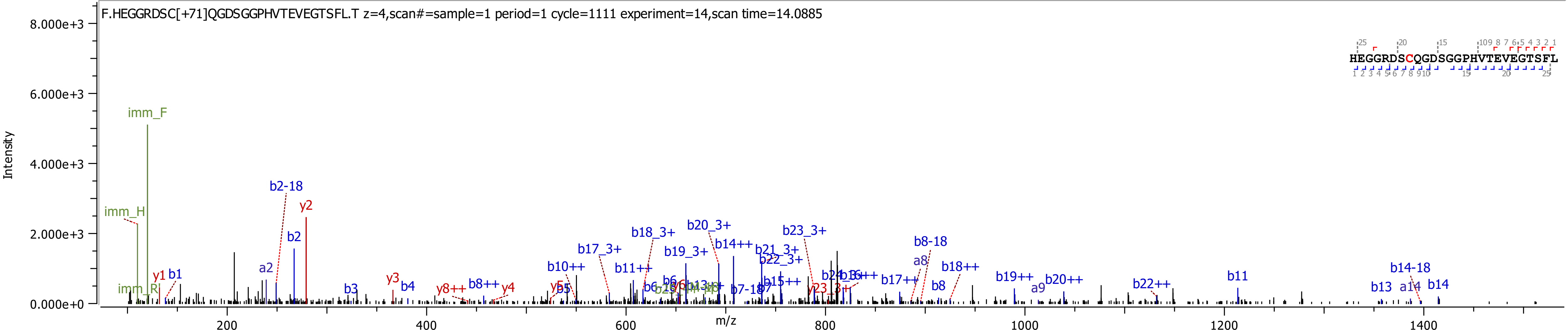

F.HEGGRDSC[+71]QGDSGGPHVTEVEGTSFL.T z=4,scan#=sample=1 period=1 cycle=1111 experiment=14,scan time=14.0885
HEGGRDSCQGDSGGPHVTEVEGTSFL
Intensity
m/z
imm_F
imm_H
imm_R
y1
b1
a2
b2-18
b2
y2
b3
y3
b4
y8++
b8++
y4
b10++
b17_3+
b11++
b18_3+
b19_3+
b20_3+
b14++
b21_3+
b22_3+
b23_3+
b8-18
a8
b18++
b17++
b8
b19++
a9
b20++
b22++
b11
b14-18
b13
a14
b14

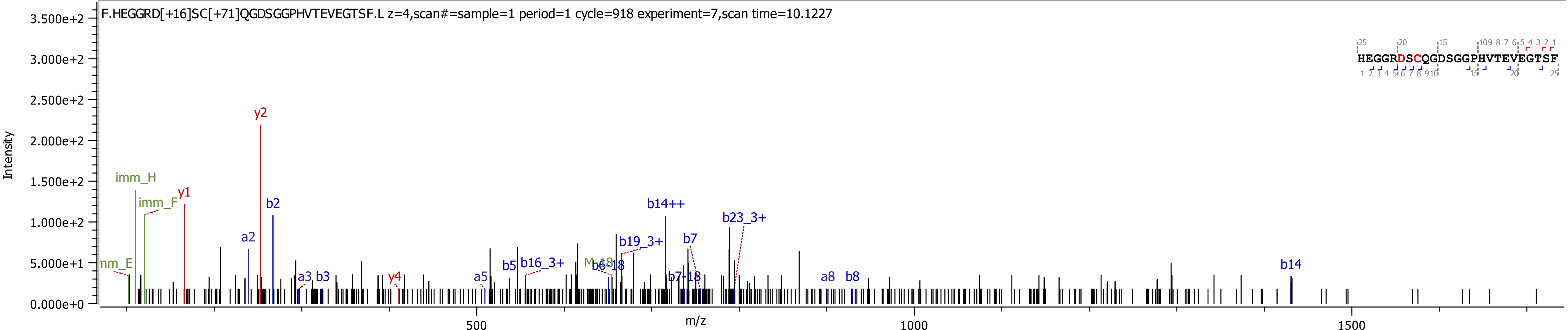
F.HEGGRD[+16]SC[+71]QGDSGGPHVTEVEGTSF.L z=4,scan#=sample=1 period=1 cycle=918 experiment=7,scan time=10.1227
HEGGRDSCQGDSGGPHVTEVEGTSF
Intensity
m/z
imm_H
imm_F
y1
y2
b2
a2
a3
b3
y4
a5
b5
b16_3+
b6-18
b19_3+
b14++
b7-18
b7
b23_3+
a8
b8
b14
0.000e+0
5.000e+1
1.000e+2
1.500e+2
2.000e+2
2.500e+2
3.000e+2
3.500e+2
500
1000
1500

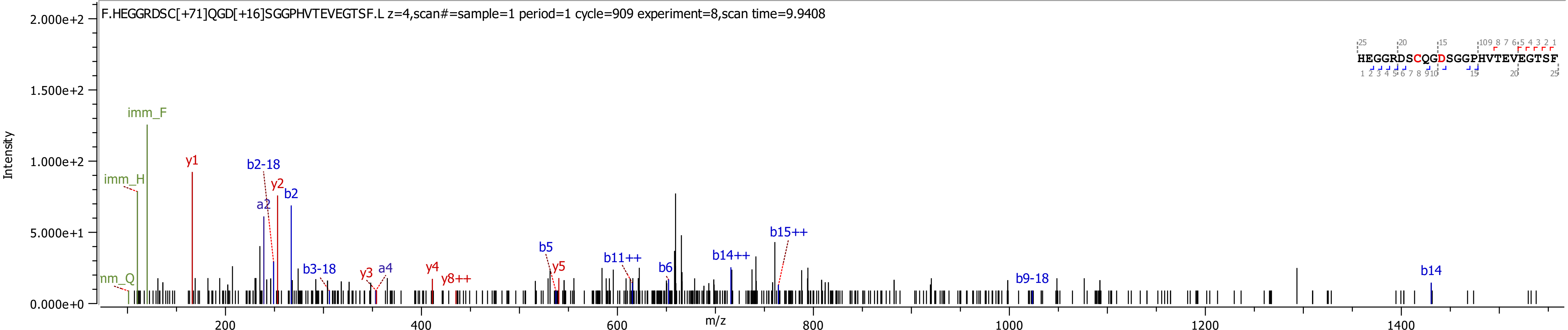
F.HEGGRDSC[+71]QGD[+16]SGGPHVTEVEGTSF.L z=4,scan#=sample=1 period=1 cycle=909 experiment=8,scan time=9.9408
HEGGRDSCQGDSGGPHVTEVEGTSF
Intensity
m/z
imm_F
imm_H
nm_Q
y1
b2-18
y2
b2
a2
b3-18
y3
a4
y4
y8++
b5
y5
b11++
b6
b14++
b15++
b9-18
b14
2.000e+2
1.500e+2
1.000e+2
5.000e+1
0.000e+0
200
400
600
800
1000
1200
1400

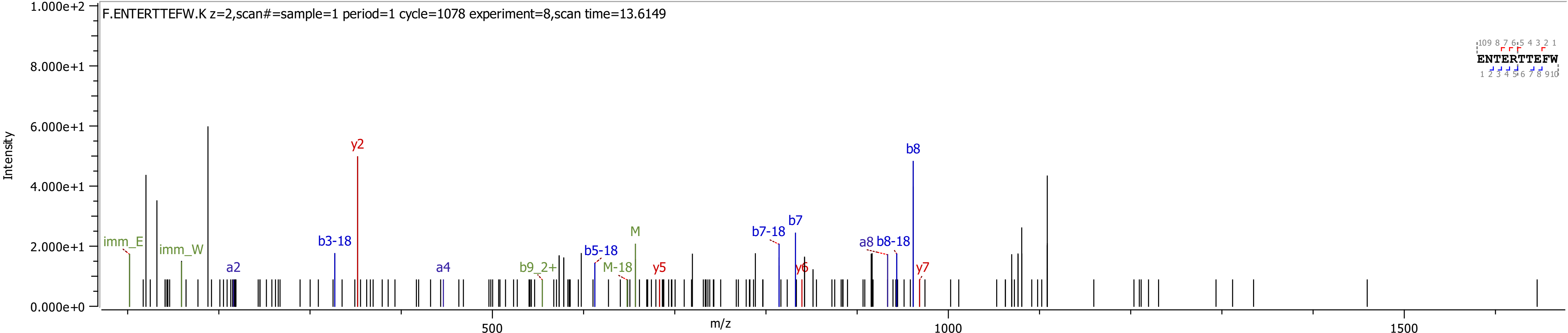
F.ENTERTTEFW.K z=2,scan#=sample=1 period=1 cycle=1078 experiment=8,scan time=13.6149
Intensity
m/z
0.000e+0
2.000e+1
4.000e+1
6.000e+1
8.000e+1
1.000e+2
500
1000
1500
imm_E
imm_W
a2
b3-18
y2
a4
b9_2+
b5-18
M-18
M
y5
b7-18
b7
y6
a8
b8-18
b8
y7
ENTERTTEFW

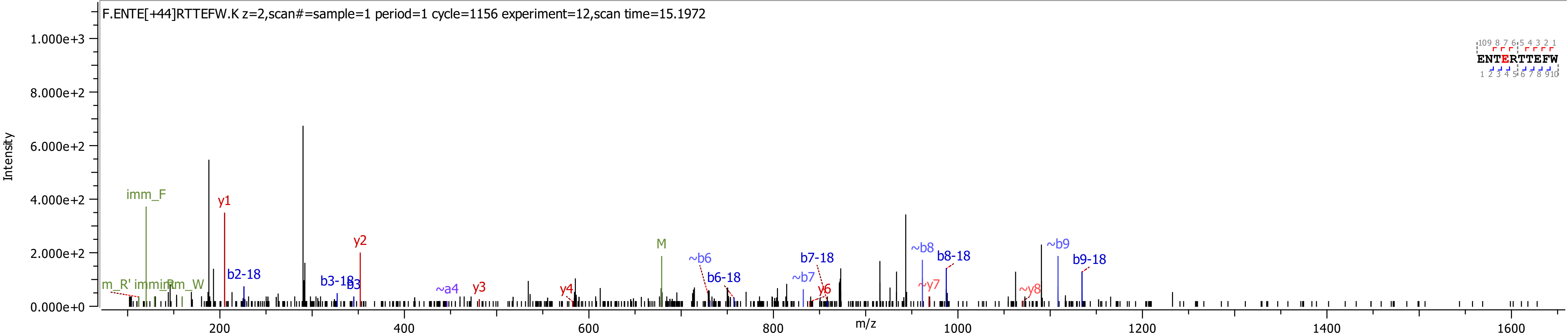
F.ENTE[+44]RTTEFW.K z=2,scan#=sample=1 period=1 cycle=1156 experiment=12,scan time=15.1972
Intensity
m/z
ENTERTTEFW
imm_F
y1
y2
y3
y4
y6
~y7
~y8
b2-18
b3-18
b3
~a4
M
~b6
b6-18
~b7
b7-18
~b8
b8-18
~b9
b9-18
m_R'
imm_R
m_W

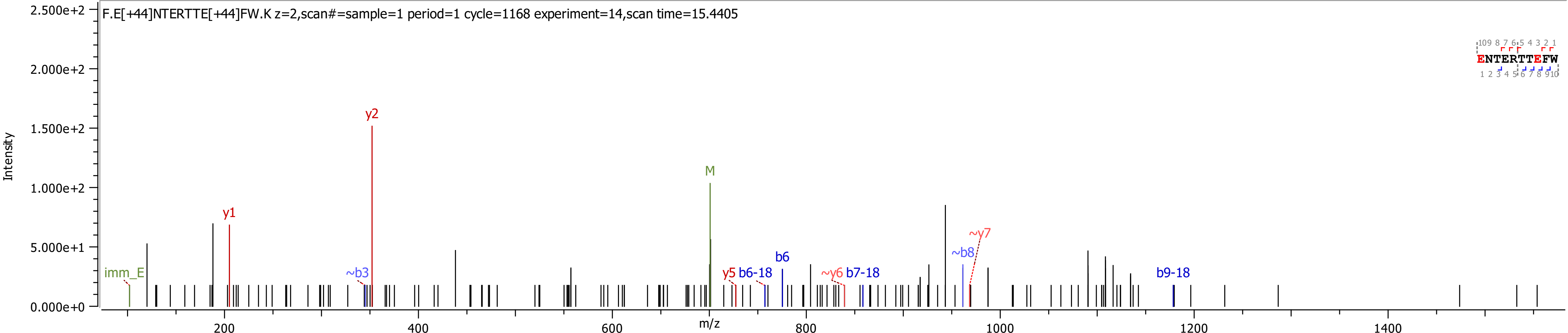
F.E[+44]NTERTTE[+44]FW.K z=2,scan#=sample=1 period=1 cycle=1168 experiment=14,scan time=15.4405
Intensity
2.500e+2
2.000e+2
1.500e+2
1.000e+2
5.000e+1
0.000e+0
m/z
200
400
600
800
1000
1200
1400
imm_E
y1
~b3
y2
M
y5
b6-18
b6
~y6
b7-18
~b8
~y7
b9-18
ENTERTTEFW

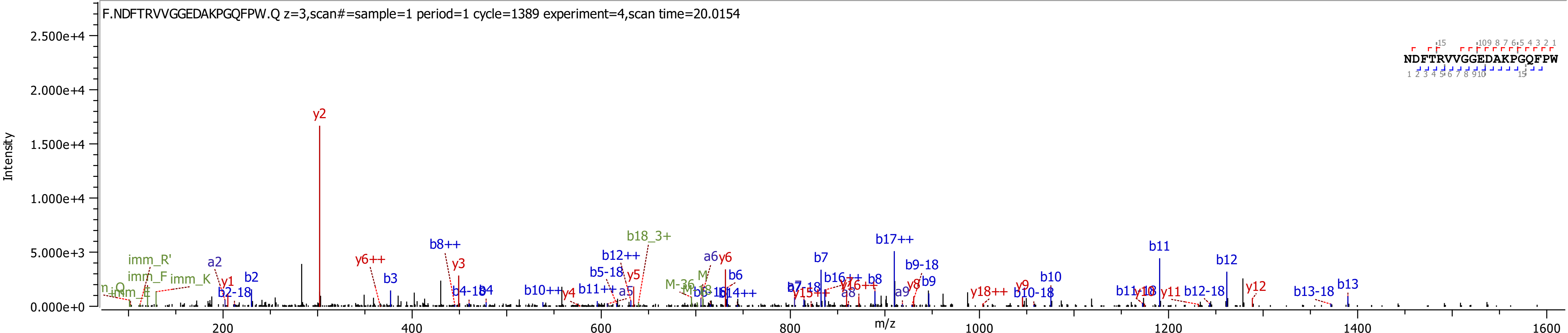
F.NDFTRVVGGEDAKPGQFPW.Q z=3,scan#=sample=1 period=1 cycle=1389 experiment=4,scan time=20.0154
Intensity
m/z
NDFTRVVGGEDAKPGQFPW

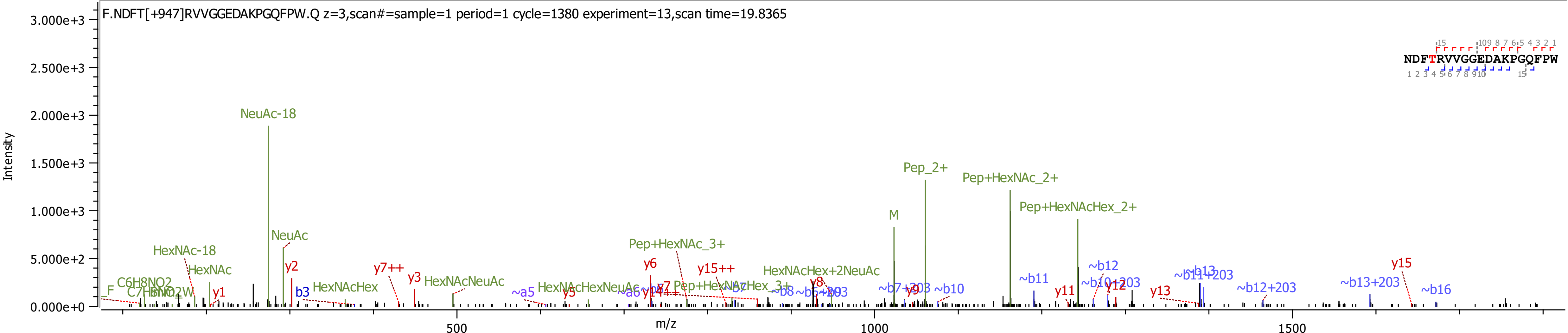
F.NDFT[+947]RVVGGEDAKPGQFPW.Q z=3,scan#=sample=1 period=1 cycle=1380 experiment=13,scan time=19.8365
Intensity
m/z
NDFTRVVGGEDAKPGQFPW
NeuAc-18
NeuAc
HexNAc-18
HexNAc
y2
y1
b3
HexNAcHex
y7++
y3
HexNAcNeuAc
~a5
y5
y6
y15++
Pep+HexNAc_3+
HexNAcHex+2NeuAc
y8
M
Pep_2+
Pep+HexNAc_2+
Pep+HexNAcHex_2+
~b7+203
~b10
~b11
~b12
y11
y12
y13
~b12+203
~b13+203
y15
~b16
3.000e+3
2.500e+3
2.000e+3
1.500e+3
1.000e+3
5.000e+2
0.000e+0
500
1000
1500

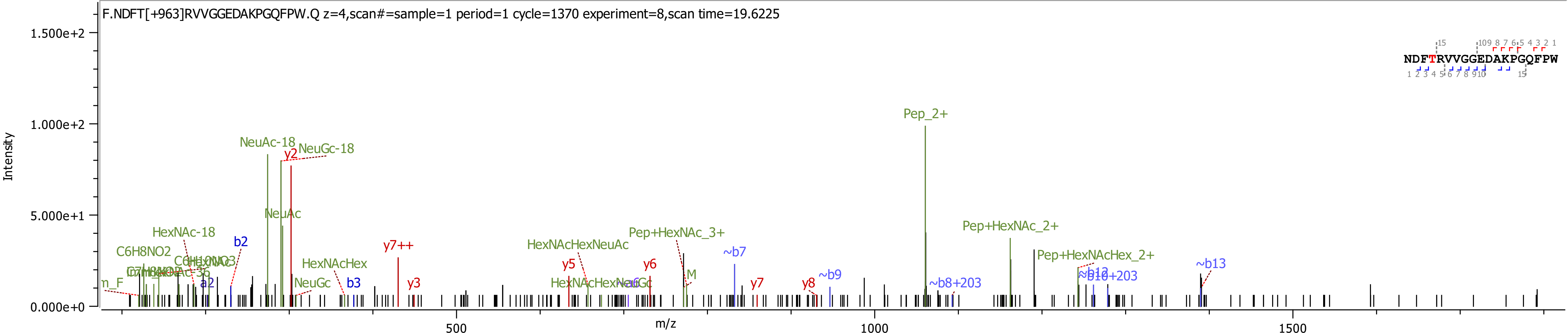

F.NDFT[+963]RVVGGEDAKPGQFPW.Q z=4,scan#=sample=1 period=1 cycle=1370 experiment=8,scan time=19.6225
Intensity
m/z
1.500e+2
1.000e+2
5.000e+1
0.000e+0
500
1000
1500
Pep_2+
NeuAc-18
NeuGc-18
y2
NeuAc
HexNAc-18
C6H8NO2
b2
a2
b3
NeuGc
HexNAcHex
y7++
y3
y5
y6
HexNAcHexNeuAc
Pep+HexNAc_3+
~b7
y7
y8
~b9
~b8+203
Pep+HexNAc_2+
Pep+HexNAcHex_2+
~b10+203
~b13
NDFTRVVGGEDAKPGQFPW

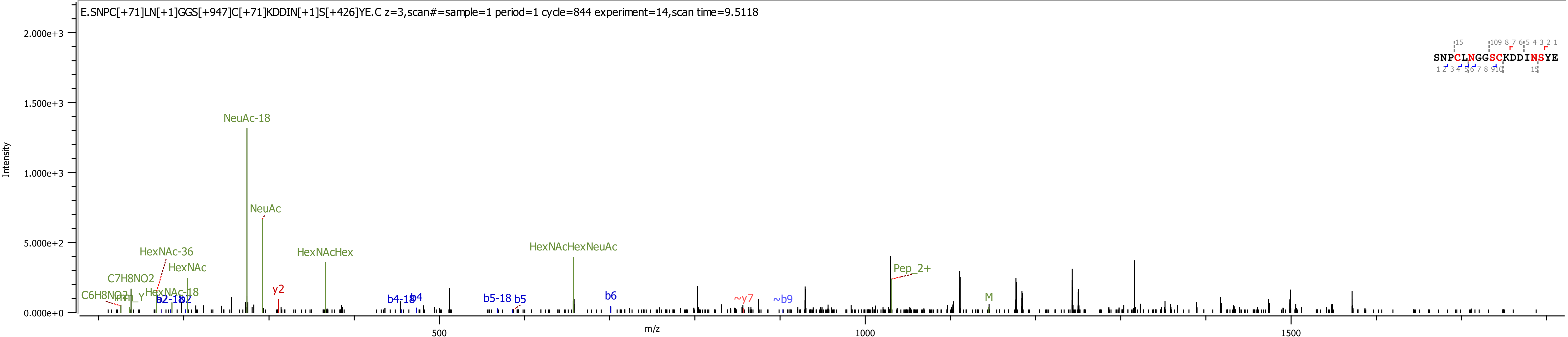
E.SNPC[+71]LN[+1]GGS[+947]C[+71]KDDIN[+1]S[+426]YE.C z=3,scan#=sample=1 period=1 cycle=844 experiment=14,scan time=9.5118
SNPCLNGGSCKDDINSYE
Intensity
m/z
NeuAc-18
NeuAc
HexNAcHex
HexNAcHexNeuAc
HexNAc-36
HexNAc
C7H8NO2
HexNAc-18
C6H8NO2
Imm_Y
b2-18
b2
y2
b4-18
b4
b5-18
b5
b6
~y7
~b9
Pep_2+
M
0.000e+0
5.000e+2
1.000e+3
1.500e+3
2.000e+3
500
1000
1500

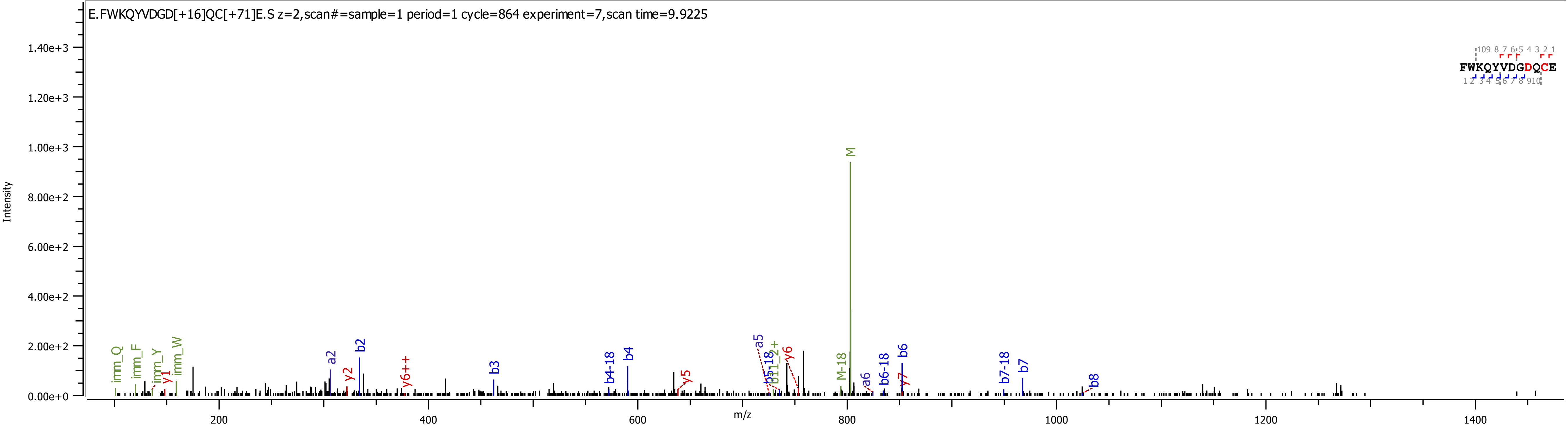
E.FWKQYVDGD[+16]QC[+71]E.S z=2,scan#=sample=1 period=1 cycle=864 experiment=7,scan time=9.9225
Intensity
m/z
FWKQYVDGDQCE
M
M-18
imm_Q
imm_F
imm_Y
imm_W
y1
y2
y5
y6
y7
y6++
a2
a5
a6
b2
b3
b4
b4-18
b6
b6-18
b7
b7-18
b8
b11_2+

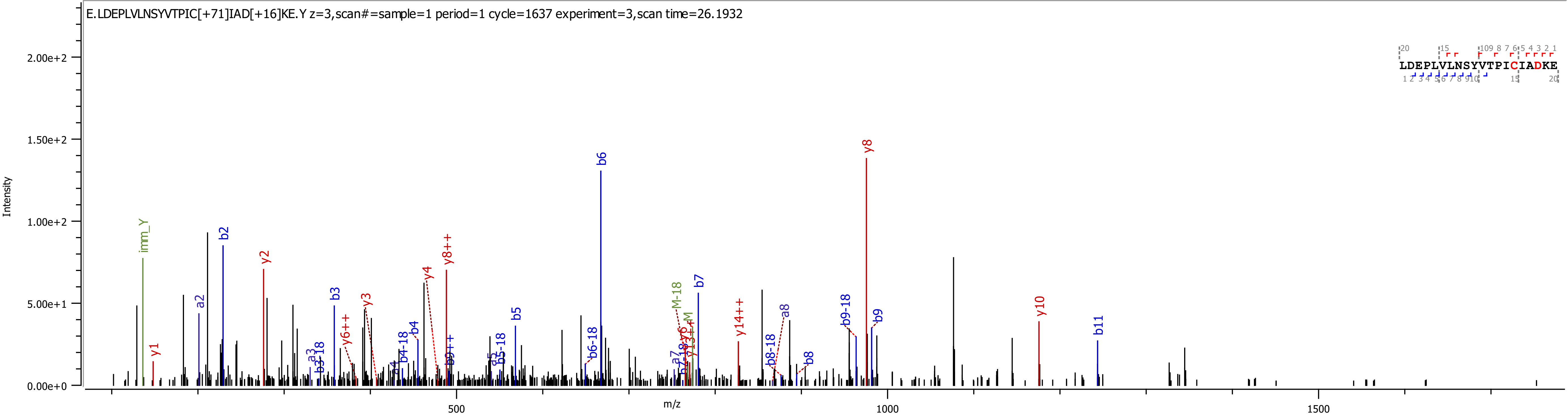
E.LDEPLVLNSYVTPIC[+71]IAD[+16]KE.Y z=3,scan#=sample=1 period=1 cycle=1637 experiment=3,scan time=26.1932
LDEPLVLNSYVTPICIADKE
Intensity
m/z
b6
y8
b2
y2
y8++
y4
imm_Y
b7
M-18
y14++
y10
b11
b9-18
b9
a8
b3
y3
a2
b5
y1

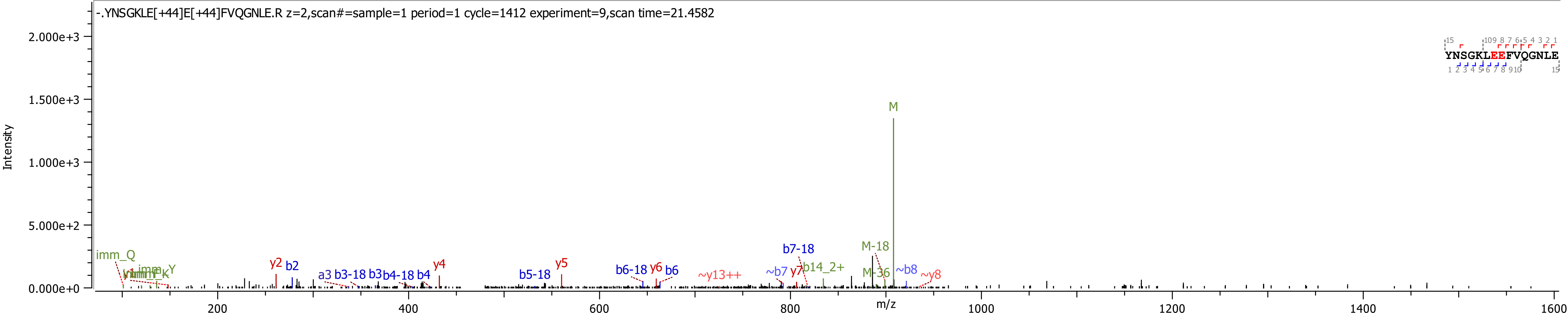
-.YNSGKLE[+44]E[+44]FVQGNLE.R z=2,scan#=sample=1 period=1 cycle=1412 experiment=9,scan time=21.4582
Intensity
m/z
YNSGKLEEFVQGNLE
M
M-18
M-36
y2
y4
y5
y6
y7
~y8
~y13++
b2
a3
b3-18
b3
b4-18
b4
b5-18
b6-18
b6
b7-18
~b7
~b8
b14_2+
imm_Q
imm_Y
imm_K

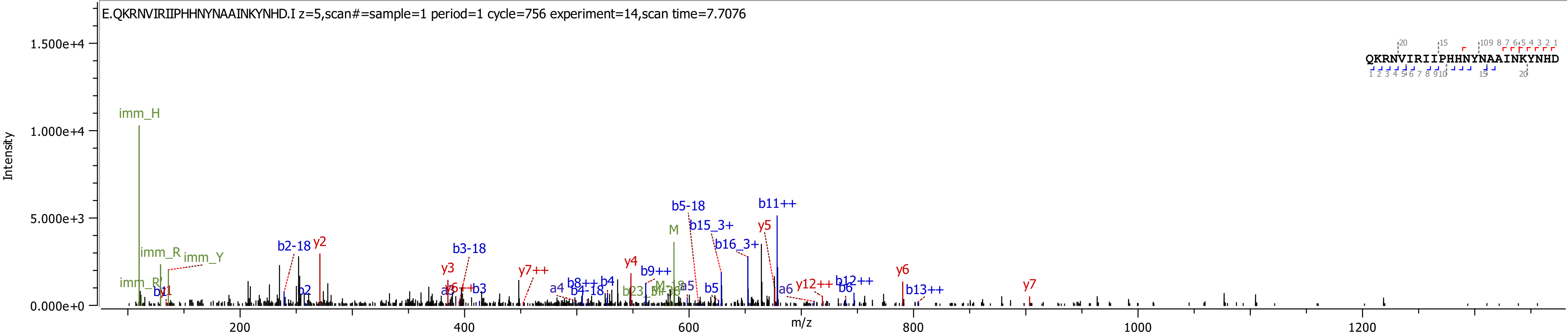

E.QKRNVIRIIPHHNYNAAINKYNHD.I z=5,scan#=sample=1 period=1 cycle=756 experiment=14,scan time=7.7076
QKRNVIRIIPHHNYNAAINKYNHD
Intensity
m/z
1.500e+4
1.000e+4
5.000e+3
0.000e+0
200
400
600
800
1000
1200
imm_H
imm_R
imm_Y
b2-18
y2
b3-18
y3
y7++
y4
b9++
M
b5-18
b15_3+
b16_3+
b11++
y5
y12++
b12++
y6
b13++
y7
a4
b8++
b4
a5
b5
a6
b6

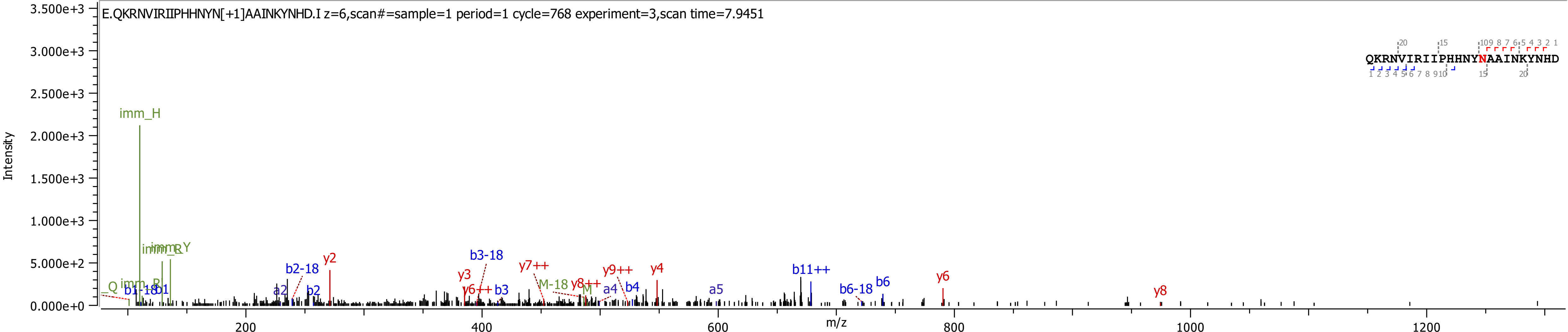
E.QKRNVIRIIPHHNYN[+1]AAINKYNHD.I z=6,scan#=sample=1 period=1 cycle=768 experiment=3,scan time=7.9451
QKRNVIRIIPHHNYNAAINKYNHD
Intensity
m/z
3.500e+3
3.000e+3
2.500e+3
2.000e+3
1.500e+3
1.000e+3
5.000e+2
0.000e+0
200
400
600
800
1000
1200
imm_H
imm_Y
imm_R
Q
b1-18
b1
a2
b2-18
b2
y2
y3
b3-18
y6++
b3
y7++
M-18
y8++
M
y9++
a4
b4
y4
a5
b11++
b6-18
b6
y6
y8

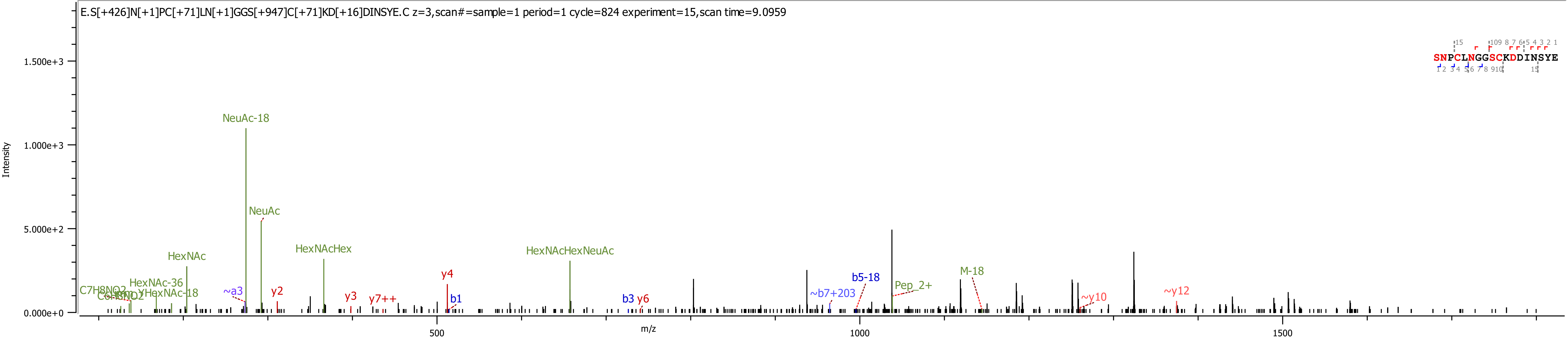
E.S[+426]N[+1]PC[+71]LN[+1]GGS[+947]C[+71]KD[+16]DINSYE.C z=3,scan#=sample=1 period=1 cycle=824 experiment=15,scan time=9.0959
SNPCLNGGSCKDDINSYE
Intensity
1.500e+3
1.000e+3
5.000e+2
0.000e+0
NeuAc-18
NeuAc
HexNAc
HexNAcHex
HexNAcHexNeuAc
HexNAc-36
HexNAc-18
C7H8NO2
~a3
y2
y3
y7++
y4
b1
b3
y6
~b7+203
b5-18
Pep_2+
M-18
~y10
~y12
500
1000
1500
m/z

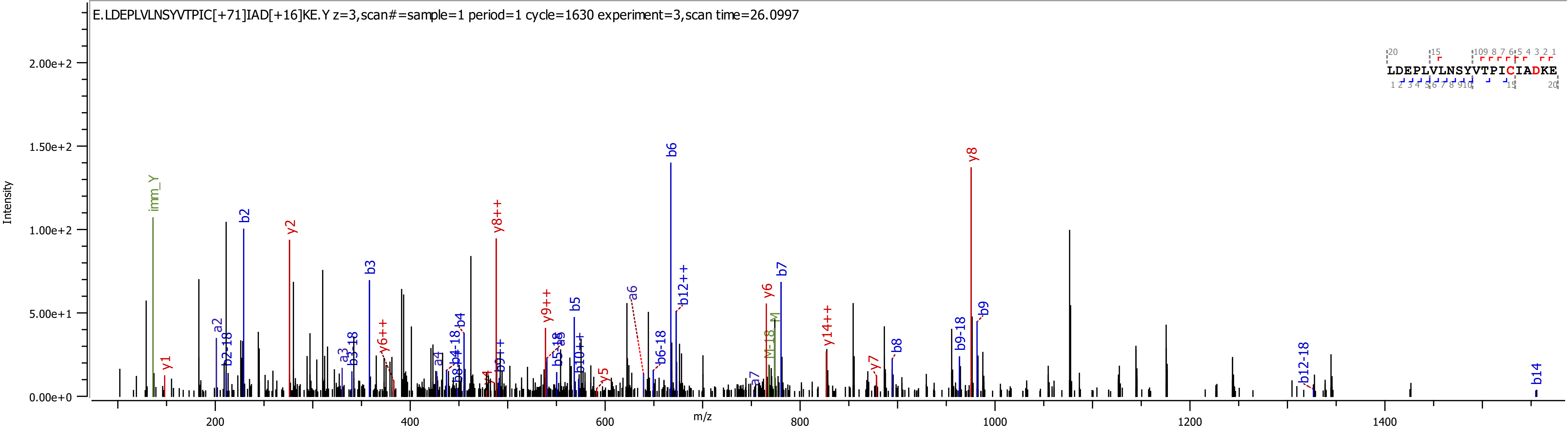
E.LDEPLVLNSYVTPIC[+71]IAD[+16]KE.Y z=3,scan#=sample=1 period=1 cycle=1630 experiment=3,scan time=26.0997
LDEPLVLNSYVTPICIADKE
Intensity
m/z
2.00e+2
1.50e+2
1.00e+2
5.00e+1
0.00e+0
200
400
600
800
1000
1200
1400
imm_Y
y1
a2
b2-18
b2
y2
a3
b3-18
b3
y6++
a4
b8-b4-18
b4
y4
y8++
b9++
y9++
b5-18
a5
b5
b10++
y5
a6
b6-18
b6
b12++
a7
M-18
M
y6
b7
y14++
y7
b8
b9-18
b9
y8
b12-18
b14

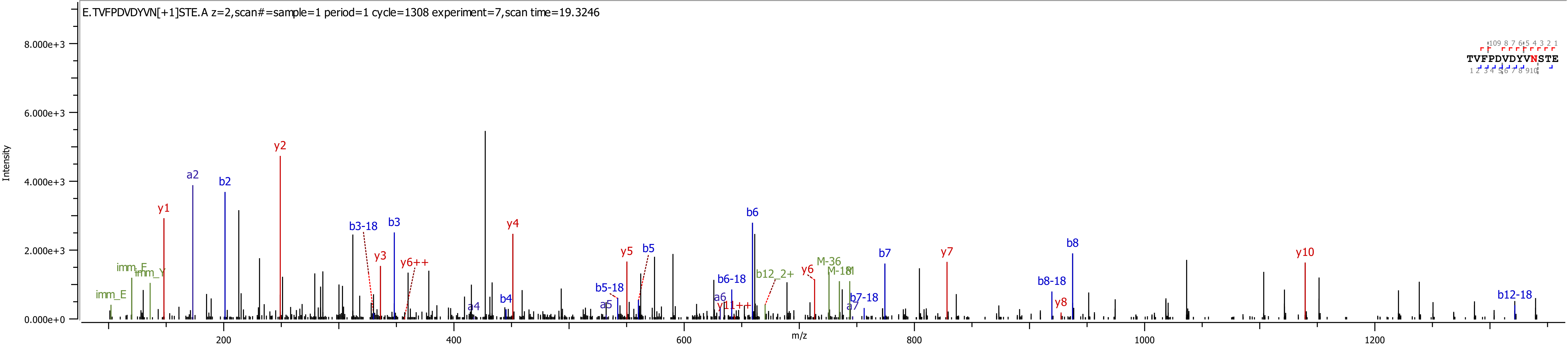
E.TVFPDVDYVN[+1]STE.A z=2,scan#=sample=1 period=1 cycle=1308 experiment=7,scan time=19.3246
Intensity
m/z
TVFPDVDYVNSTE
y1
a2
b2
y2
b3-18
b3
y3
y6++
y4
a4
b4
b5-18
a5
y5
b5
b6-18
a6
y11++
b6
b12_2+
y6
M-36
M-18
M
b7-18
a7
b7
y7
b8-18
y8
b8
y10
b12-18
imm_E
imm_Y
0.000e+0
2.000e+3
4.000e+3
6.000e+3
8.000e+3
200
400
600
800
1000
1200

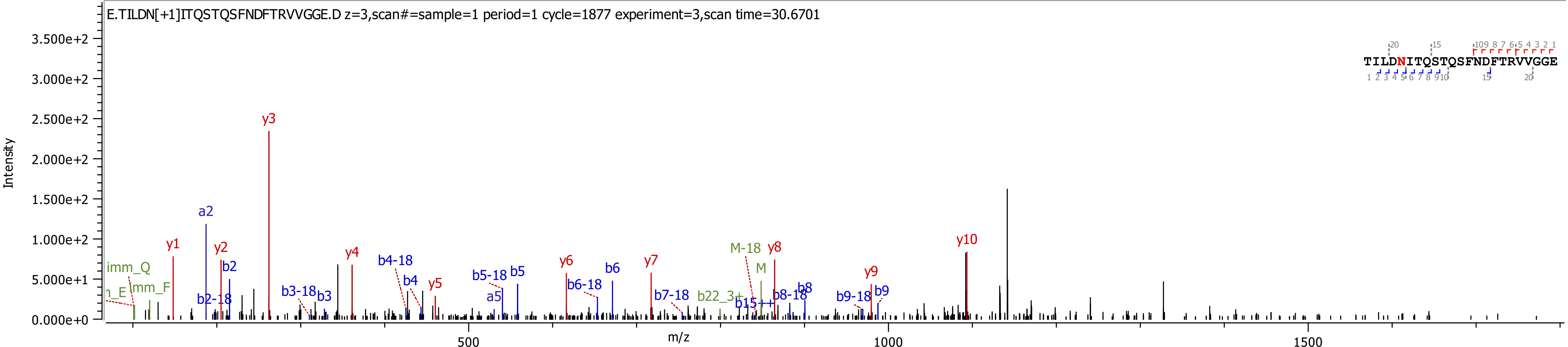

E.TILDN[+1]ITQSTQSFNDFTRVVGGE.D z=3,scan#=sample=1 period=1 cycle=1877 experiment=3,scan time=30.6701
Intensity
m/z
TILDNITQSTQSFNDFTRVVGGE
y1
y2
y3
y4
y5
y6
y7
y8
y9
y10
a2
a5
b2
b2-18
b3
b3-18
b4
b4-18
b5
b5-18
b6
b6-18
b7-18
b8
b8-18
b9
b9-18
b15++
b22_3+
imm_Q
imm_F
M
M-18

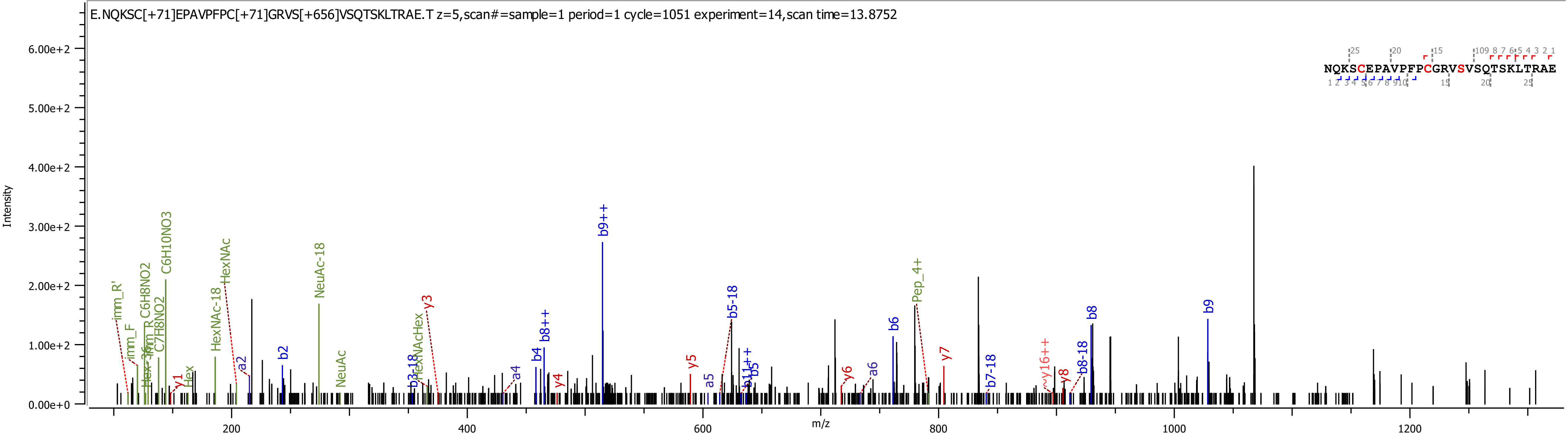
E.NQKSC[+71]EPAVPFPC[+71]GRVS[+656]VSQTSKLTRAE.T z=5,scan#=sample=1 period=1 cycle=1051 experiment=14,scan time=13.8752
NQKSCEPAVPFPCGRVSVSQTSKLTRAE
Intensity
6.00e+2
5.00e+2
4.00e+2
3.00e+2
2.00e+2
1.00e+2
0.00e+0
m/z
200
400
600
800
1000
1200
imm_R'
imm_F
Hex-36
imm_R
C6H8NO2
C7H8NO2
C6H10NO3
y1
Hex
HexNAc-18
HexNAc
a2
b2
NeuAc-18
NeuAc
b3-18
HexNAcHex
y3
y4
a4
b4
b8++
b9++
y5
a5
b5-18
b11++
b5
y6
a6
b6
Pep_4+
y7
b7-18
y16++
y8
b8-18
b8
b9

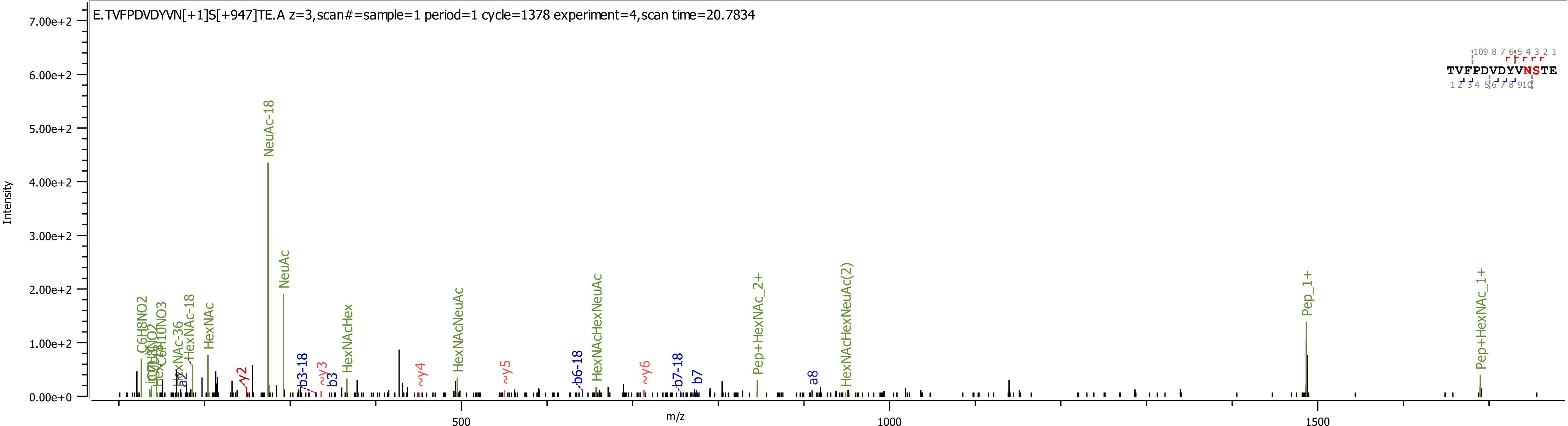
E.TVFPDVDYVN[+1]S[+947]TE.A z=3,scan#=sample=1 period=1 cycle=1378 experiment=4,scan time=20.7834
TVFPDVDYVNSTE
Intensity
m/z
7.00e+2
6.00e+2
5.00e+2
4.00e+2
3.00e+2
2.00e+2
1.00e+2
0.00e+0
500
1000
1500
NeuAc-18
NeuAc
HexNAc
HexNAc-18
HexNAc-36
C6H8NO2
C6H10NO3
y2
b3-18
~y3
b3
HexNAcHex
~y4
HexNAcNeuAc
~y5
b6-18
HexNAcHexNeuAc
~y6
b7-18
b7
Pep+HexNAc_2+
a8
HexNAcHexNeuAc(2)
Pep_1+
Pep+HexNAc_1+

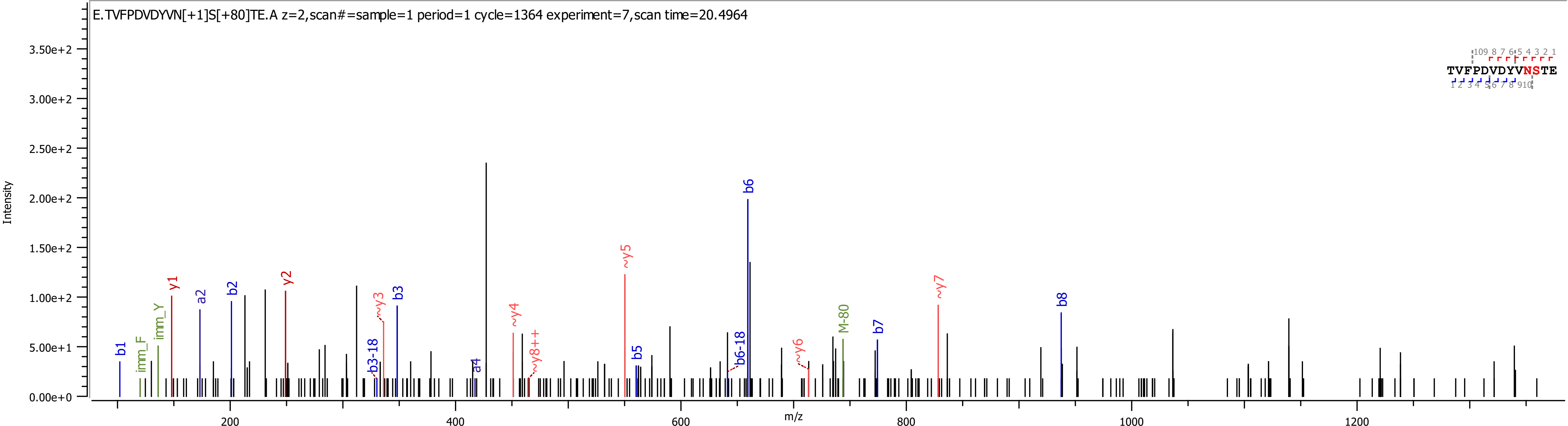

E.TVFPDVDYVN[+1]S[+80]TE.A z=2,scan#=sample=1 period=1 cycle=1364 experiment=7,scan time=20.4964
Intensity
3.50e+2
3.00e+2
2.50e+2
2.00e+2
1.50e+2
1.00e+2
5.00e+1
0.00e+0
m/z
200
400
600
800
1000
1200
TVFPDVDYVNSTE
b1
imm_F
imm_Y
y1
a2
b2
y2
b3-18
~y3
b3
a4
~y4
~y8++
~y5
b5
b6-18
b6
~y6
M-80
b7
~y7
b8

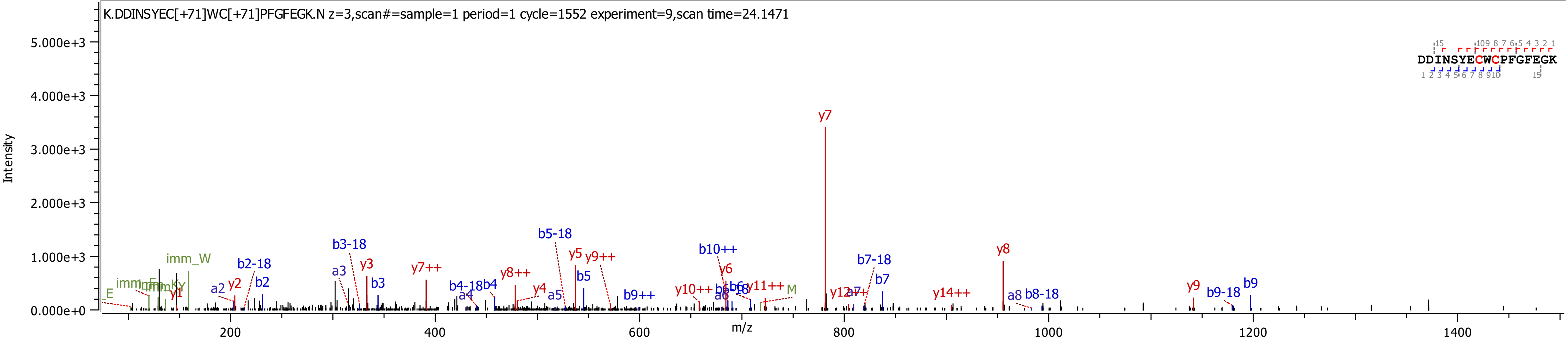

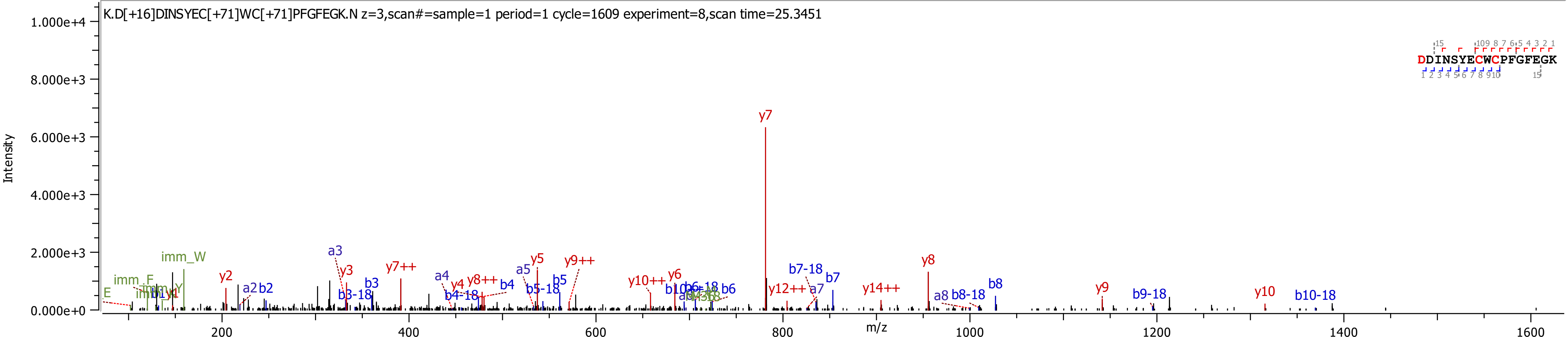
K.D[+16]DINSYEC[+71]WC[+71]PFGFEGK.N z=3,scan#=sample=1 period=1 cycle=1609 experiment=8,scan time=25.3451
DDINSYECWCPFGFEGK
Intensity
m/z
1.000e+4
8.000e+3
6.000e+3
4.000e+3
2.000e+3
0.000e+0
200
400
600
800
1000
1200
1400
1600
y7
y8
y5
y9++
a3
y3
y7++
y2
imm_W
imm_F
E
a2
b2
b3
b3-18
a4
y4
y8++
b4
b4-18
a5
b5
b5-18
y10++
y6
b6-18
b6
b7-18
b7
a7
y12++
y14++
a8
b8-18
b8
y9
b9-18
y10
b10-18

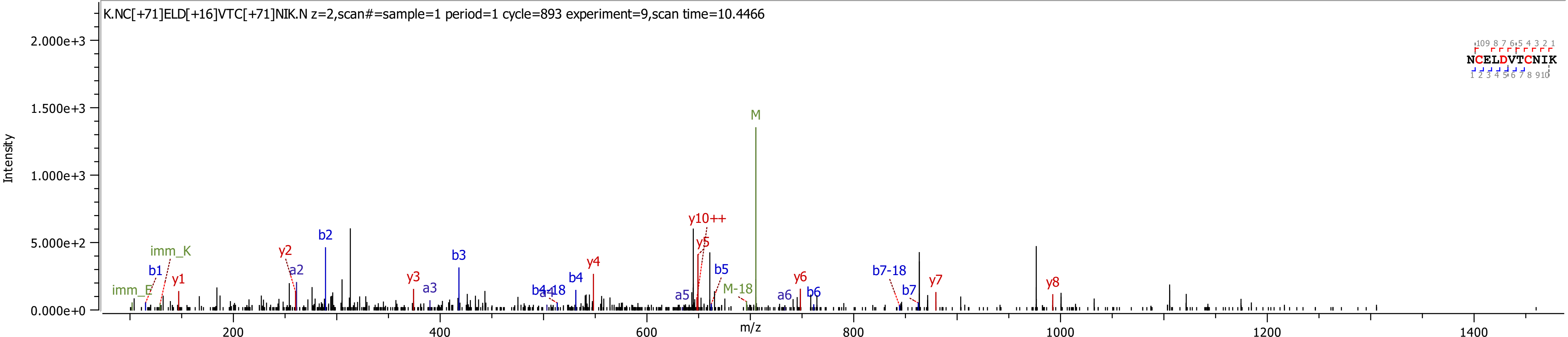

K.NC[+71]ELD[+16]VTC[+71]NIK.N z=2,scan#=sample=1 period=1 cycle=893 experiment=9,scan time=10.4466
NCELDVTCNIK
Intensity
m/z
M
y10++
y5
b5
M-18
y6
b6
a6
b7-18
b7
y7
y8
imm_K
imm_E
b1
y1
y2
a2
b2
b3
a3
y3
b4-18
a4
b4
y4
a5

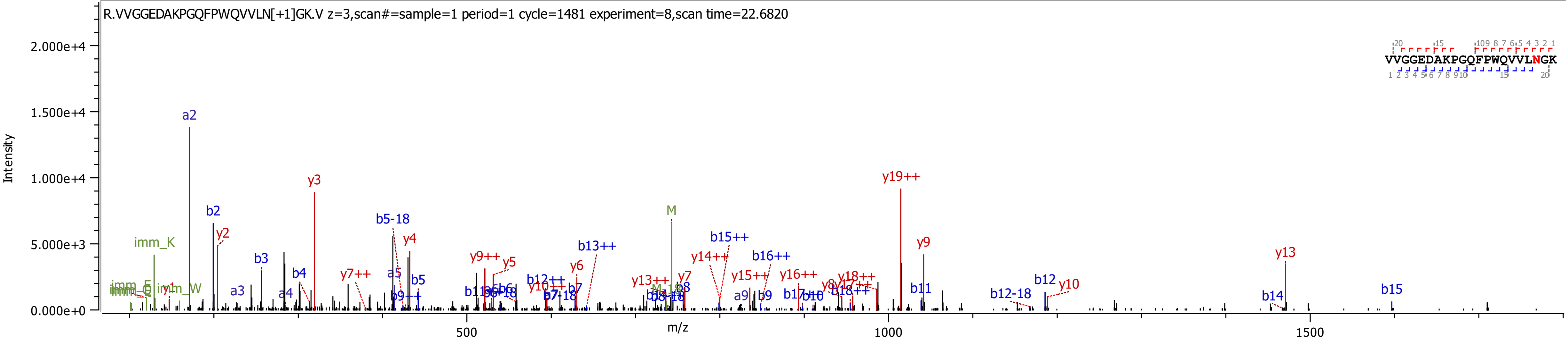

R.VVGGEDAKPGQFPWQVVLN[+1]GK.V z=3,scan#=sample=1 period=1 cycle=1481 experiment=8,scan time=22.6820
VVGGEDAKPGQFPWQVVLNGK
Intensity
2.000e+4
1.500e+4
1.000e+4
5.000e+3
0.000e+0
m/z
500
1000
1500
a2
b2
y2
imm_K
y1
imm_W
a3
b3
a4
b4
y3
y7++
a5
b5-18
y4
b5
b9++
y9++
y5
b12++
y10++
y6
b7
b7-18
b13++
M
y13++
y7
b8
y14++
b15++
a9
b9
y15++
b16++
y16++
b17++
b10
y18++
b18++
y19++
y9
b11
b12-18
b12
y10
y13
b14
b15

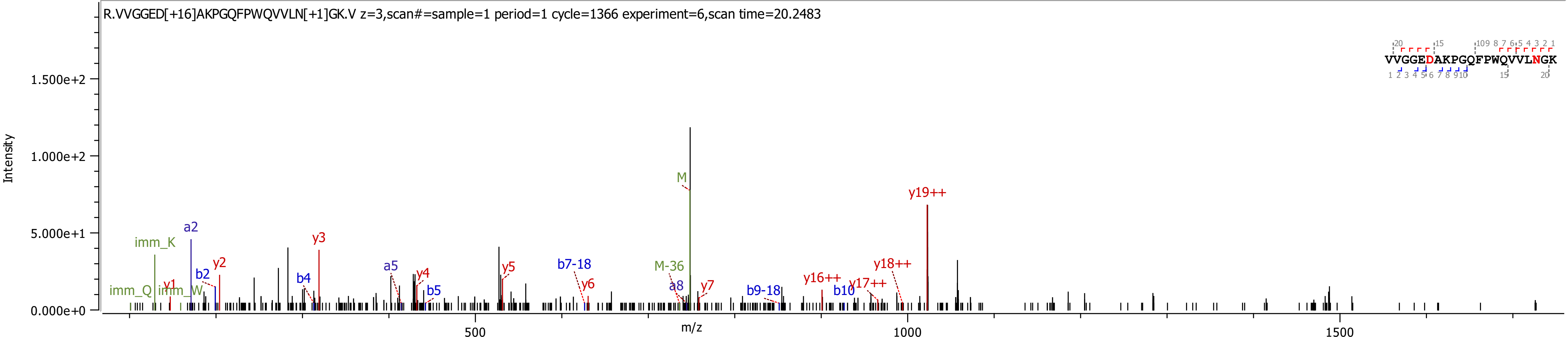
R.VVGGED[+16]AKPGQFPWQVVLN[+1]GK.V z=3,scan#=sample=1 period=1 cycle=1366 experiment=6,scan time=20.2483
VVGGEDAKPGQFPWQVVLNGK
Intensity
1.500e+2
1.000e+2
5.000e+1
0.000e+0
m/z
500
1000
1500
M
y19++
imm_K
a2
y3
y2
b2
y1
imm_Q
imm_W
b4
a5
y4
b5
y5
b7-18
y6
M-36
a8
y7
b9-18
y16++
b10
y17++
y18++

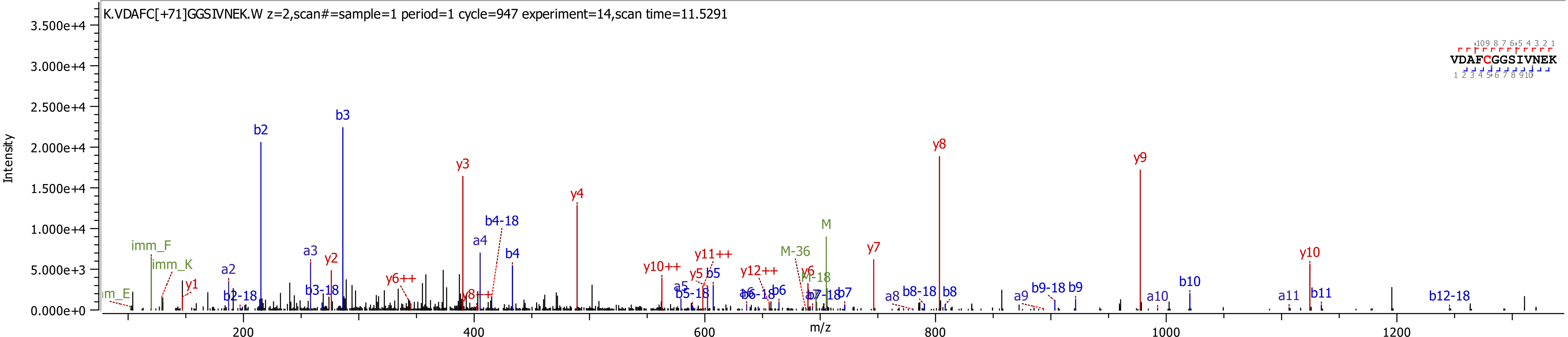
K.VDAFC[+71]GGSIVNEK.W z=2,scan#=sample=1 period=1 cycle=947 experiment=14,scan time=11.5291
VDAFCGGSIVNEK
Intensity
m/z
y8
y9
y3
y4
b2
b3
b4-18
a4
b4
a3
a2
y2
y1
imm_F
imm_K
imm_E
b2-18
b3-18
y6++
y8++
y10++
y11++
y5
b5
a5
b5-18
y12++
a6
b6-18
b6
M
M-36
M-18
y6
a7
b7-18
b7
y7
a8
b8-18
b8
a9
b9-18
b9
a10
b10
y10
a11
b11
b12-18

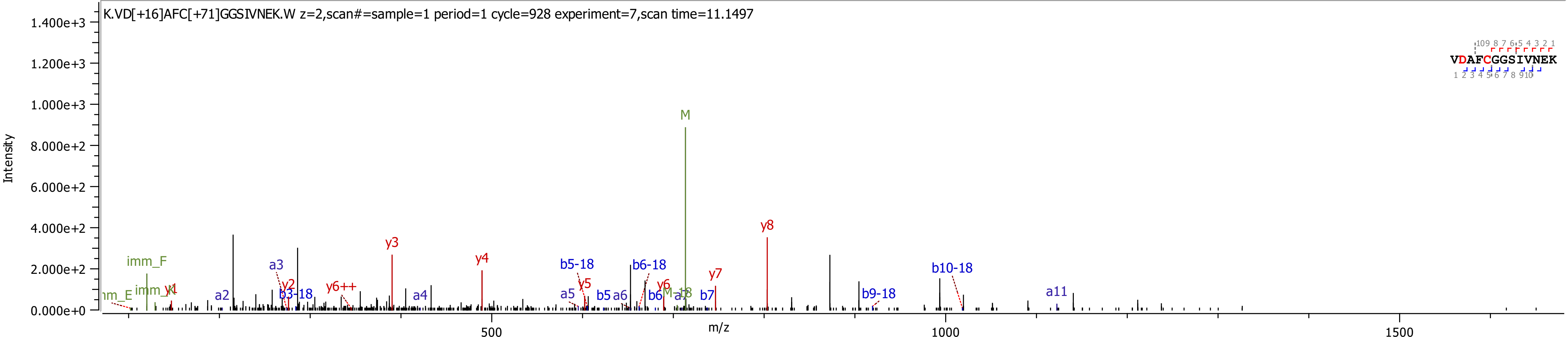
K.VD[+16]AFC[+71]GGSIVNEK.W z=2,scan#=sample=1 period=1 cycle=928 experiment=7,scan time=11.1497
Intensity
m/z
VDAFCGGSIVNEK
M
y8
y3
y4
b5-18
b6-18
b10-18
y7
a3
y2
y6++
a2
b3-18
a4
a5
y5
b5
a6
b6
y6
b7
b9-18
a11
imm_F
imm_E
imm_K
y1

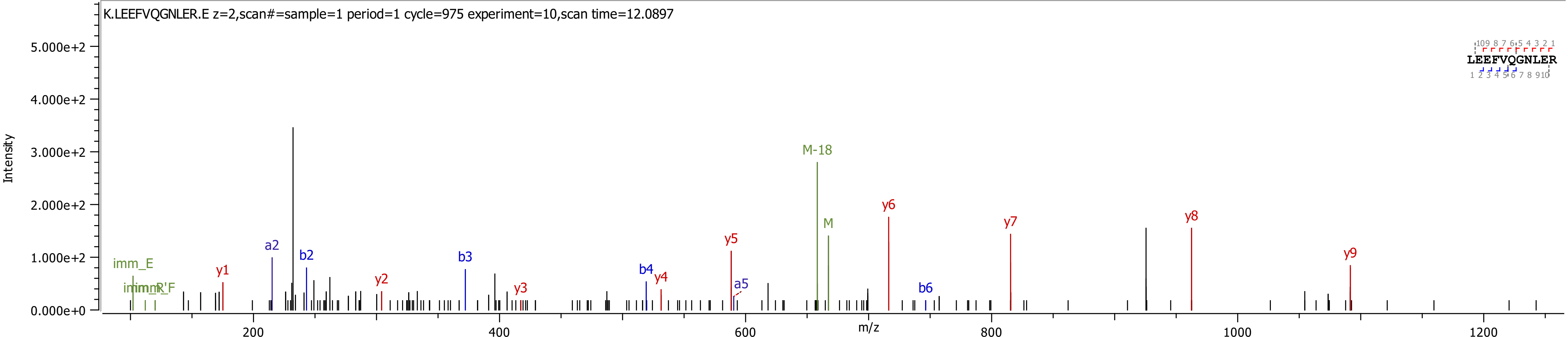
K.LEEFVQGNLER.E z=2,scan#=sample=1 period=1 cycle=975 experiment=10,scan time=12.0897
Intensity
m/z
LEEFVQGNLER
imm_E
y1
a2
b2
b3
y2
y3
b4
y4
y5
a5
M-18
M
y6
b6
y7
y8
y9

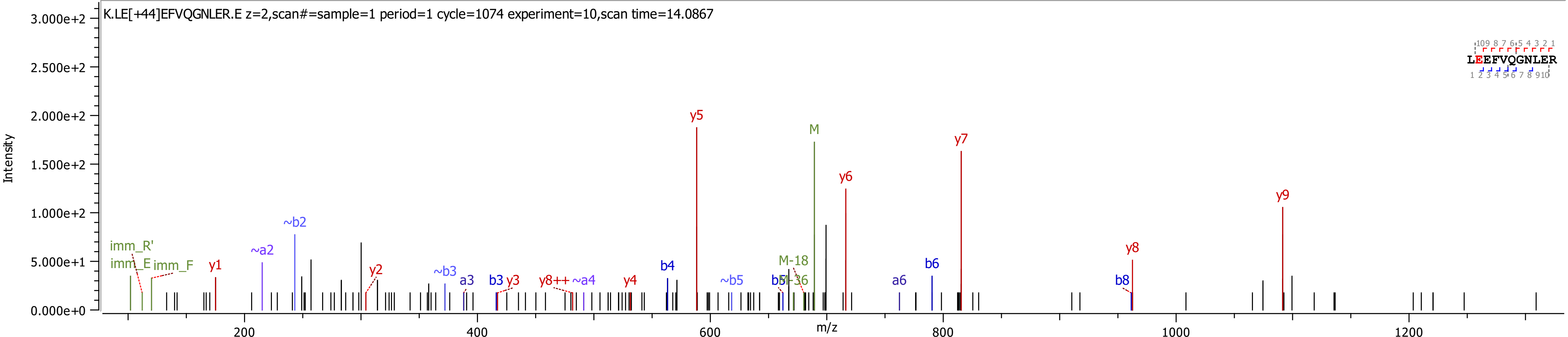

K.LE[+44]EFVQGNLER.E z=2,scan#=sample=1 period=1 cycle=1074 experiment=10,scan time=14.0867
Intensity
m/z
3.000e+2
2.500e+2
2.000e+2
1.500e+2
1.000e+2
5.000e+1
0.000e+0
200
400
600
800
1000
1200
LEEFVQGNLER
imm_R'
imm_E
imm_F
y1
~a2
~b2
y2
~b3
a3
b3
y3
y8++
~a4
y4
b4
y5
~b5
b5
M-36
M-18
M
y6
y7
a6
b6
b8
y8
y9

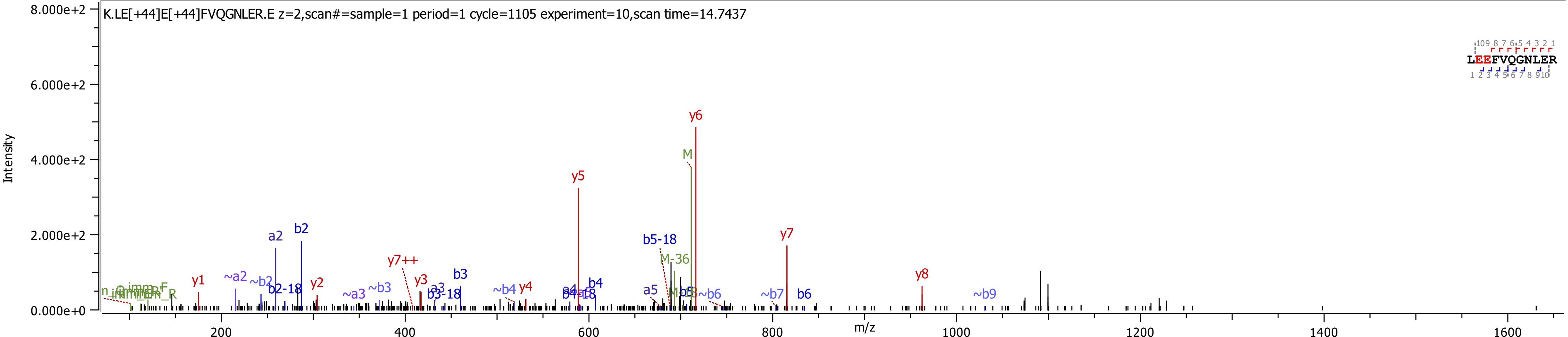
K.LE[+44]E[+44]FVQGNLER.E z=2,scan#=sample=1 period=1 cycle=1105 experiment=10,scan time=14.7437
Intensity
m/z
LEEFVQGNLER
y6
y5
M
y7
b5-18
M-36
a2
b2
y8
y7++
b3
y1
~a2
~b2
b2-18
y2
y3
a3
b3-18
~a3
~b3
~b4
y4
a4
b4
a5
~b6
~b7
b6
~b9
0.000e+0
2.000e+2
4.000e+2
6.000e+2
8.000e+2
200
400
600
800
1000
1200
1400
1600

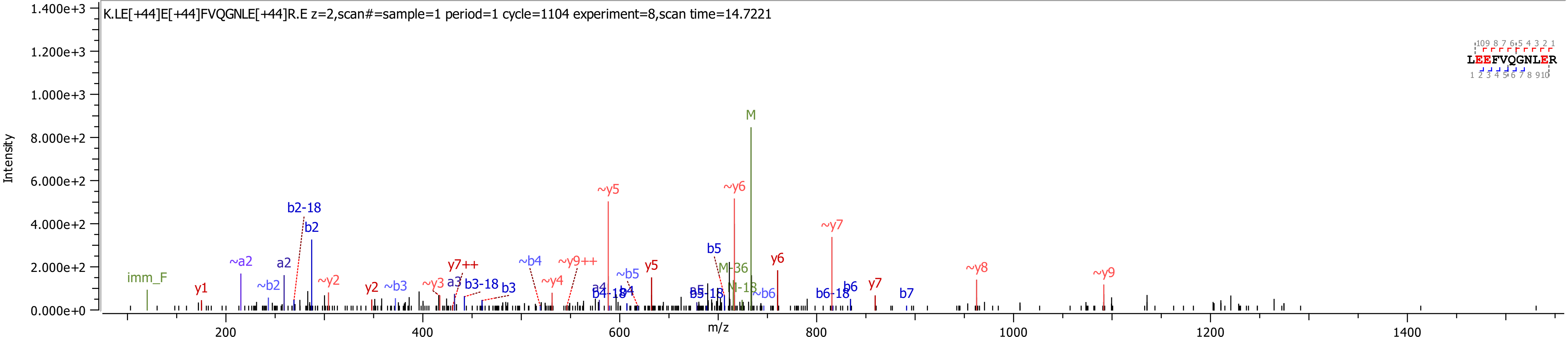
K.LE[+44]E[+44]FVQGNLE[+44]R.E z=2,scan#=sample=1 period=1 cycle=1104 experiment=8,scan time=14.7221
Intensity
m/z
LEEFVQGNLER
imm_F
y1
~a2
~b2
a2
b2-18
b2
~y2
y2
~b3
~y3
a3
y7++
b3-18
b3
~b4
~y4
~y9++
a4
b4-18
b4
~b5
~y5
y5
b5
b5-18
M-36
M-18
~y6
M
~b6
y6
~y7
b6-18
b6
y7
b7
~y8
~y9

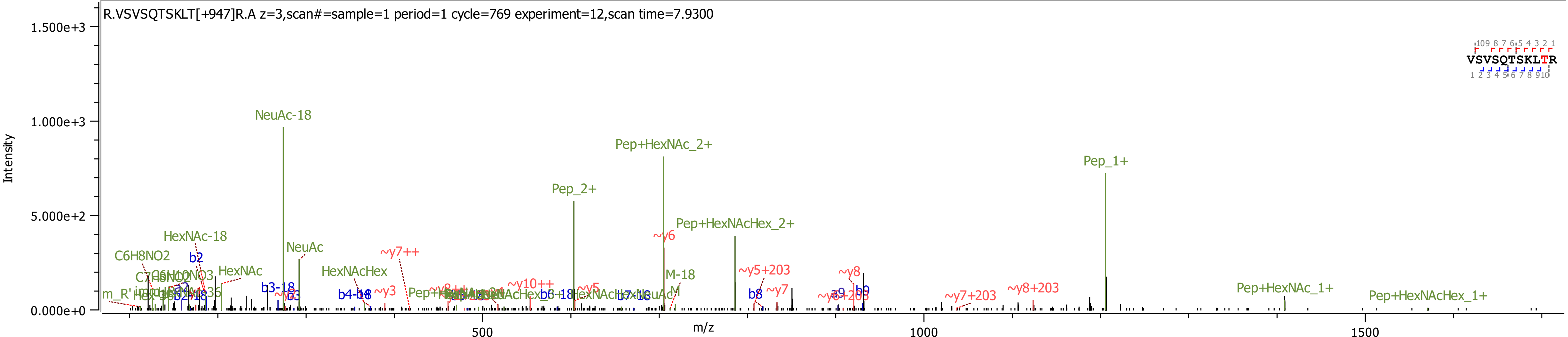
R.VSVSQTSKLT[+947]R.A z=3,scan#=sample=1 period=1 cycle=769 experiment=12,scan time=7.9300
Intensity
1.500e+3
1.000e+3
5.000e+2
0.000e+0
m/z
500
1000
1500
NeuAc-18
Pep+HexNAc_2+
Pep_1+
Pep_2+
Pep+HexNAcHex_2+
~y6
HexNAc-18
NeuAc
C6H8NO2
b2
HexNAc
HexNAcHex
~y7++
M-18
~y5+203
~y8
~y10++
~y5
b3-18
b4-18
~y3
b8
~y7
a9
b9
~y7+203
~y8+203
Pep+HexNAc_1+
Pep+HexNAcHex_1+
VSVSQTSKLTR

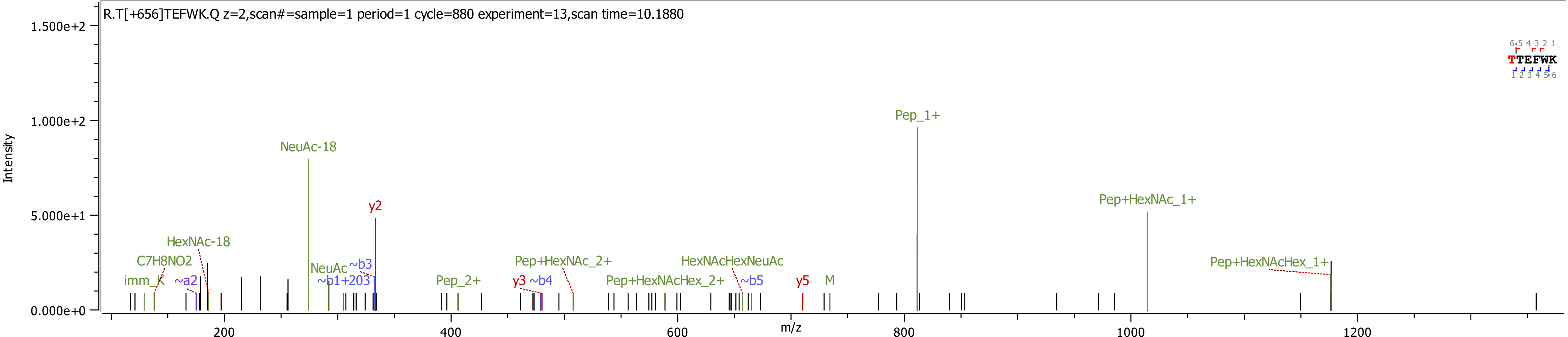
R.T[+656]TEFWK.Q z=2,scan#=sample=1 period=1 cycle=880 experiment=13,scan time=10.1880
Intensity
m/z
TTEFWK
Pep_1+
NeuAc-18
y2
Pep+HexNAc_1+
HexNAc-18
C7H8NO2
imm_K
~a2
NeuAc
~b3
~b1+203
Pep_2+
y3
~b4
Pep+HexNAc_2+
Pep+HexNAcHex_2+
HexNAcHexNeuAc
~b5
y5
M
Pep+HexNAcHex_1+
1.500e+2
1.000e+2
5.000e+1
0.000e+0
200
400
600
800
1000
1200

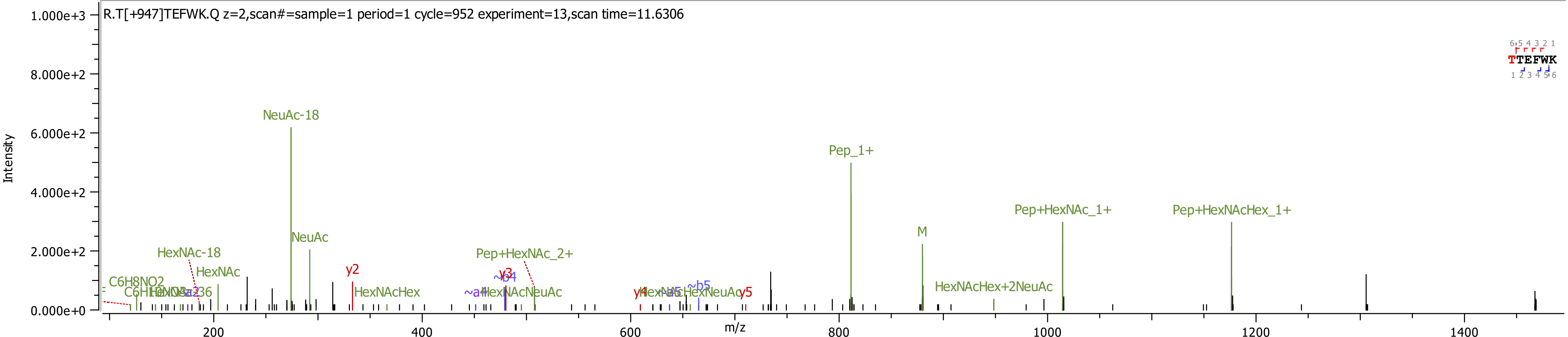
R.T[+947]TEFWK.Q z=2,scan#=sample=1 period=1 cycle=952 experiment=13,scan time=11.6306
Intensity
m/z
1.000e+3
8.000e+2
6.000e+2
4.000e+2
2.000e+2
0.000e+0
200
400
600
800
1000
1200
1400
NeuAc-18
Pep_1+
Pep+HexNAc_1+
Pep+HexNAcHex_1+
M
NeuAc
HexNAc-18
HexNAc
Pep+HexNAc_2+
y2
y3
~b4
~a4
HexNAcHex
HexNAcNeuAc
C6H8NO2
HexNAcHex+2NeuAc
y4
y5
~b5
~a5
HexNAcHexNeuAc
TTEFWK

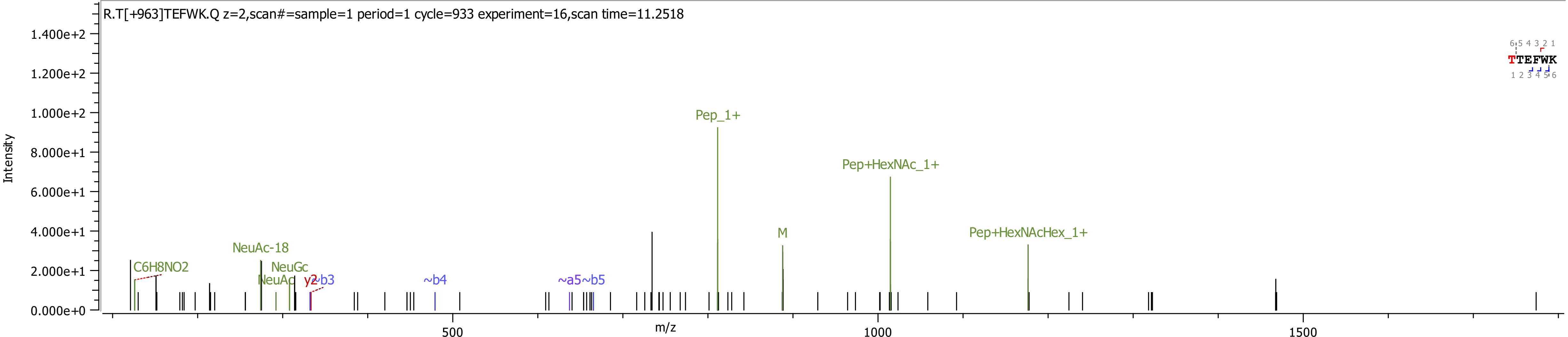
R.T[+963]TEFWK.Q z=2,scan#=sample=1 period=1 cycle=933 experiment=16,scan time=11.2518
Intensity
m/z
1.400e+2
1.200e+2
1.000e+2
8.000e+1
6.000e+1
4.000e+1
2.000e+1
0.000e+0
500
1000
1500
Pep_1+
Pep+HexNAc_1+
M
Pep+HexNAcHex_1+
C6H8NO2
NeuAc-18
NeuGc
NeuAc
y2
~b3
~b4
~a5
~b5
TTEFWK

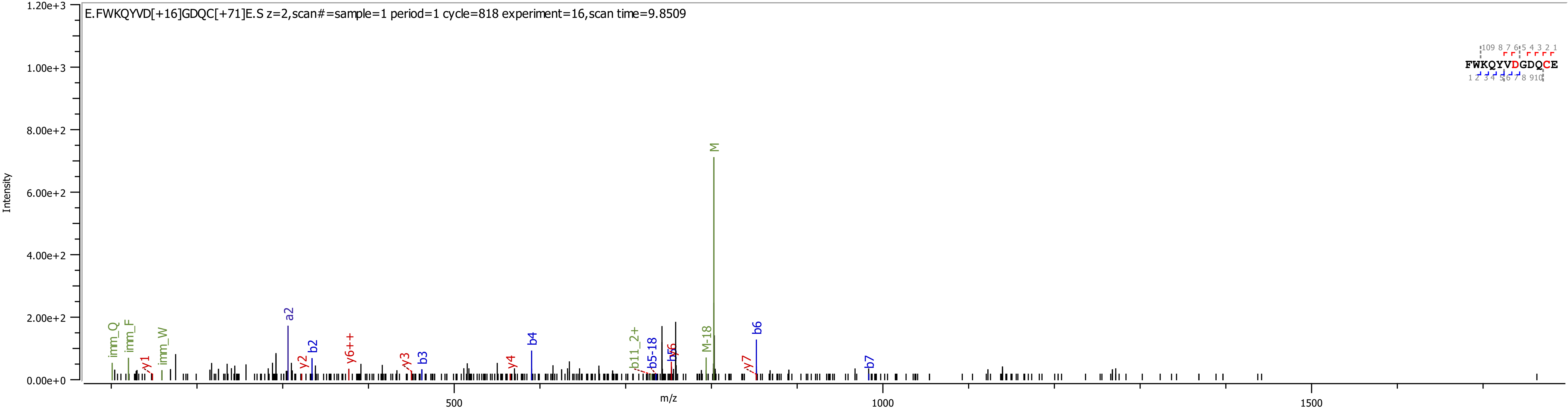

E.FWKQYVD[+16]GDQC[+71]E.S z=2,scan#=sample=1 period=1 cycle=818 experiment=16,scan time=9.8509
FWKQYVDGDQCE
Intensity
m/z
1.20e+3
1.00e+3
8.00e+2
6.00e+2
4.00e+2
2.00e+2
0.00e+0
500
1000
1500
M
M-18
imm_Q
imm_F
imm_W
y1
a2
y2
b2
y6++
y3
b3
y4
b4
b11_2+
b5-18
b5
y5
y7
b6
b7

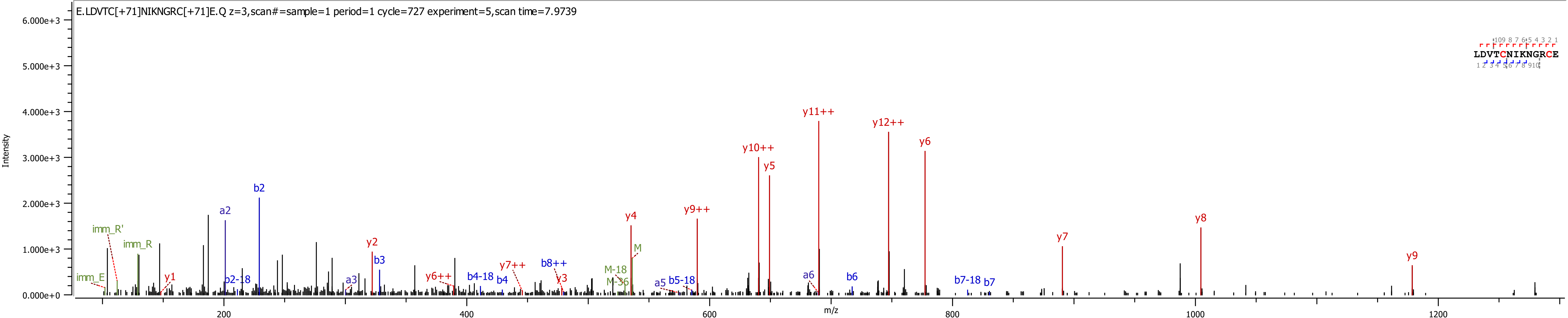

E.LDVTC[+71]NIKNGRC[+71]E.Q z=3,scan#=sample=1 period=1 cycle=727 experiment=5,scan time=7.9739
Intensity
m/z
6.000e+3
5.000e+3
4.000e+3
3.000e+3
2.000e+3
1.000e+3
0.000e+0
200
400
600
800
1000
1200
LDVTCNIKNGRCE
imm_E
imm_R'
imm_R
y1
a2
b2-18
b2
a3
y2
b3
y6++
b4-18
b4
y7++
b8++
y3
M-18
M-36
M
y4
a5
b5-18
y9++
y10++
y5
a6
y11++
b6
y12++
y6
b7-18
b7
y7
y8
y9

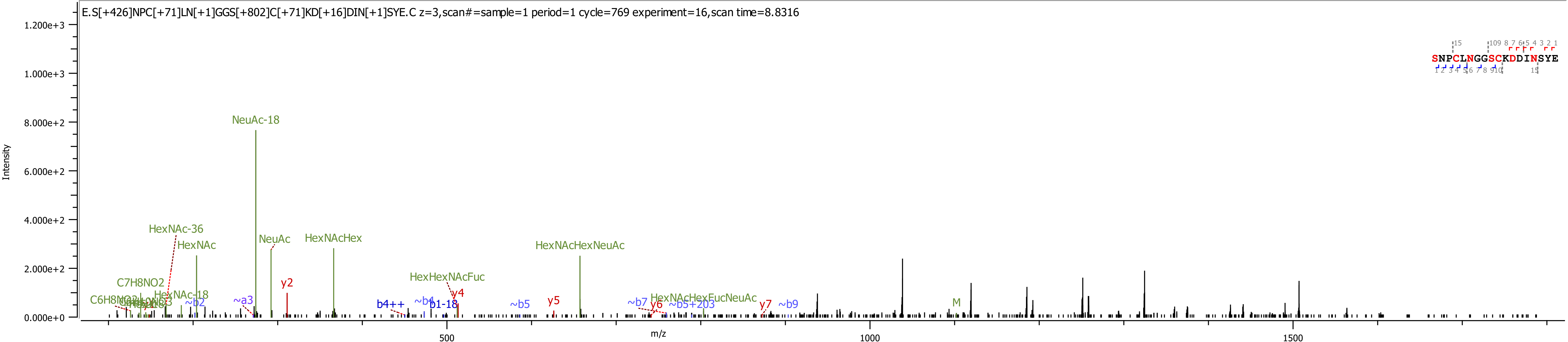

E.S[+426]NPC[+71]LN[+1]GGS[+802]C[+71]KD[+16]DIN[+1]SYE.C z=3,scan#=sample=1 period=1 cycle=769 experiment=16,scan time=8.8316
SNPCLNGGSCKDDINSYE
Intensity
m/z
NeuAc-18
HexNAc-36
HexNAc
NeuAc
HexNAcHex
HexNAcHexNeuAc
HexHexNAcFuc
HexNAcHexFucNeuAc
C7H8NO2
C6H8NO2
HexNAc-18
~b2
~a3
y2
b4++
~b4
b1-18
y4
~b5
y5
~b7
y6
~b5+203
y7
~b9
M
1.200e+3
1.000e+3
8.000e+2
6.000e+2
4.000e+2
2.000e+2
0.000e+0
500
1000
1500

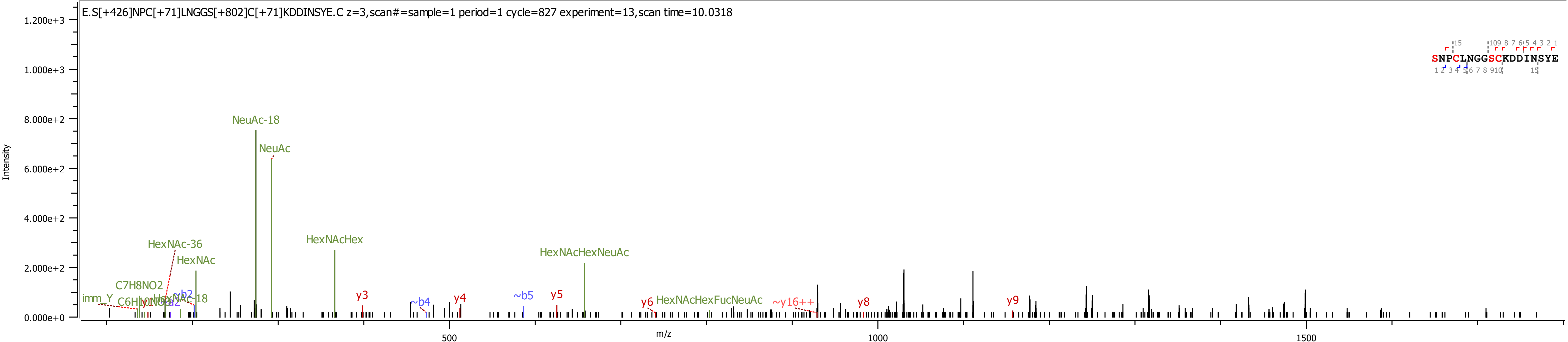

E.S[+426]NPC[+71]LNGGS[+802]C[+71]KDDINSYE.C z=3,scan#=sample=1 period=1 cycle=827 experiment=13,scan time=10.0318
Intensity
m/z
1.200e+3
1.000e+3
8.000e+2
6.000e+2
4.000e+2
2.000e+2
0.000e+0
500
1000
1500
SNPCLNGGSCKDDINSYE
NeuAc-18
NeuAc
HexNAc-36
HexNAc
HexNAcHex
HexNAcHexNeuAc
HexNAcHexFucNeuAc
C7H8NO2
imm_Y
~b2
~b4
~b5
y3
y4
y5
y6
~y16++
y8
y9

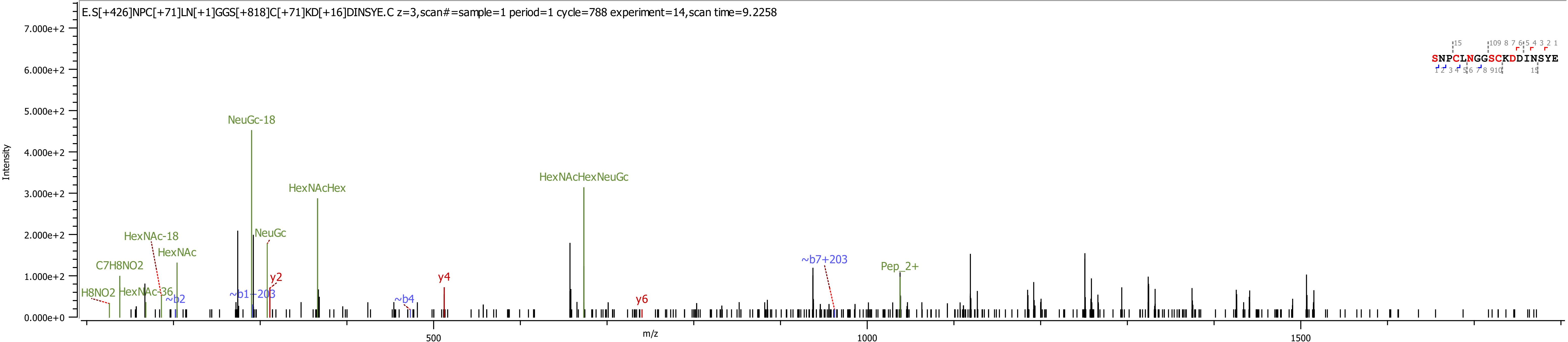

E.S[+426]NPC[+71]LN[+1]GGS[+818]C[+71]KD[+16]DINSYE.C z=3,scan#=sample=1 period=1 cycle=788 experiment=14,scan time=9.2258
Intensity
m/z
SNPCLNGGSCKDDINSYE
NeuGc-18
HexNAcHex
HexNAcHexNeuGc
NeuGc
HexNAc-18
HexNAc
C7H8NO2
H8NO2
HexNAc-36
~b2
~b1+203
y2
~b4
y4
y6
~b7+203
Pep_2+

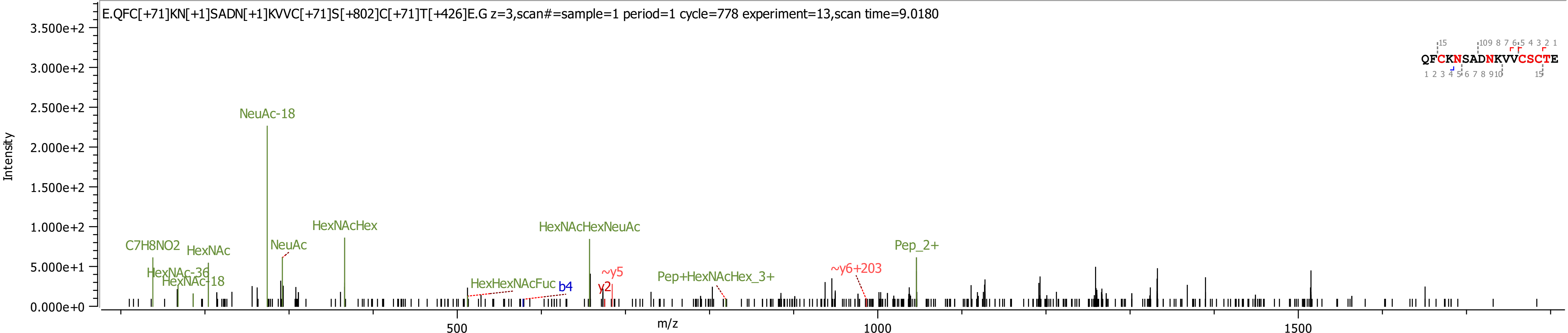
E.QFC[+71]KN[+1]SADN[+1]KVVC[+71]S[+802]C[+71]T[+426]E.G z=3,scan#=sample=1 period=1 cycle=778 experiment=13,scan time=9.0180
Intensity
m/z
NeuAc-18
HexNAcHex
HexNAcHexNeuAc
C7H8NO2
HexNAc
NeuAc
Pep_2+
HexNAc-36
HexNAc-18
HexHexNAcFuc
b4
~y5
y2
Pep+HexNAcHex_3+
~y6+203
QFCKNSADNKVVCSCTE

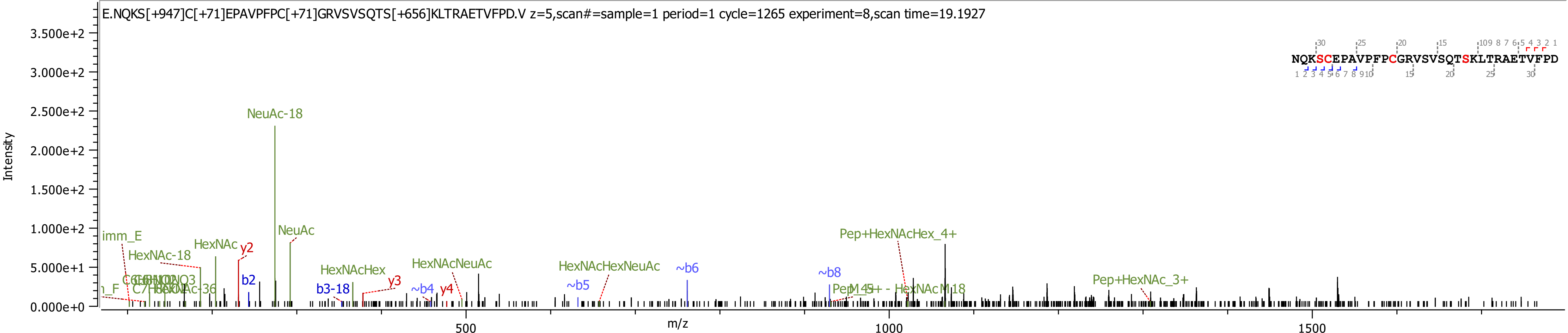
E.NQKS[+947]C[+71]EPAVPFPC[+71]GRVSVSQTS[+656]KLTRAETVFPD.V z=5,scan#=sample=1 period=1 cycle=1265 experiment=8,scan time=19.1927
NQKSCEPAVPFPCGRVSVSQTSKLTRAETVFPD
Intensity
m/z
3.500e+2
3.000e+2
2.500e+2
2.000e+2
1.500e+2
1.000e+2
5.000e+1
0.000e+0
500
1000
1500
NeuAc-18
NeuAc
imm_E
HexNAc-18
HexNAc
y2
b2
HexNAcHex
b3-18
y3
~b4
y4
HexNAcNeuAc
HexNAcHexNeuAc
~b5
~b6
~b8
Pep+HexNAcHex_4+
Pep+HexNAc_3+

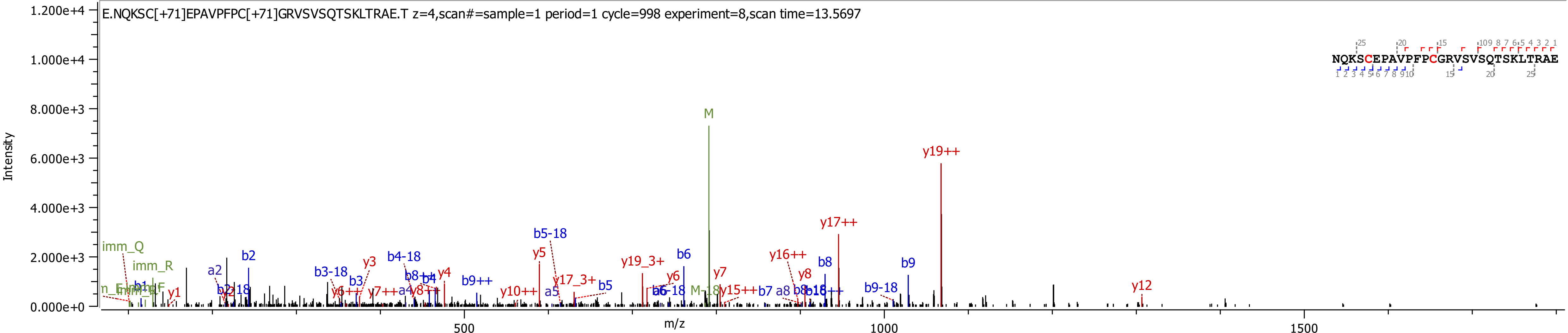
E.NQKSC[+71]EPAVPFPC[+71]GRVSVSQTSKLTRAE.T z=4,scan#=sample=1 period=1 cycle=998 experiment=8,scan time=13.5697
NQKSCEPAVPFPCGRVSVSQTSKLTRAE
Intensity
m/z
M
y19++
y17++
b5-18
y5
b6
b2
imm_Q
imm_R
a2
b3-18
y3
b4-18
y4
b9++
y19_3+
y6
y7
y16++
b8
y8
b9
y12
y15++
b7
b9-18
y17_3+
b5
a5
y10++
y1

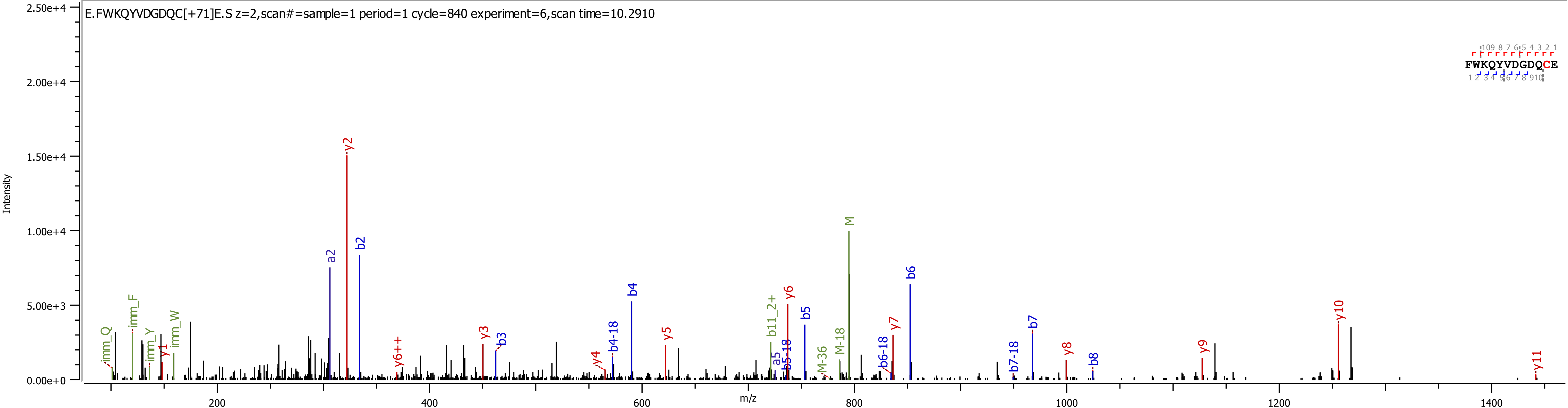
E.FWKQYVDGDQC[+71]E.S z=2,scan#=sample=1 period=1 cycle=840 experiment=6,scan time=10.2910
Intensity
m/z
FWKQYVDGDQCE
y2
a2
b2
M
b6
b4
y6
b5
b11_2+
y10
y3
b3
y5
y7
b7
y9
y8
b8
y11
imm_Q
imm_F
imm_Y
imm_W
y1
y6++
y4
b4-18
a5
b5-18
M-36
M-18
b6-18
b7-18
2.50e+4
2.00e+4
1.50e+4
1.00e+4
5.00e+3
0.00e+0
200
400
600
800
1000
1200
1400

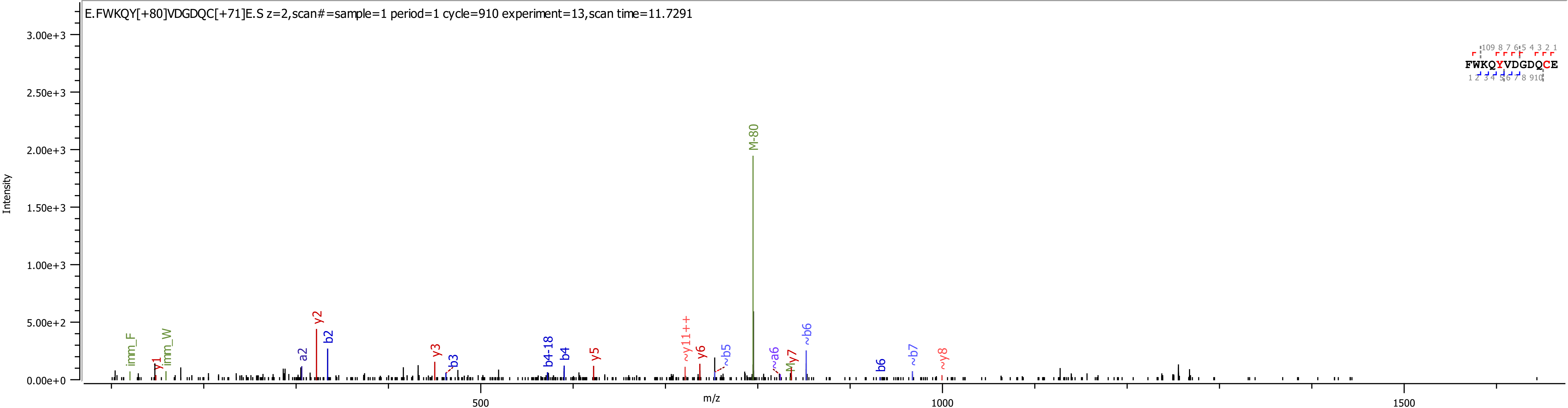
E.FWKQY[+80]VDGDQC[+71]E.S z=2,scan#=sample=1 period=1 cycle=910 experiment=13,scan time=11.7291
Intensity
m/z
FWKQYVDGDQCE
M-80
imm_F
imm_W
y1
a2
y2
b2
y3
b3
b4-18
b4
y5
~y11++
y6
~b5
~a6
y7
~b6
b6
~b7
~y8
3.00e+3
2.50e+3
2.00e+3
1.50e+3
1.00e+3
5.00e+2
0.00e+0
500
1000
1500

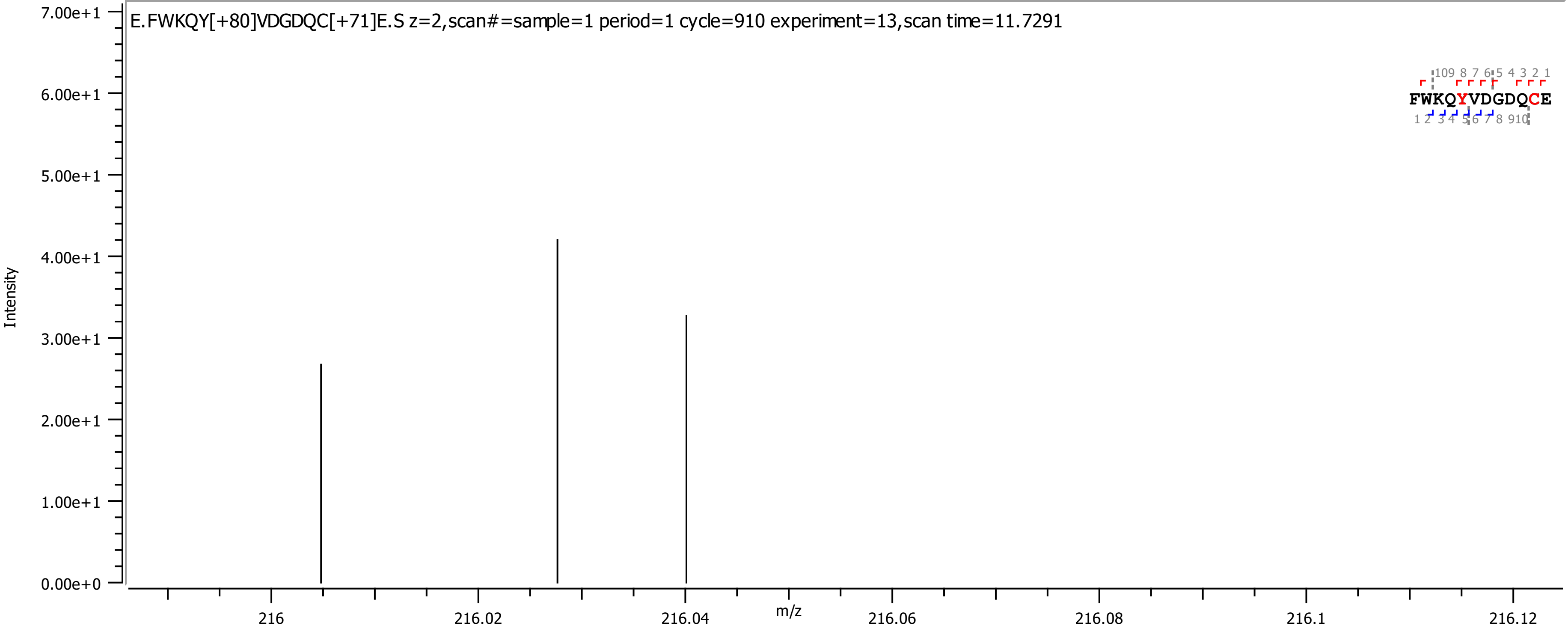
E.FWKQY[+80]VDGDQC[+71]E.S z=2,scan#=sample=1 period=1 cycle=910 experiment=13,scan time=11.7291
FWKQYVDGDQCE
Intensity
m/z
0.00e+0
1.00e+1
2.00e+1
3.00e+1
4.00e+1
5.00e+1
6.00e+1
7.00e+1
216
216.02
216.04
216.06
216.08
216.1
216.12

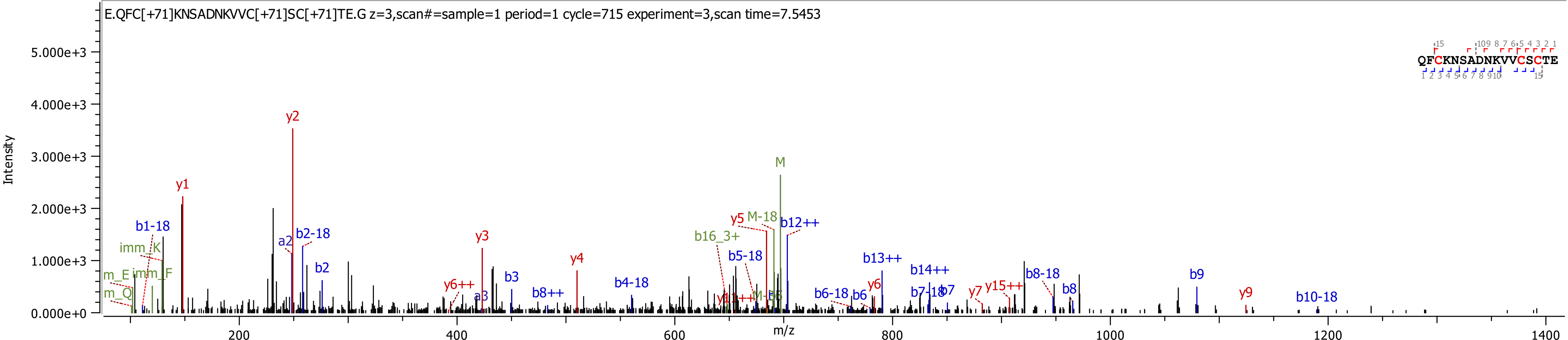
E.QFC[+71]KNSADNKVVC[+71]SC[+71]TE.G z=3,scan#=sample=1 period=1 cycle=715 experiment=3,scan time=7.5453
Intensity
m/z
QFCKNSADNKVVCSCTE
y1
y2
y3
y4
y5
y6
y7
y9
y6++
y15++
b1-18
b2-18
b2
a2
a3
b3
b8++
b4-18
b5-18
b6-18
b6
b7-18
b7
b8-18
b8
b9
b10-18
b12++
b13++
b14++
b16_3+
M
M-18
imm_K
imm_F
m_E
m_Q

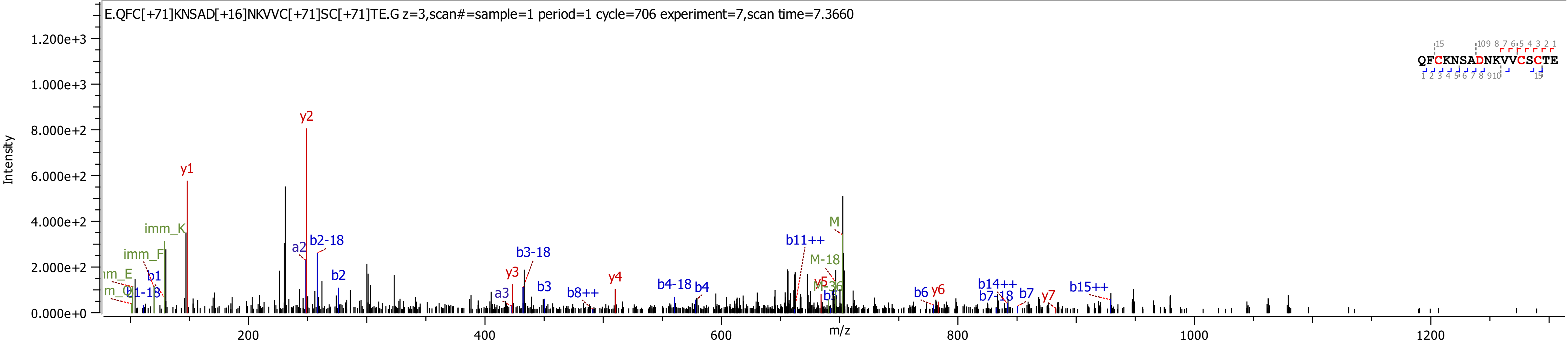

E.QFC[+71]KNSAD[+16]NKVVC[+71]SC[+71]TE.G z=3,scan#=sample=1 period=1 cycle=706 experiment=7,scan time=7.3660
Intensity
m/z
0.000e+0
2.000e+2
4.000e+2
6.000e+2
8.000e+2
1.000e+3
1.200e+3
200
400
600
800
1000
1200
y1
y2
imm_K
imm_F
b1
b1-18
a2
b2-18
b2
b3-18
y3
a3
b3
b8++
y4
b4-18
b4
b11++
M
M-18
y5
b6
y6
b14++
b7-18
b7
y7
b15++
QFCKNSADNKVVCSCTE

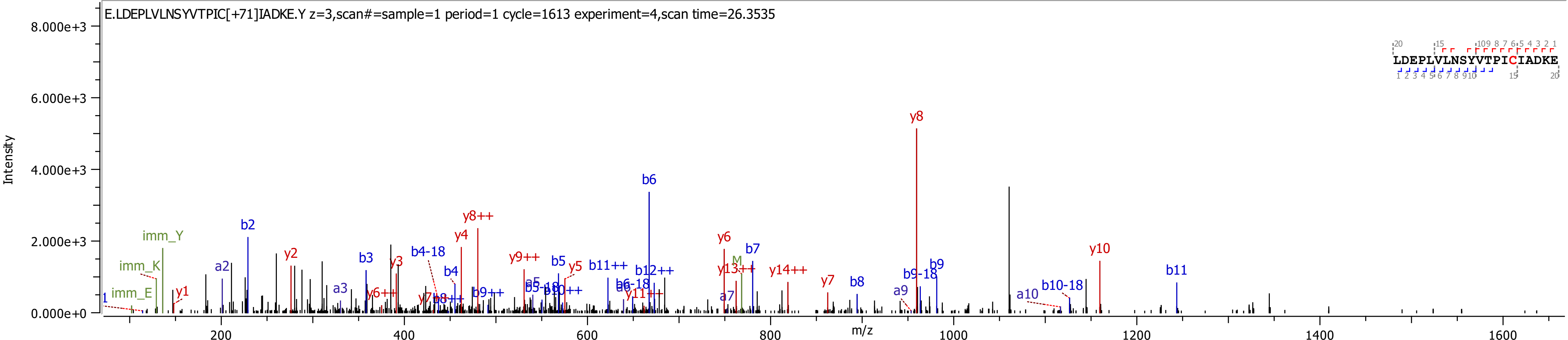
E.LDEPLVLNSYVTPIC[+71]IADKE.Y z=3,scan#=sample=1 period=1 cycle=1613 experiment=4,scan time=26.3535
LDEPLVLNSYVTPICIADKE
Intensity
m/z
y8
b6
y8++
y4
b2
imm_Y
y2
y6
b7
y10
b11
b9
b9-18
b8
y7
y14++
y13++
b12++
b11++
b10-18
a10
a9
a7
b5
y5
y9++
b4
b4-18
b3
y3
a3
a2
imm_K
imm_E
y1
b9++
b8++
y7++
y6++
b10++
b5-18
a5
b6-18
a6
y11++
M

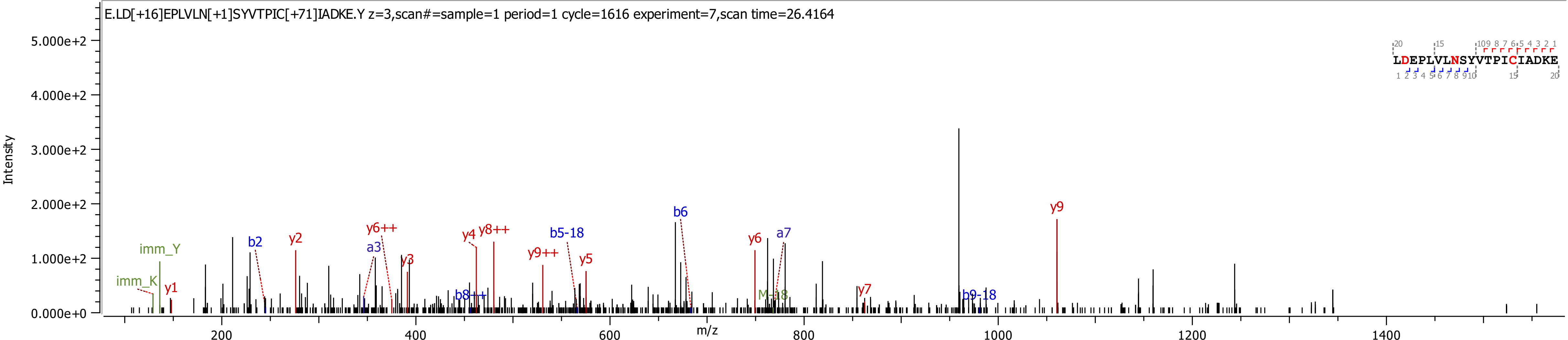

E.LD[+16]EPLVLN[+1]SYVTPIC[+71]IADKE.Y z=3,scan#=sample=1 period=1 cycle=1616 experiment=7,scan time=26.4164
Intensity
m/z
LDEPLVLNSYVTPICIADKE
imm_Y
imm_K
y1
b2
y2
y6++
a3
y3
b8++
y4
y8++
y9++
b5-18
y5
b6
y6
a7
M-18
y7
b9-18
y9

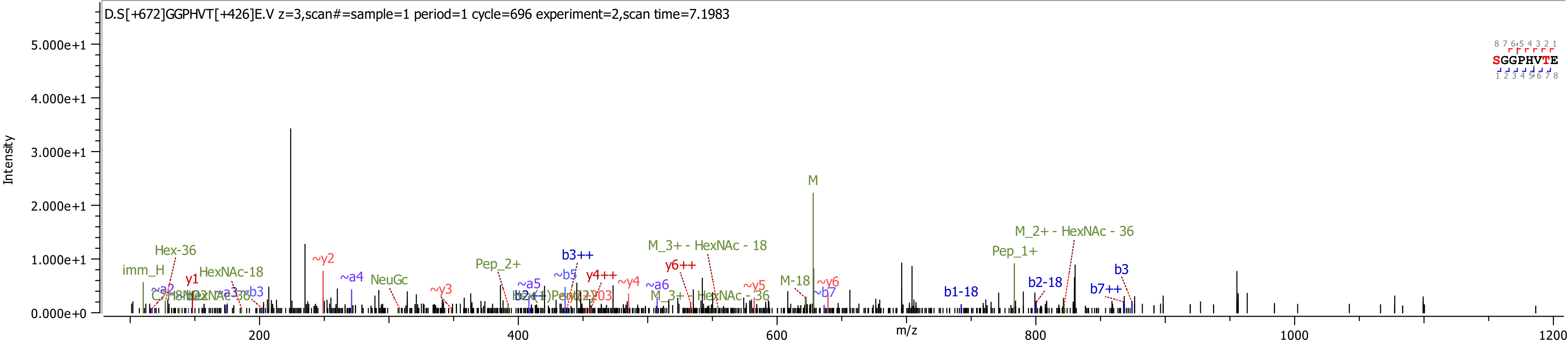
D.S[+672]GGPHVT[+426]E.V z=3,scan#=sample=1 period=1 cycle=696 experiment=2,scan time=7.1983
SGGPHVTE
Intensity
m/z
M
M_2+ - HexNAc - 36
Pep_1+
M_3+ - HexNAc - 18
b3++
b3
b7++
b2-18
b1-18
~b7
~y6
M-18
~y5
y6++
M_3+ - HexNAc - 36
~a6
~y4
y4++
~b5
~a5
Pep_2+
~y3
NeuGc
~a4
~y2
HexNAc-18
Hex-36
imm_H
y1
~a2
~a3
~b3

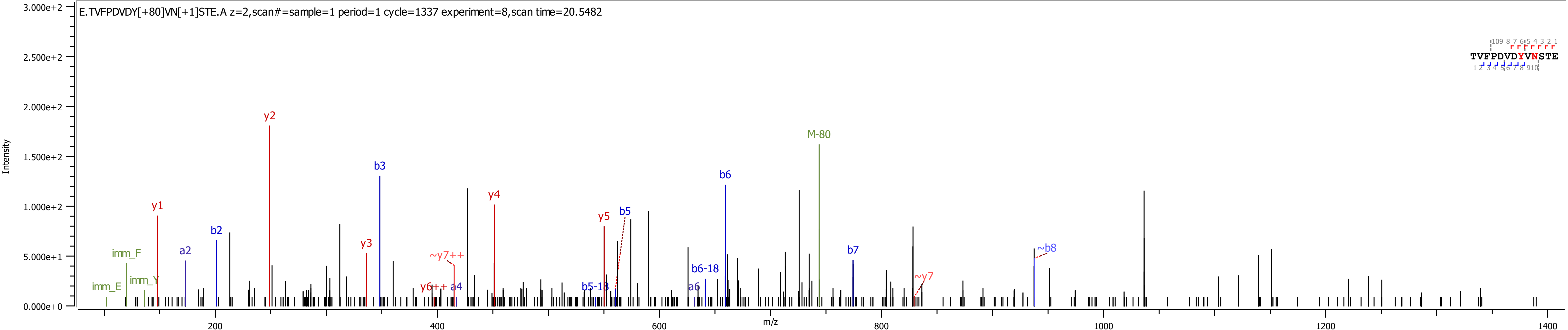

E.TVFPDVDY[+80]VN[+1]STE.A z=2,scan#=sample=1 period=1 cycle=1337 experiment=8,scan time=20.5482
Intensity
m/z
y1
y2
y3
y4
y5
b2
b3
b5
b6
b7
a2
a4
a6
M-80
imm_F
imm_E
imm_Y
~y7++
y6++
b5-18
b6-18
~y7
~b8
TVFPDVDYVNSTE

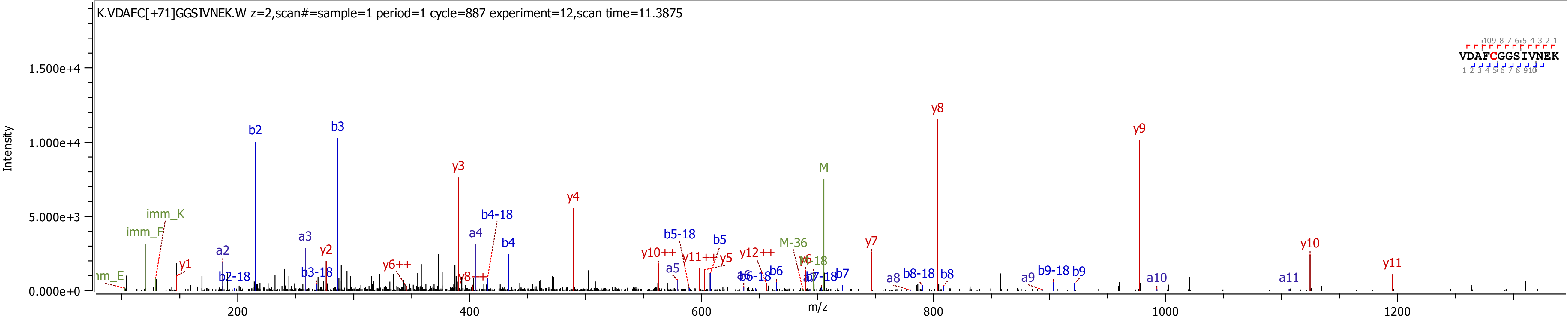
K.VDAFC[+71]GGSIVNEK.W z=2,scan#=sample=1 period=1 cycle=887 experiment=12,scan time=11.3875
VDAFCGGSIVNEK
Intensity
1.500e+4
1.000e+4
5.000e+3
0.000e+0
m/z
200
400
600
800
1000
1200
y8
y9
b2
b3
y3
M
y4
b4-18
a4
b4
imm_K
imm_F
a3
a2
y2
y1
b2-18
b3-18
y6++
y8++
b5-18
b5
y10++
y11++
y5
y12++
M-36
y7
y10
y11
a5
b6
b7
a8
b8-18
b8
a9
b9-18
b9
a10
a11

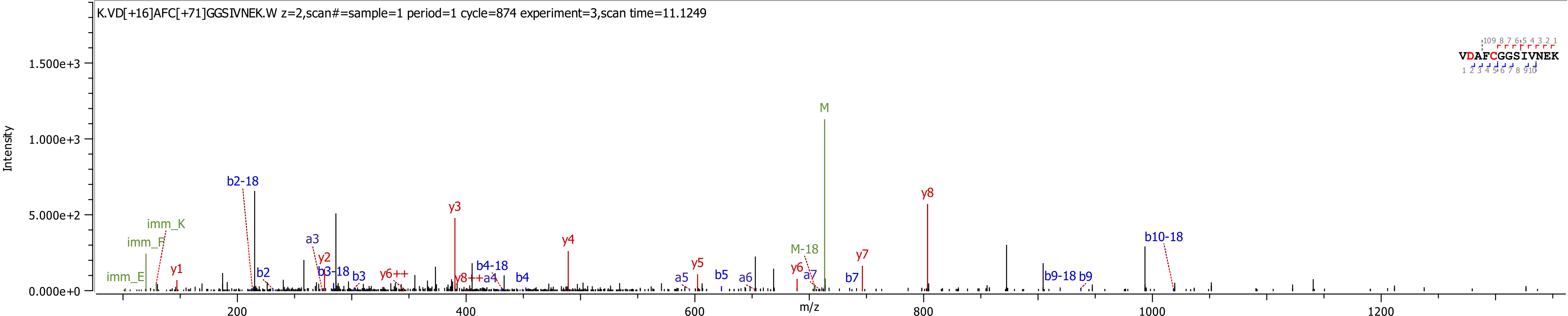

K.VD[+16]AFC[+71]GGSIVNEK.W z=2,scan#=sample=1 period=1 cycle=874 experiment=3,scan time=11.1249
Intensity
m/z
VDAFCGGSIVNEK
M
y8
y3
y4
b2-18
b10-18
M-18
y7
y6
y5
y2
y1
a3
b3-18
b3
b2
b4-18
b4
a4
y8++
y6++
a5
b5
a6
a7
b7
b9-18
b9
imm_K
imm_F
imm_E
1.500e+3
1.000e+3
5.000e+2
0.000e+0
200
400
600
800
1000
1200

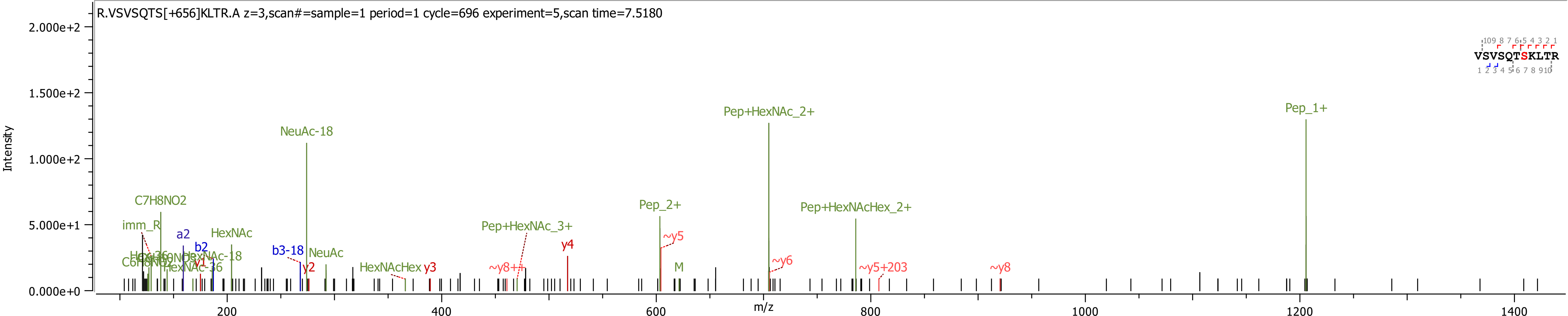
R.VSVSQTS[+656]KLTR.A z=3,scan#=sample=1 period=1 cycle=696 experiment=5,scan time=7.5180
Intensity
2.000e+2
1.500e+2
1.000e+2
5.000e+1
0.000e+0
m/z
200
400
600
800
1000
1200
1400
VSVSQTSKLTR
NeuAc-18
Pep+HexNAc_2+
Pep_1+
C7H8NO2
imm_R
a2
b2
HexNAc
HexNAc-18
HexNAc-36
b3-18
NeuAc
y1
y2
HexNAcHex
y3
~y8++
Pep+HexNAc_3+
y4
Pep_2+
~y5
M
~y6
Pep+HexNAcHex_2+
~y5+203
~y8

ProteinPilot™ Software - [Result - D:\2018\20181030_Schulz_Luci_FIXH1H2\20181106_Schulz_Luci_H1_TTP.group]

File Configure Window Help

Workflow Tasks

Identify Proteins
- LC...
- Spot-Based (MS only)...
- Spot-Based (MS and MS/MS)...

View
- Analysis Log...
- Result...

Export
- Peptide Summary...
- Distinct Peptide Summary...
- Protein Summary...
- Spectrum Summary...
- MGF Peaklist(s)...
- mzIdentML...
- Features...

Protein ID | Features | Spectra | Summary Statistics

Spectrum List

Spectrum	Acq Time	Obs MW	Obs m/z	Obs z	Prot N	Best Sequence	Modifications	Conf	Theor MW	z
2.1.1.885.2	10.282	810.4155	406.2150	2	1, 2	TTEFWK		99	810.3912	2

Peptide ID Hypotheses - 2.1.1.885.2

Conf	Sc	Prot N	Sequence	Modifications	Theor MW	Theor m/z	Obs m/z	z	ΔMass
99	9	1, 2	TTEFWK		810.3912	406.2029	406.2150	2	0.0243

Precursor MS Region

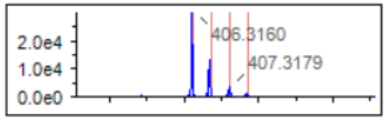

Fragmentation Evidence for Peptide

TTEFWK

Residue	b	b+2	y	y+2
T	102.0550	51.5311	811.3985	406.2029
T	203.1026	102.0550	710.3508	355.6790
E	332.1452	166.5763	609.3031	305.1552
F	479.2136	240.1105	480.2605	240.6339
W	665.2930	333.1501	333.1921	167.0997
K	793.3879	397.1976	147.1128	74.0600

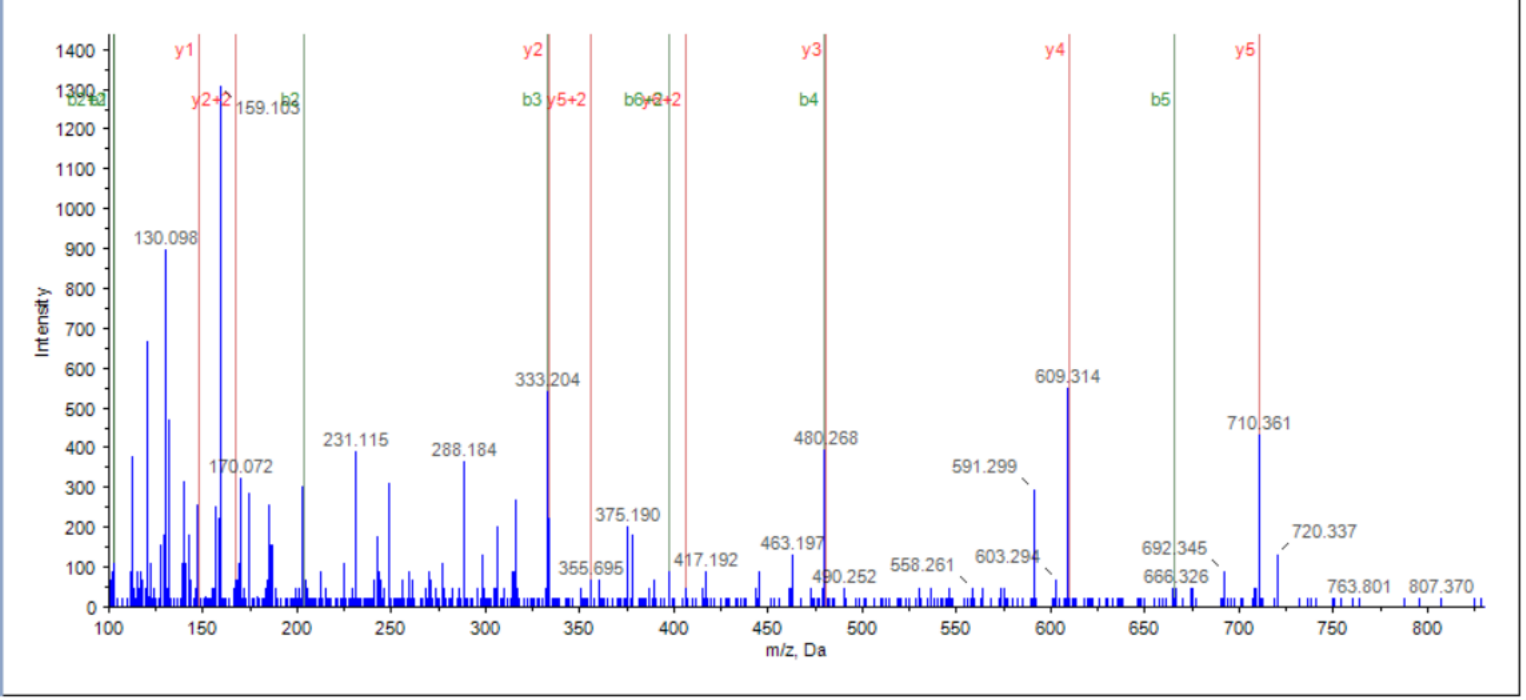

Type here to search

2:53 PM
3/04/2020

ProteinPilot™ Software - [Result - D:\2018\20181030_Schulz_Luci_FIXH1H2\20181106_Schulz_Luci_H1_TTP.group]

File Configure Window Help

Workflow Tasks

Identify Proteins
- LC...
- Spot-Based (MS only)...
- Spot-Based (MS and MS/MS)...

View
- Analysis Log...
- Result...

Export
- Peptide Summary...
- Distinct Peptide Summary...
- Protein Summary...
- Spectrum Summary...
- MGF Peaklist(s)...
- mzIdentML...
- Features...

Protein ID | Features | Spectra | Summary Statistics

Spectrum List

Spectrum	Acq Time	Obs MW	Obs m/z	Obs z	Prot N	Best Sequence	Modifications	Conf	Theor MW	z
4.1.1.949.3	12.135	854.3766	428.1956	2	1, 2	TTEFWK	Carboxy(E)@3	99	854.3810	2

Peptide ID Hypotheses - 4.1.1.949.3

Conf	Sc	Prot N	Sequence	Modifications	Theor MW	Theor m/z	Obs m/z	z	ΔMass
99	6	1, 2	TTEFWK	Carboxy(E)@3	854.3810	428.1978	428.1956	2	-0.0045

Precursor MS Region

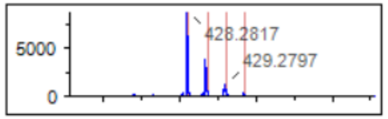

Fragmentation Evidence for Peptide

TTE[Cox]FWK

Residue	b	b+2	y	y+2
T	102.0550	51.5311	855.3883	428.1
T	203.1026	102.0550	754.3406	377.6
E[Cox]	376.1351	188.5712	653.2930	327.1
F	523.2035	262.1054	480.2605	240.6
W	709.2828	355.1450	333.1921	167.0
K	837.3777	419.1925	147.1128	74.0

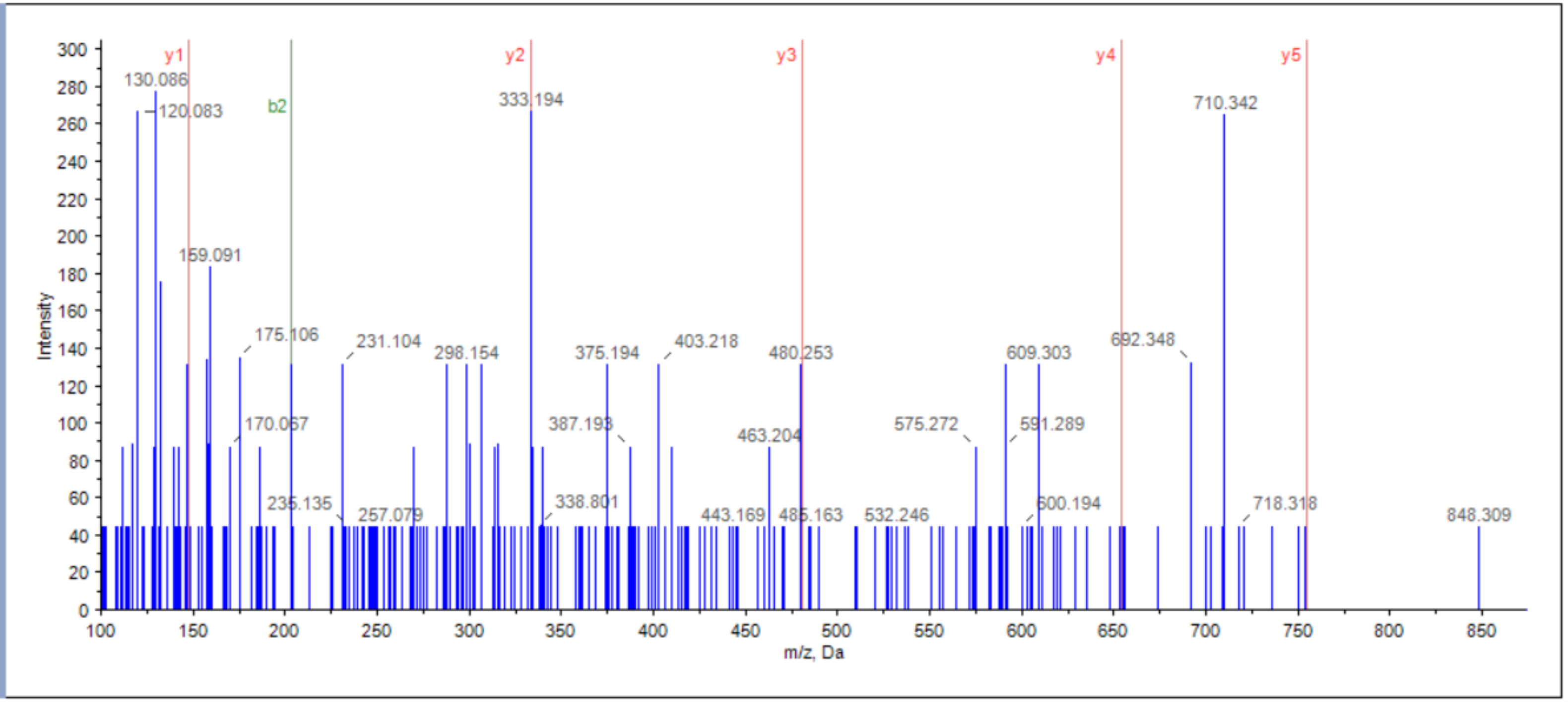

Type here to search

ENG 10:42 PM 2/04/2020

ProteinPilot™ Software - [Result - D:\2018\20181030_Schulz_Luci_FIXH1H2\20181106_Schulz_Luci_H1_GGP.group]

File Configure Window Help

Workflow Tasks

Identify Proteins
- LC...
- Spot-Based (MS only)...
- Spot-Based (MS and MS/MS)...

View
- Analysis Log...
- Result...

Export
- Peptide Summary...
- Distinct Peptide Summary...
- Protein Summary...
- Spectrum Summary...
- MGF Peaklist(s)...
- mzIdentML...
- Features...

Protein ID | Features | Spectra | Summary Statistics

Spectrum List

Spectrum	Acq Time	Obs MW	Obs m/z	Obs z	Prot N	Best Sequence	Modifications	Conf	Theor MW	z
2.1.1.1290.4	18.940	2341.9673	781.6630	3	1	TVFPDVDYVNSTEAE	Deamidated(N)@10, Hex(1)HexNAc(1)NeuAc(1)(T)@12	96.1	2341.9585	3
2.1.1.1053.7	13.913	2345.1851	782.7356	3	1	NQKSCEPAVPFPCGRVSVSQ	Propionamide@N-term, Propionamide(C)@5, Propionamide(C)@13	98.6	2345.1257	3

Peptide ID Hypotheses - 2.1.1.1290.4

Conf	Sc	Prot N	Sequence	Modifications	Theor MW	Theor m/z	Obs m/z	z	ΔMass
96.1	9	1	TVFPDVDYVNSTEAE	Deamidated(N)@10, Hex(1)HexNAc(1)NeuAc(1)(T)@12	2341.9585	781.6601	781.6630	3	0.008
85.9	9	1	TVFPDVDYVNSTEAE	Deamidated(N)@10, Hex(1)HexNAc(1)NeuAc(1)(S)@11	2341.9585	781.6601	781.6630	3	0.008

Precursor MS Region

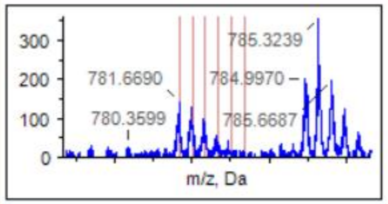

Fragmentation Evidence for Peptide

TVFPDVDYVN[Dea]ST[NHH]EAE

Residue	b	b+2	y
T	102.0550	51.5311	2342.9657
V	201.1234	101.0653	2241.9180
F	348.1918	174.5995	2142.8496
P	445.2445	223.1259	1995.7812
D	560.2715	280.6394	1898.7284
V	659.3399	330.1736	1783.7015
D	774.3668	387.6871	1684.6331
Y	937.4302	469.2187	1569.6061
V	1036.4986	518.7529	1406.5428
N[Dea]	1151.5255	576.2664	1307.4744
S	1238.5576	619.7824	1192.4475
T[NHH]	1995.8329	998.4201	1105.4154
E	2124.8754	1062.9414	348.1401
A	2195.9126	1098.4599	219.0975
E	2324.9552	1162.9812	148.0604

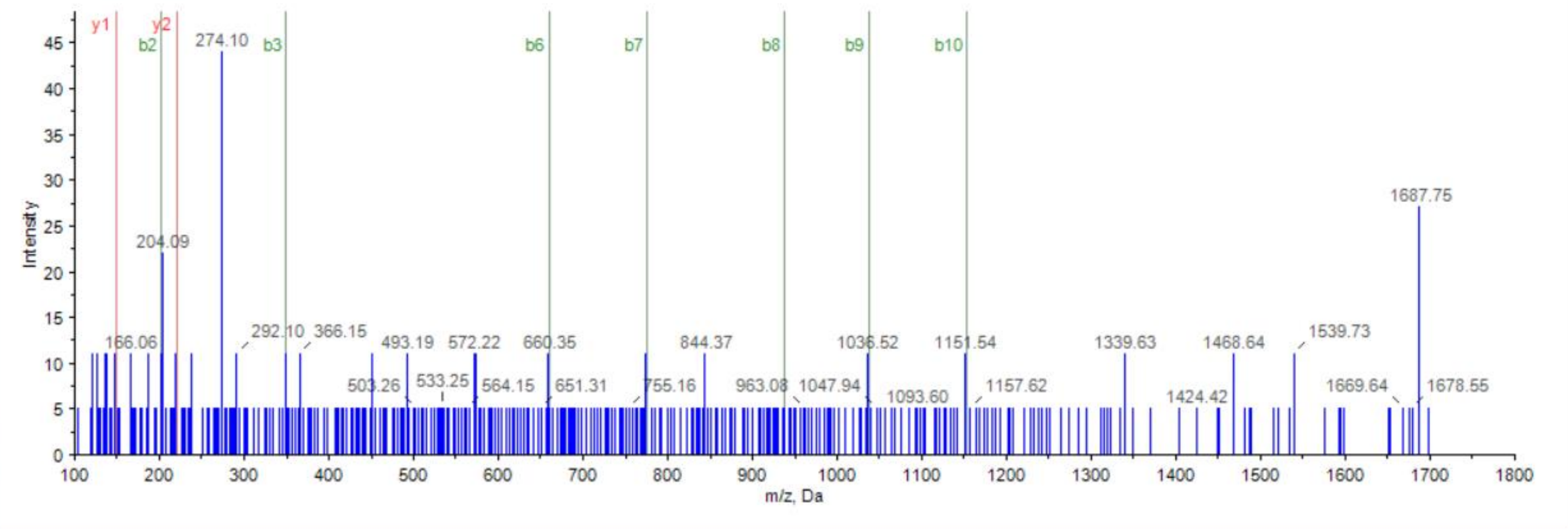

Type here to search

ENG 10:27 PM 2/04/2020
