# Supplementary material for: Coagulation factor IX analysis in bioreactor cell culture supernatant predicts quality of the purified product
Source: Commun Biol. 2021 Mar 23;4:390. doi: 10.1038/s42003-021-01903-x (PMC7988164; doi:10.1038/s42003-021-01903-x)
Supplement: Supplementary file 10 — Supplementary Data S7 [file 42003_2021_1903_MOESM10_ESM.pdf]

20190702_Co4Ra2NeuGcOglyLacNAc_20190509_BenSchulz_Luci_H2a_Ch.wiff_Byonic_D359_D364 1
20190702_Co4Ra2NeuGcOglyLacNAc_20190509_BenSchulz_Luci_H2a_Ch.wiff_Byonic_E15_E17_E20_E21 2
20190702_Co4Ra2NeuGcOglyLacNAc_20190509_BenSchulz_Luci_H2a_Ch.wiff_Byonic_E20_E21_E26_E27_E30[+44] 3
20190702_Co4Ra2NeuGcOglyLacNAc_20190509_BenSchulz_Luci_H2a_Ch.wiff_Byonic_E20_E21_E30_E33_E36 4
20190702_Co4Ra2NeuGcOglyLacNAc_20190509_BenSchulz_Luci_H2a_Ch.wiff_Byonic_E33_E36_E40 5

20190702_Co4Ra2NeuGcOglyLacNAc_20190509_BenSchulz_Luci_H2a_Ch.wiff_Byonic_E33_E36_E40[+44] 6
20190702_Co4Ra2NeuGcOglyLacNAc_20190509_BenSchulz_Luci_H2a_Ch.wiff_Byonic_E33_E36_E40[+44]x2 7
20190702_Co4Ra2NeuGcOglyLacNAc_20190509_BenSchulz_Luci_H2a_Ch.wiff_Byonic_S53[+426] 8
20190702_Co4Ra2NeuGcOglyLacNAc_20190509_BenSchulz_Luci_H2a_Ch.wiff_Byonic_T179[+656] 9
20190702_Co4Ra2NeuGcOglyLacNAc_20190509_BenSchulz_Luci_H2a_Ch.wiff_Byonic_T179[+947] 10

Common4rare2NeuGc50_20181106_Schulz_Luci_H2aG.wiff_20181116_Byonic_D47[+16] 11
Common4rare2NeuGc50_20181106_Schulz_Luci_H2aG.wiff_20181116_Byonic_D49[+16] 12
Common4rare2NeuGc50_20181106_Schulz_Luci_H2aG.wiff_20181116_Byonic_D104 13
Common4rare2NeuGc50_20181106_Schulz_Luci_H2aG.wiff_20181116_Byonic_D104[+16] 14
Common4rare2NeuGc50_20181106_Schulz_Luci_H2aG.wiff_20181116_Byonic_D276_D292 15

Common4rare2NeuGc50_20181106_Schulz_Luci_H2aG.wiff_20181116_Byonic_D292[+16] 16
Common4rare2NeuGc50_20181106_Schulz_Luci_H2aG.wiff_20181116_Byonic_N157[+1] 17
Common4rare2NeuGc50_20181106_Schulz_Luci_H2aG.wiff_20181116_Byonic_N258 18
Common4rare2NeuGc50_20181106_Schulz_Luci_H2aG.wiff_20181116_Byonic_N258[+1] 19
Common4rare2NeuGc50_20181106_Schulz_Luci_H2aG.wiff_20181116_Byonic_S53[+426]_S61[+802]_D64[+16]_D65[+16]_S68[+ 20

Common4rare2NeuGc50_20181106_Schulz_Luci_H2aG.wiff_20181116_Byonic_S110[+426]_T112[+802] 21
Common4rare2NeuGc50_20181106_Schulz_Luci_H2aG.wiff_20181116_Byonic_Y45_D47_D49 22
Common4rare2NeuGc50_20181106_Schulz_Luci_H2aG.wiff_20181116_Byonic_Y45[+80] 23
Common4rare2NeuGc50_20181106_Schulz_Luci_H2aGP.wiff_20181116_Byonic_N167[+1] 24
Common4rare2NeuGc50_20181106_Schulz_Luci_H2aGP.wiff_20181116_Byonic_S158[+947] 25

Common4rare2NeuGc50_20181106_Schulz_Luci_H2aT.wiff_20181119_Byonic_D64 26
Common4rare2NeuGc50_20181106_Schulz_Luci_H2aT.wiff_20181119_Byonic_D64[+16] 27
Common4rare2NeuGc50_20181106_Schulz_Luci_H2aT.wiff_20181119_Byonic_D85 28
Common4rare2NeuGc50_20181106_Schulz_Luci_H2aT.wiff_20181119_Byonic_D85[+16] 29
Common4rare2NeuGc50_20181106_Schulz_Luci_H2aT.wiff_20181119_Byonic_D203 30

Common4rare2NeuGc50_20181106_Schulz_Luci_H2aT.wiff_20181119_Byonic_D203[+16] 31
Common4rare2NeuGc50_20181106_Schulz_Luci_H2aT.wiff_20181119_Byonic_E7_E9_E15 32
Common4rare2NeuGc50_20181106_Schulz_Luci_H2aT.wiff_20181119_Byonic_E7_E9_E15[+44] 33
Common4rare2NeuGc50_20181106_Schulz_Luci_H2aT.wiff_20181119_Byonic_E7_E9_E15[+44]x2 34
Common4rare2NeuGc50_20181106_Schulz_Luci_H2aT.wiff_20181119_Byonic_E7_E9_E15[+44]x3 35

Common4rare2NeuGc50_20181106_Schulz_Luci_H2aT.wiff_20181119_Byonic_S141[+656] 36
Common4rare2NeuGc50_20181106_Schulz_Luci_H2aT.wiff_20181119_Byonic_S141[+947] 37
Common4rare2NeuGc50_20181106_Schulz_Luci_H2aT.wiff_20181119_Byonic_T38[+656] 38
Common4rare2NeuGc50_20181106_Schulz_Luci_H2aT.wiff_20181119_Byonic_T38[+947] 39
Common4rare2NeuGc50_20181106_Schulz_Luci_H2aT.wiff_20181119_Byonic_T38[+963] 40

Common4rare2NeuGc50_20181106_Schulz_Luci_H2bG.wiff_20181116_Byonic_Y155[+80] 41
Common4rare2NeuGc50_20181106_Schulz_Luci_H2bGP.wiff_20181116_Byonic_D276{+16] 42
Common4rare2NeuGc50_20181106_Schulz_Luci_H2bGP.wiff_20181116_Byonic_T169orT172[+947] 43
Common4rare2NeuGc50_20181106_Schulz_Luci_H2bT.wiff_20181119_Byonic_E26_E27_E30_E33_E36[+44]x3 44
Common4rare2NeuGc50_20181106_Schulz_Luci_H2bTP.wiff_20181119_Byonic_S53[+426]_S61 45

ProteinPilot_E40[+44] 46

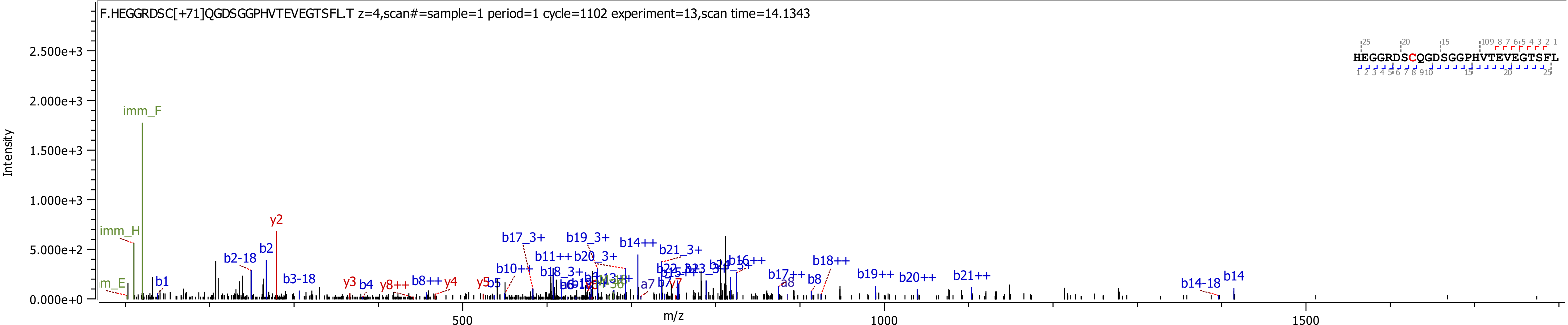
F.HEGGRDSC[+71]QGDSGGPHVTEVEGTSFL.T z=4,scan#=sample=1 period=1 cycle=1102 experiment=13,scan time=14.1343
Intensity
m/z
0.000e+0
5.000e+2
1.000e+3
1.500e+3
2.000e+3
2.500e+3
500
1000
1500
HEGGRDSCQGDSGGPHVTEVEGTSFL
imm_F
imm_H
y2
b2
b2-18
b1
b3-18
y3
b4
y8++
b8++
y4
y5
b5
b10++
b17_3+
b11++
b19_3+
b20_3+
b14++
b21_3+
b16++
b18++
b17++
b8
b19++
b20++
b21++
b14-18
b14
a7
a8

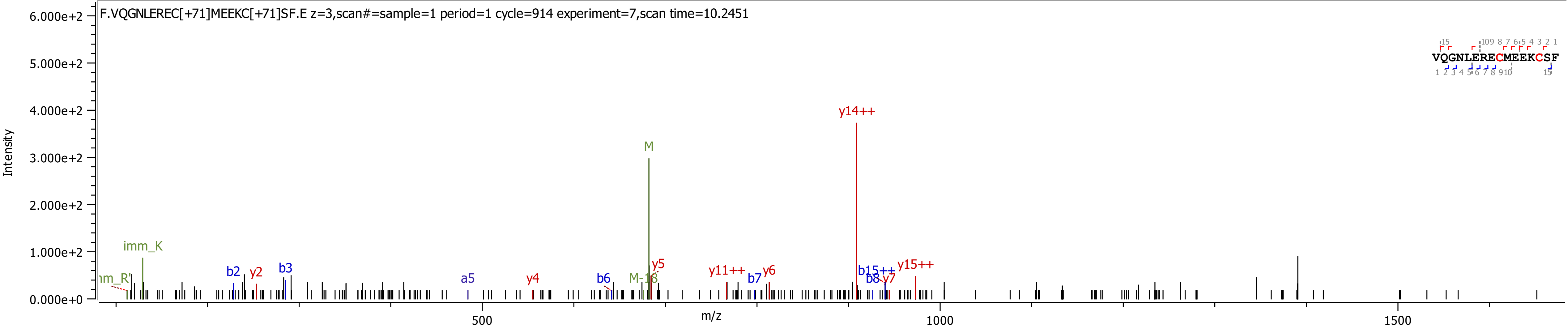

F.VQGNLEREC[+71]MEEKC[+71]SF.E z=3,scan#=sample=1 period=1 cycle=914 experiment=7,scan time=10.2451
Intensity
m/z
VQGNLERECMEEKCSF
y14++
M
imm_K
b2
y2
b3
a5
y4
b6
M-18
y5
y11++
b7
y6
b15++
b8
y7
y15++

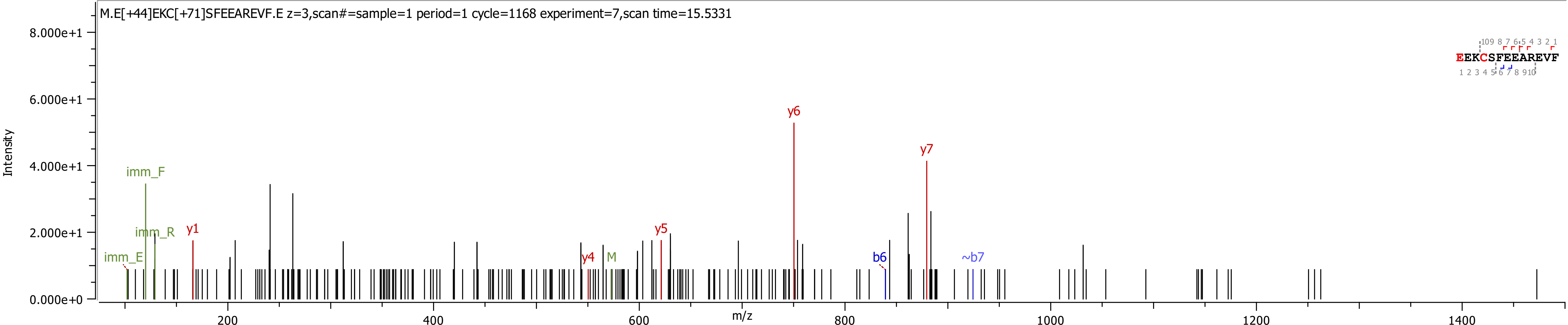

M.E[+44]EKC[+71]SFEEAREVF.E z=3,scan#=sample=1 period=1 cycle=1168 experiment=7,scan time=15.5331
8.000e+1
6.000e+1
4.000e+1
2.000e+1
0.000e+0
Intensity
m/z
200
400
600
800
1000
1200
1400
imm_F
imm_R
imm_E
y1
y4
M
y5
y6
y7
b6
~b7
EEKCSFEEAREVF

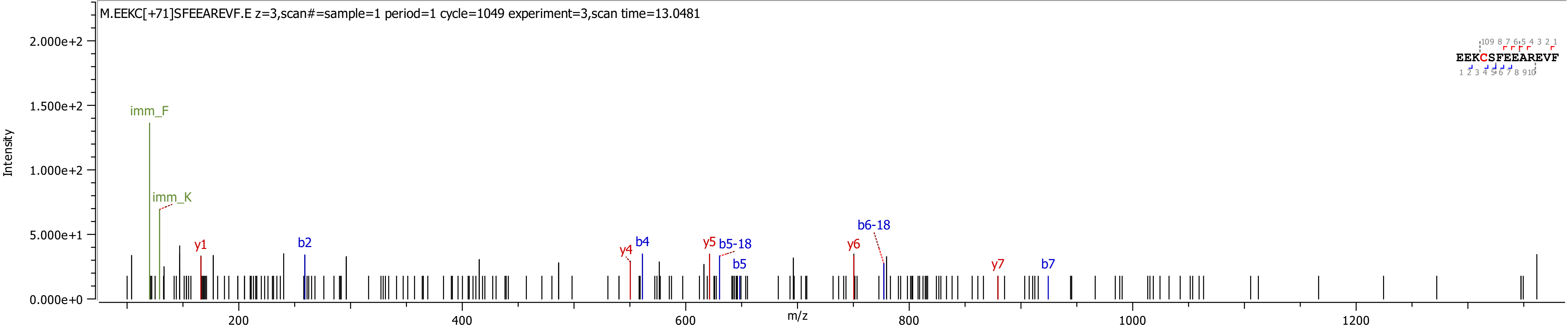
M.EEKC[+71]SFEEAREVF.E z=3,scan#=sample=1 period=1 cycle=1049 experiment=3,scan time=13.0481
Intensity
2.000e+2
1.500e+2
1.000e+2
5.000e+1
0.000e+0
m/z
200
400
600
800
1000
1200
imm_F
imm_K
y1
b2
y4
b4
y5
b5-18
b5
b6-18
y6
y7
b7
EEKCSFEEAREVF

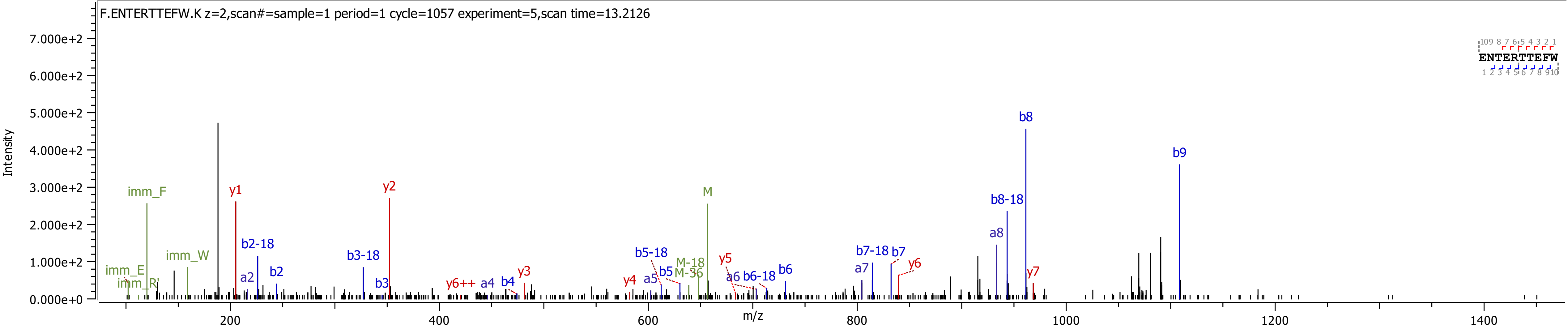

F.ENTERTTEFW.K z=2,scan#=sample=1 period=1 cycle=1057 experiment=5,scan time=13.2126
Intensity
m/z
7.000e+2
6.000e+2
5.000e+2
4.000e+2
3.000e+2
2.000e+2
1.000e+2
0.000e+0
200
400
600
800
1000
1200
1400
imm_F
imm_W
imm_E
imm_R
y1
y2
y3
y4
y5
y6
y7
y6++
a2
a4
a5
a6
a7
a8
b2
b2-18
b3
b3-18
b4
b5
b5-18
b6
b6-18
b7
b7-18
b8
b8-18
b9
M
M-18
M-36
ENTERTTEFW

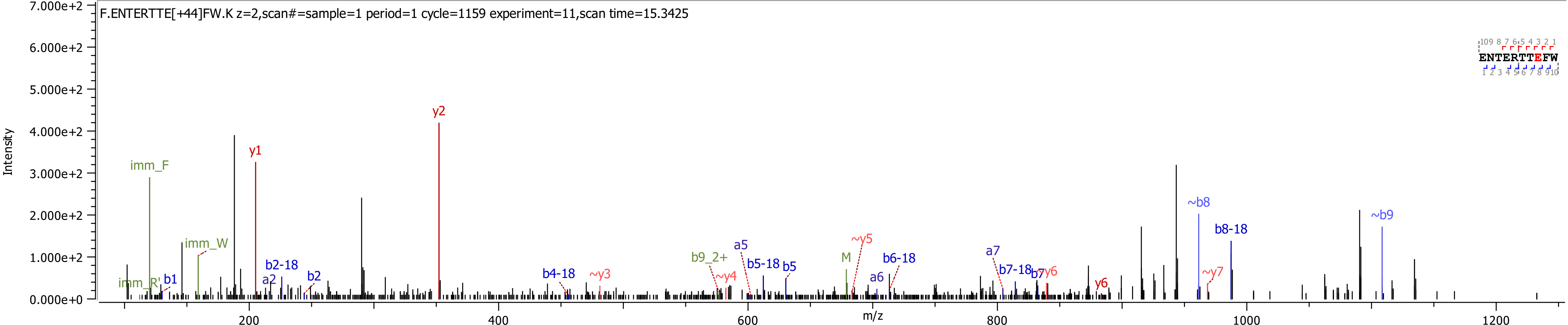

F.ENTERTTE[+44]FW.K z=2,scan#=sample=1 period=1 cycle=1159 experiment=11,scan time=15.3425
ENTERTTEFW
Intensity
m/z

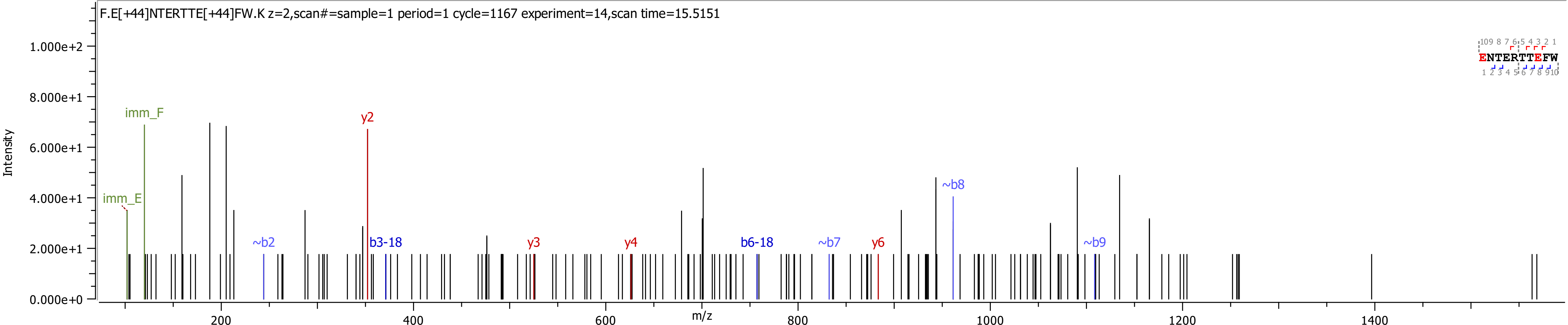
F.E[+44]NTERTTE[+44]FW.K z=2,scan#=sample=1 period=1 cycle=1167 experiment=14,scan time=15.5151
Intensity
m/z
imm_F
imm_E
y2
~b2
b3-18
y3
y4
b6-18
~b7
y6
~b8
~b9
ENTERTTEFW
0.000e+0
2.000e+1
4.000e+1
6.000e+1
8.000e+1
1.000e+2
200
400
600
800
1000
1200
1400

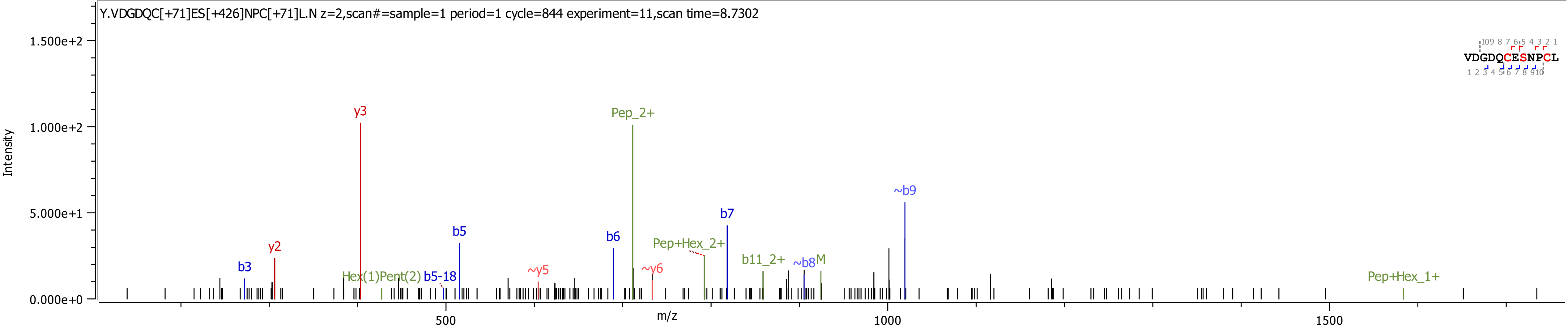

Y.VDGDQC[+71]ES[+426]NPC[+71]L.N z=2,scan#=sample=1 period=1 cycle=844 experiment=11,scan time=8.7302
Intensity
1.500e+2
1.000e+2
5.000e+1
0.000e+0
m/z
500
1000
1500
y3
Pep_2+
~b9
b7
b5
b6
Pep+Hex_2+
y2
b3
~y5
~y6
b11_2+
~b8
M
Hex(1)Pent(2)
b5-18
Pep+Hex_1+
VDGDQCESNPCL

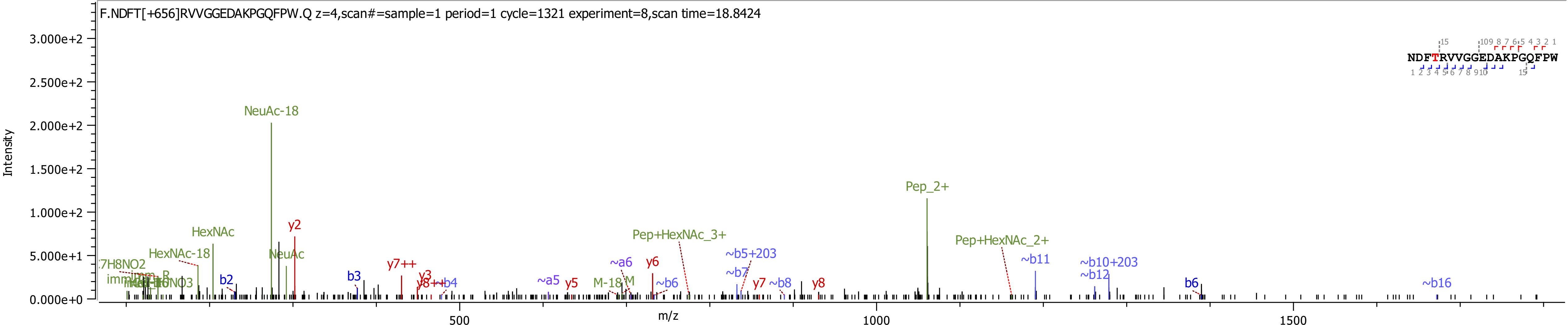
F.NDFT[+656]RVVGGEDAKPGQFPW.Q z=4,scan#=sample=1 period=1 cycle=1321 experiment=8,scan time=18.8424
NDFTRVVGGEDAKPGQFPW
Intensity
m/z
NeuAc-18
Pep_2+
HexNAc
HexNAc-18
NeuAc
y2
Pep+HexNAc_3+
Pep+HexNAc_2+
~b5+203
~b10+203
~b11
~b12
~b7
~a6
y6
~b6
y7++
y3
y8++
~b4
b2
b3
~a5
y5
M-18
M
y7
~b8
y8
b6
~b16
500
1000
1500

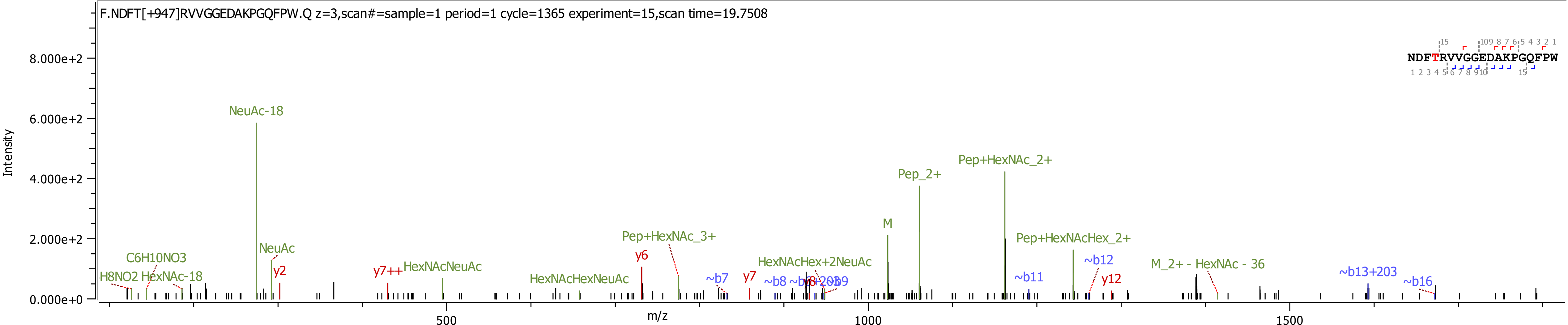
F.NDFT[+947]RVVGGEDAKPGQFPW.Q z=3,scan#=sample=1 period=1 cycle=1365 experiment=15,scan time=19.7508
Intensity
m/z
NeuAc-18
NeuAc
C6H10NO3
H8NO2
HexNAc-18
y2
y7++
HexNAcNeuAc
HexNAcHexNeuAc
Pep+HexNAc_3+
y6
~b7
y7
HexNAcHex+2NeuAc
~b8
M
Pep_2+
Pep+HexNAc_2+
Pep+HexNAcHex_2+
~b11
~b12
y12
M_2+ - HexNAc - 36
~b13+203
~b16
NDFTRVVGGEDAKPGQFPW

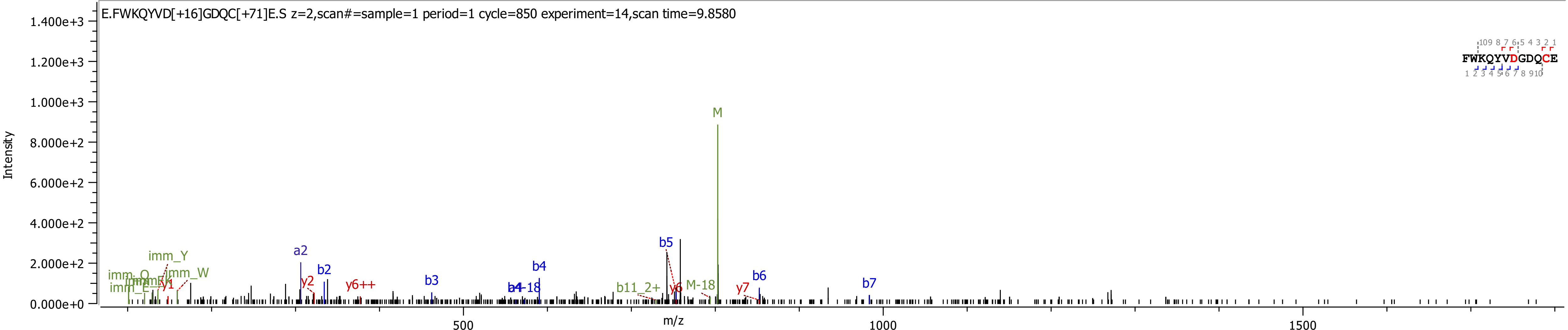
E.FWKQYVD[+16]GDQC[+71]E.S z=2,scan#=sample=1 period=1 cycle=850 experiment=14,scan time=9.8580
Intensity
1.400e+3
1.200e+3
1.000e+3
8.000e+2
6.000e+2
4.000e+2
2.000e+2
0.000e+0
m/z
500
1000
1500
M
b5
a2
b2
b4
b3
b6
b7
y2
y6++
y7
y1
imm_Y
imm_W
imm_Q
b11_2+
M-18
b4-18
FWKQYVDGDQCE

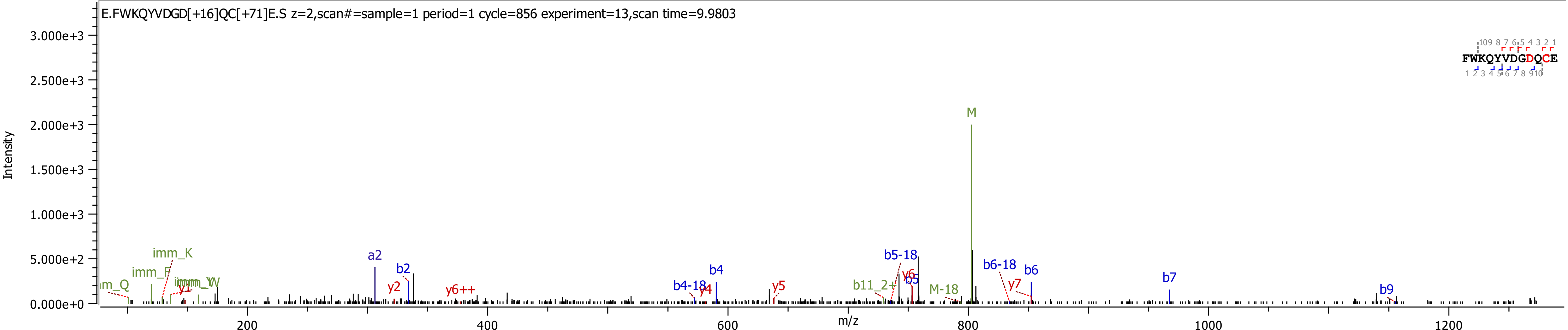

E.FWKQYVDGD[+16]QC[+71]E.S z=2,scan#=sample=1 period=1 cycle=856 experiment=13,scan time=9.9803
FWKQYVDGDQCE
Intensity
m/z
3.000e+3
2.500e+3
2.000e+3
1.500e+3
1.000e+3
5.000e+2
0.000e+0
200
400
600
800
1000
1200
M
imm_K
imm_F
a2
b2
y2
y6++
b4
b4-18
y4
y5
b5-18
b11_2+
y6
b5
M-18
b6-18
b6
y7
b7
b9

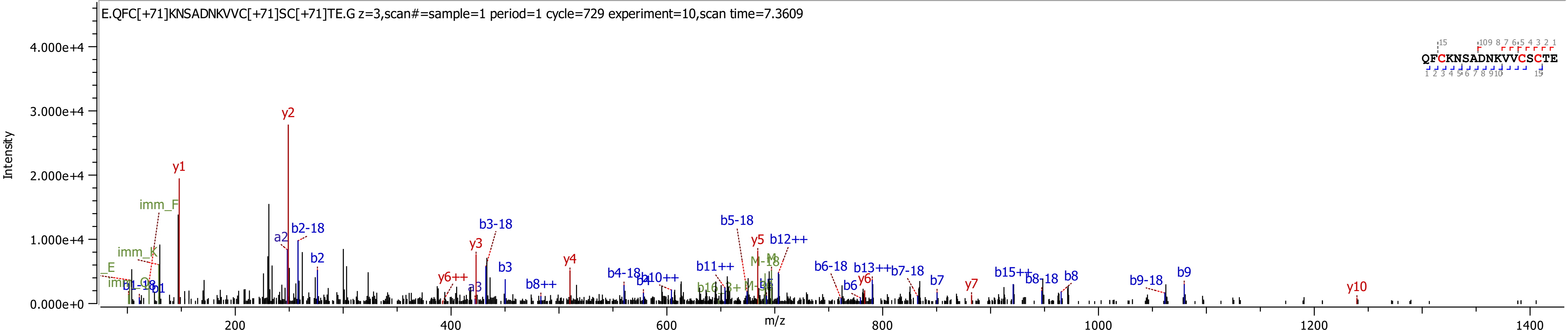
E.QFC[+71]KNSADNKVVC[+71]SC[+71]TE.G z=3,scan#=sample=1 period=1 cycle=729 experiment=10,scan time=7.3609
Intensity
4.000e+4
3.000e+4
2.000e+4
1.000e+4
0.000e+0
200
400
600
800
1000
1200
1400
m/z
QFCKNSADNKVVCSCTE
y1
y2
y3
y4
y5
y6
y7
y10
y6++
a2
a3
b1
b2
b3
b4
b6
b7
b8
b9
b2-18
b3-18
b4-18
b5-18
b6-18
b7-18
b8-18
b9-18
b8++
b10++
b11++
b12++
b13++
b15++
imm_F
imm_K
imm_Q
_E
M-18

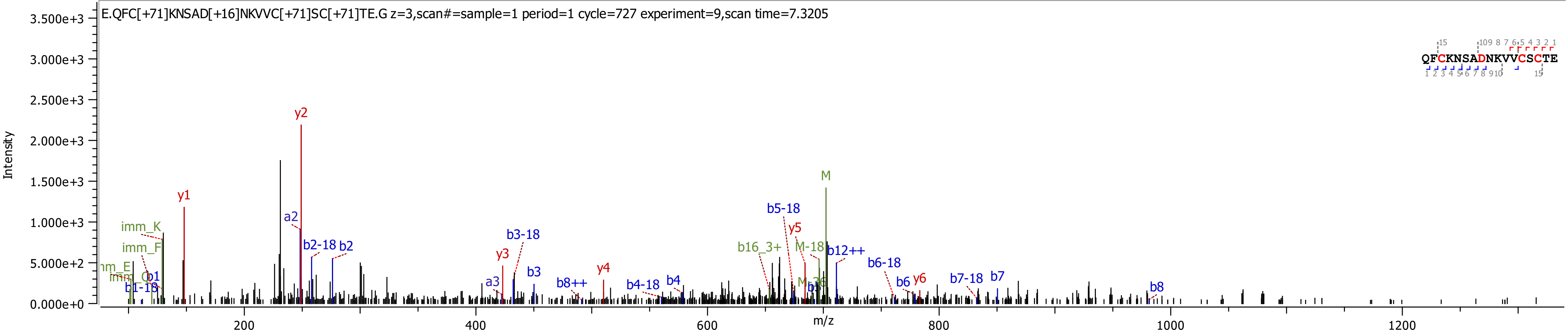

E.QFC[+71]KNSAD[+16]NKVVC[+71]SC[+71]TE.G z=3,scan#=sample=1 period=1 cycle=727 experiment=9,scan time=7.3205
QFCKNSADNKVVCSCTE
Intensity
m/z
y1
y2
y3
y4
y5
y6
a2
a3
b2-18
b2
b3-18
b3
b4-18
b4
b5-18
b6-18
b6
b7-18
b7
b8
b8++
b12++
b16_3+
M
M-18
imm_K
imm_F
b1-18
b1

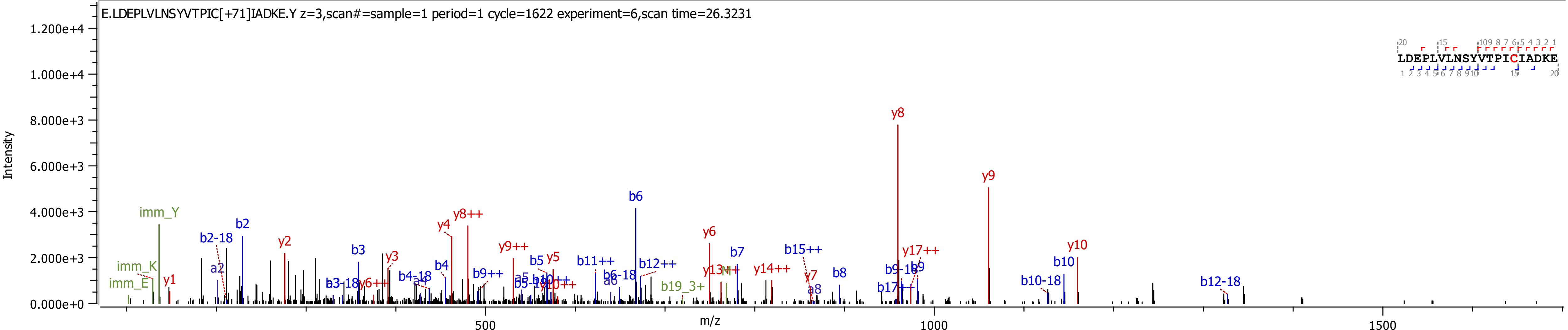
E.LDEPLVLNSYVTPIC[+71]IADKE.Y z=3,scan#=sample=1 period=1 cycle=1622 experiment=6,scan time=26.3231
LDEPLVLNSYVTPICIADKE
Intensity
m/z
0.000e+0
2.000e+3
4.000e+3
6.000e+3
8.000e+3
1.000e+4
1.200e+4
500
1000
1500
y8
y9
b6
imm_Y
b2
b2-18
y2
y8++
y4
y6
y10
b10
b12-18
b10-18
y17++
b15++
b11++
b12++
y14++
b7
b8
y9++
b3
y3
b4
a2
imm_K
imm_E
y1
b19_3+
y13++
b9++
b5
y5
a8
y7

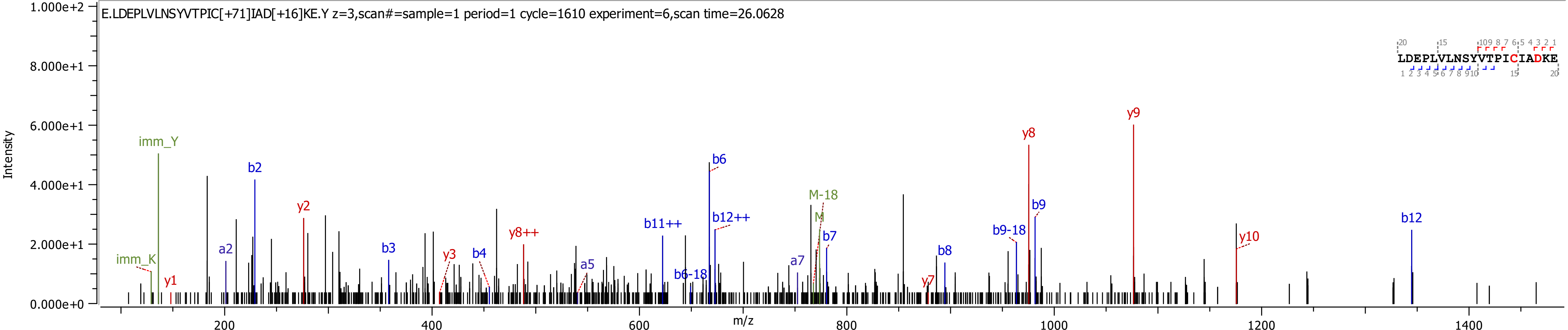
E.LDEPLVLNSYVTPIC[+71]IAD[+16]KE.Y z=3,scan#=sample=1 period=1 cycle=1610 experiment=6,scan time=26.0628
Intensity
m/z
LDEPLVLNSYVTPICIADKE
imm_Y
imm_K
y1
a2
b2
y2
b3
y3
b4
y8++
a5
b11++
b6-18
b6
b12++
a7
M-18
M
b7
y7
b8
b9-18
y8
b9
y9
y10
b12

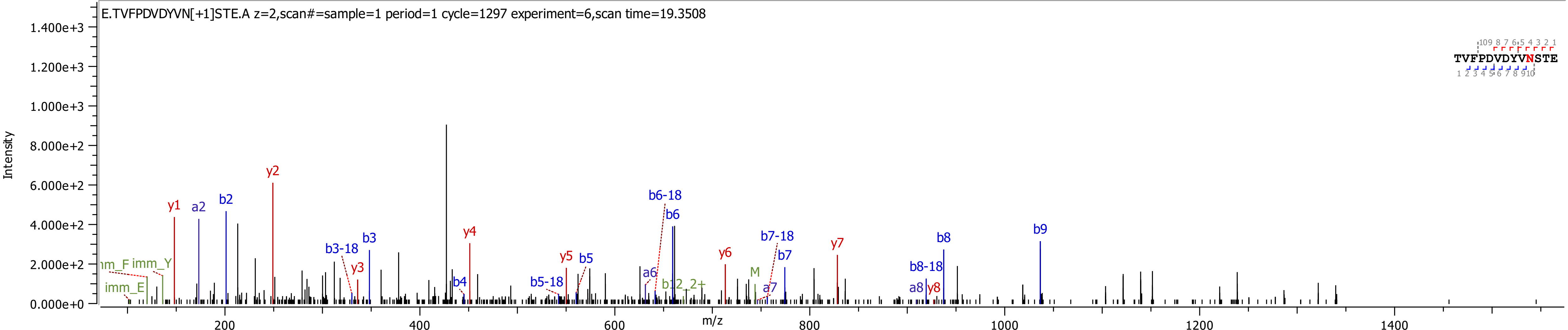
E.TVFPDVDYVN[+1]STE.A z=2,scan#=sample=1 period=1 cycle=1297 experiment=6,scan time=19.3508
TVFPDVDYVNSTE
Intensity
1.400e+3
1.200e+3
1.000e+3
8.000e+2
6.000e+2
4.000e+2
2.000e+2
0.000e+0
m/z
200
400
600
800
1000
1200
1400
y1
a2
b2
y2
b3-18
b3
y3
y4
b4
y5
b5
b5-18
b6-18
b6
a6
y6
M
b7-18
b7
a7
y7
b8-18
b8
a8
y8
b9
imm_F
imm_Y
imm_E
b12_2+

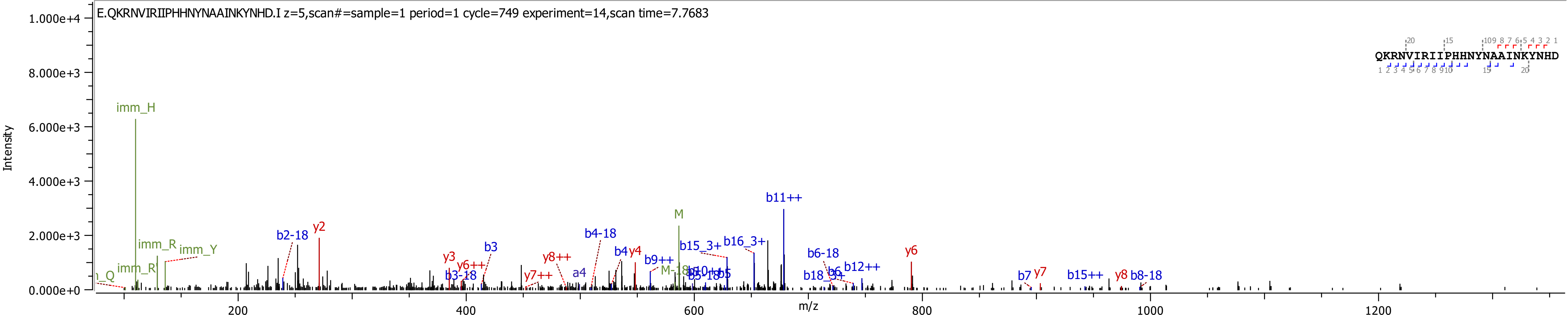
E.QKRNVIRIIPHHNYNAAINKYNHD.I z=5,scan#=sample=1 period=1 cycle=749 experiment=14,scan time=7.7683
QKRNVIRIIPHHNYNAAINKYNHD
Intensity
m/z
imm_H
imm_R
imm_Y
b2-18
y2
b3
y3
y6++
b3-18
y8++
y7++
a4
b4-18
b4
y4
b9++
M
b15_3+
b16_3+
b11++
b6-18
b12++
y6
b7
y7
b15++
y8
b8-18

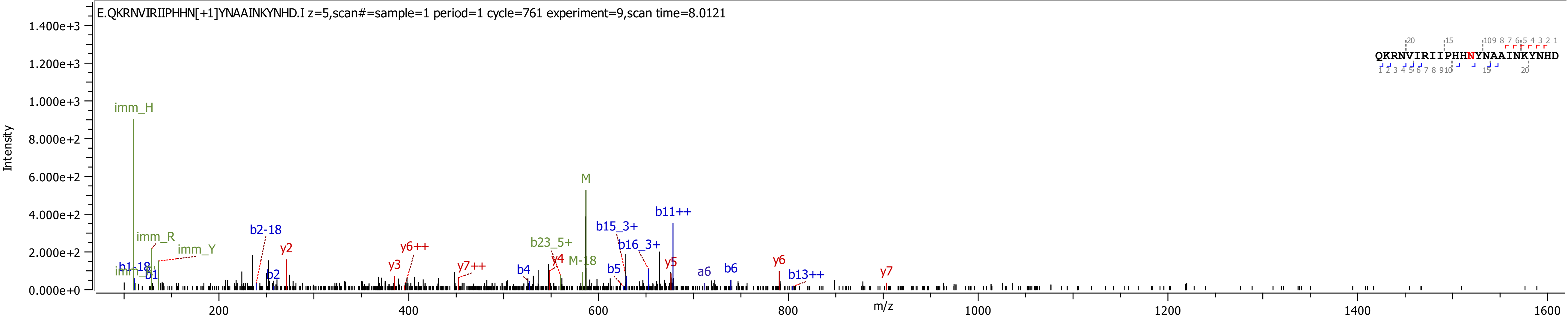
E.QKRNVIRIIPHHN[+1]YNAAINKYNHD.I z=5,scan#=sample=1 period=1 cycle=761 experiment=9,scan time=8.0121
QKRNVIRIIPHHNYNAAINKYNHD
Intensity
m/z
imm_H
M
b11++
b15_3+
b16_3+
b2-18
imm_R
imm_Y
y2
y6++
b23_5+
y4
M-18
y7++
y3
y5
y6
b13++
y7
b1-18
imm_
b1
b2
b4
b5
a6
b6
0.000e+0
2.000e+2
4.000e+2
6.000e+2
8.000e+2
1.000e+3
1.200e+3
1.400e+3
200
400
600
800
1000
1200
1400
1600

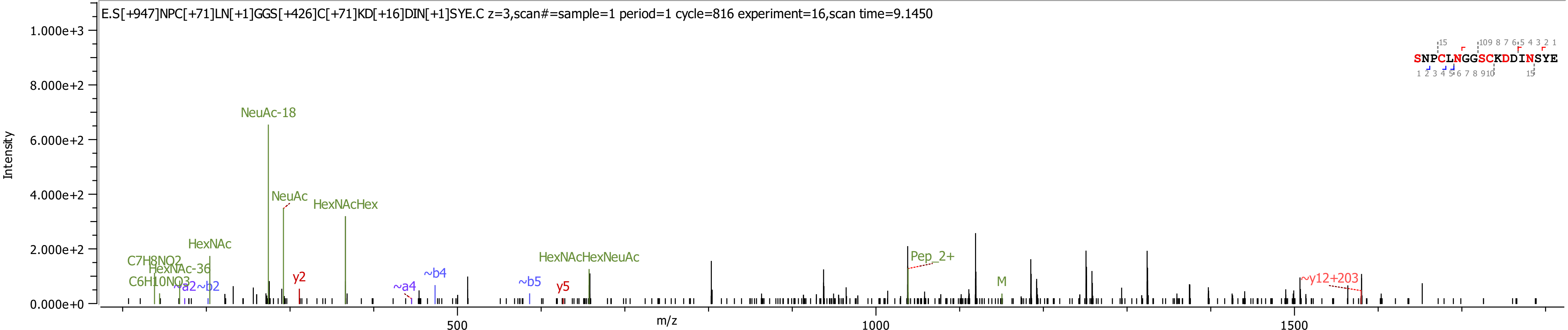
E.S[+947]NPC[+71]LN[+1]GGS[+426]C[+71]KD[+16]DIN[+1]SYE.C z=3,scan#=sample=1 period=1 cycle=816 experiment=16,scan time=9.1450
SNPCLNGGSCKDDINSYE
Intensity
1.000e+3
8.000e+2
6.000e+2
4.000e+2
2.000e+2
0.000e+0
m/z
500
1000
1500
NeuAc-18
NeuAc
HexNAcHex
HexNAc
C7H8NO2
HexNAc-36
C6H10NO3
~a2
~b2
y2
~a4
~b4
~b5
y5
HexNAcHexNeuAc
Pep_2+
M
~y12+203

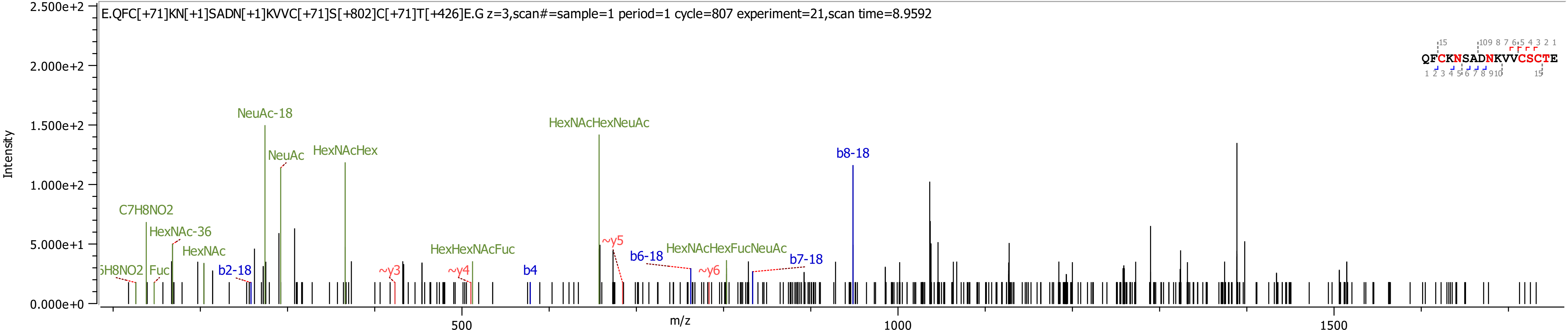

E.QFC[+71]KN[+1]SADN[+1]KVVC[+71]S[+802]C[+71]T[+426]E.G z=3,scan#=sample=1 period=1 cycle=807 experiment=21,scan time=8.9592
QFCKNSADNKVVCSCTE
Intensity
m/z
NeuAc-18
HexNAcHexNeuAc
NeuAc
HexNAcHex
b8-18
C7H8NO2
HexNAc-36
HexNAc
Fuc
b2-18
~y3
~y4
HexHexNAcFuc
b4
~y5
b6-18
HexNAcHexFucNeuAc
~y6
b7-18

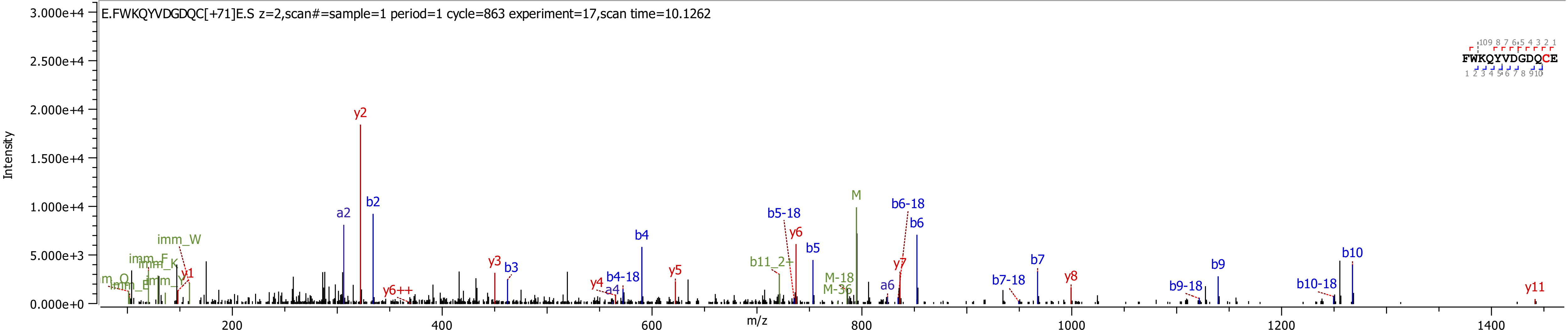

E.FWKQYVDGDQC[+71]E.S z=2,scan#=sample=1 period=1 cycle=863 experiment=17,scan time=10.1262
FWKQYVDGDQCE
Intensity
m/z
y2
a2
b2
M
b5-18
b6-18
y6
b6
b5
b4
imm_W
y3
b3
y5
b4-18
y4
a4
b11_2+
M-18
M-36
y7
a6
b7
b7-18
y8
b9
b9-18
b10
b10-18
y11
y1
y6++

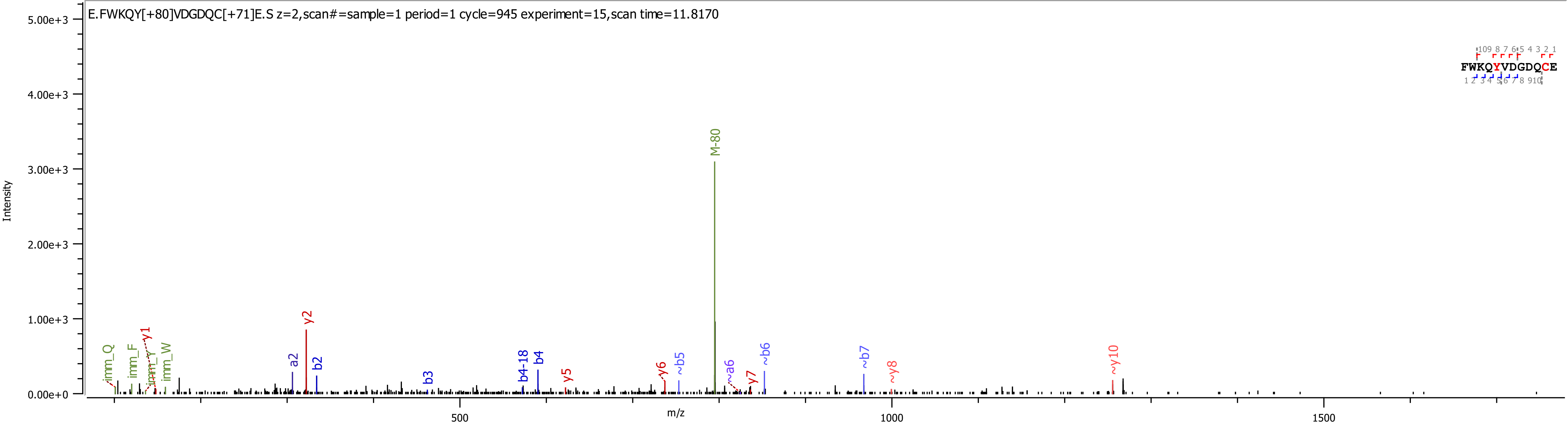
E.FWKQY[+80]VDGDQC[+71]E.S z=2,scan#=sample=1 period=1 cycle=945 experiment=15,scan time=11.8170
Intensity
5.00e+3
4.00e+3
3.00e+3
2.00e+3
1.00e+3
0.00e+0
m/z
500
1000
1500
M-80
imm_Q
imm_F
imm_Y
imm_W
y1
a2
y2
b2
b3
b4-18
b4
y5
y6
~b5
~a6
y7
~b6
~b7
~y8
~y10
FWKQYVDGDQCE

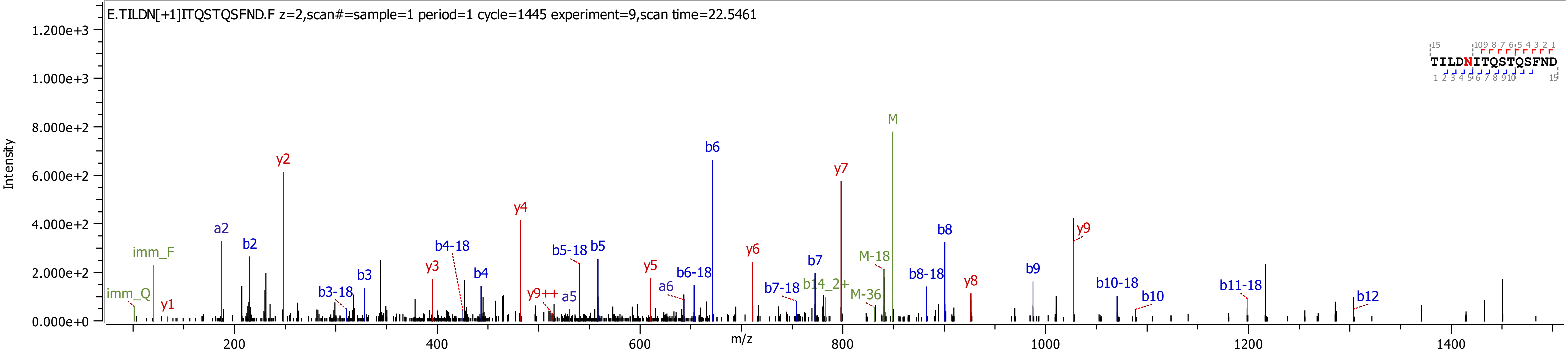
E.TILDN[+1]ITQSTQSFND.F z=2,scan#=sample=1 period=1 cycle=1445 experiment=9,scan time=22.5461
TILDNITQSTQSFND
Intensity
m/z
imm_Q
imm_F
y1
a2
b2
y2
b3-18
b3
y3
b4-18
b4
y4
y9++
a5
b5-18
b5
y5
a6
b6-18
b6
y6
b7-18
b7
b14_2+
y7
M-18
M-36
M
b8-18
b8
y8
b9
y9
b10-18
b10
b11-18
b12

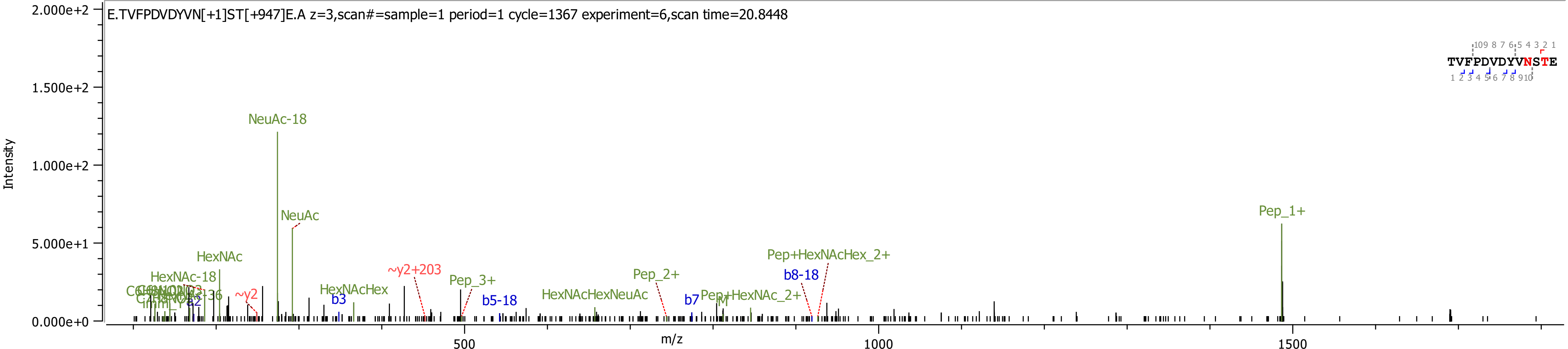
E.TVFPDVDYVN[+1]ST[+947]E.A z=3,scan#=sample=1 period=1 cycle=1367 experiment=6,scan time=20.8448
Intensity
2.000e+2
1.500e+2
1.000e+2
5.000e+1
0.000e+0
m/z
500
1000
1500
NeuAc-18
NeuAc
HexNAc
HexNAc-18
~y2
~y2+203
HexNAcHex
b3
Pep_3+
b5-18
HexNAcHexNeuAc
Pep_2+
b7
Pep+HexNAc_2+
b8-18
Pep+HexNAcHex_2+
Pep_1+
TVFPDVDYVNSTE

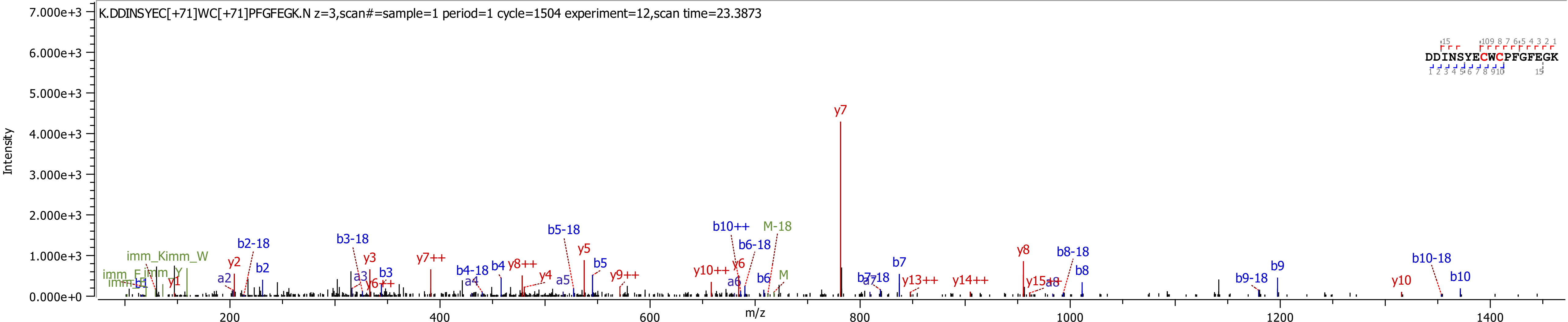
K.DDINSYEC[+71]WC[+71]PFGFEGK.N z=3,scan#=sample=1 period=1 cycle=1504 experiment=12,scan time=23.3873
Intensity
m/z
DDINSYECWCPFGFEGK
y7
y8
b7
b9
b10
y10
b10-18
b9-18
b8-18
b8
y5
b5
b5-18
b4
b4-18
a4
a5
y4
y8++
y7++
y3
b3
b3-18
a3
b2-18
b2
y2
a2
y1
imm_Kimm_W
b10++
b6-18
y6
a6
b6
M-18
M
y10++
y9++
y13++
y14++
y15++
a8
b7-18
a7
y6++
imm_F
imm_Y
imm_b1

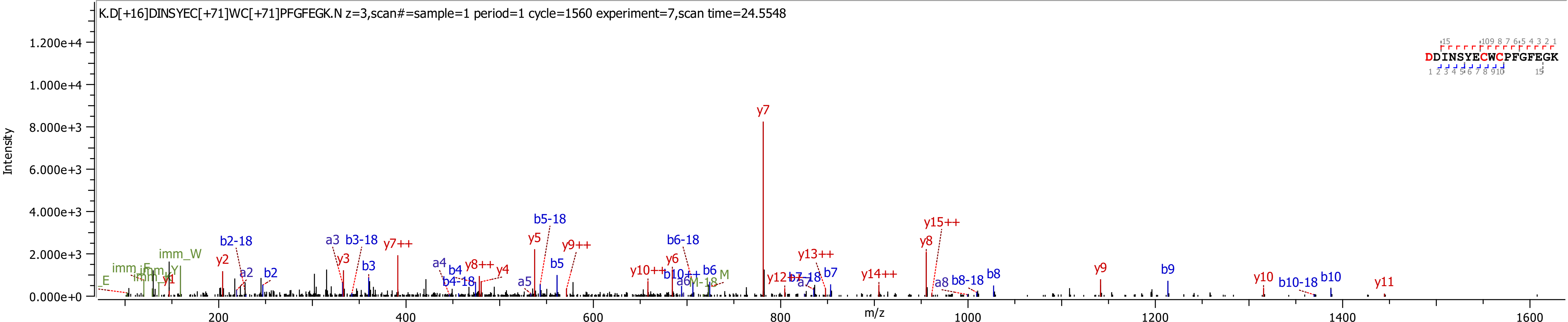
K.D[+16]DINSYEC[+71]WC[+71]PFGFEGK.N z=3,scan#=sample=1 period=1 cycle=1560 experiment=7,scan time=24.5548
DDINSYECWCPFGFEGK
Intensity
m/z
y7
b5-18
y15++
y5
y9++
y8
b2-18
a3
b3-18
y7++
b6-18
y13++
imm_W
y2
y3
a4
y8++
y4
b5
y6
b4
b3
y10++
b10++
b6
b7
y14++
y9
b9
b8
b2
a2
y1
M
y12++
b7-18
a6
M-18
a5
b4-18
a7
a8
b8-18
y10
b10
b10-18
y11
E
0.000e+0
2.000e+3
4.000e+3
6.000e+3
8.000e+3
1.000e+4
1.200e+4
200
400
600
800
1000
1200
1400
1600

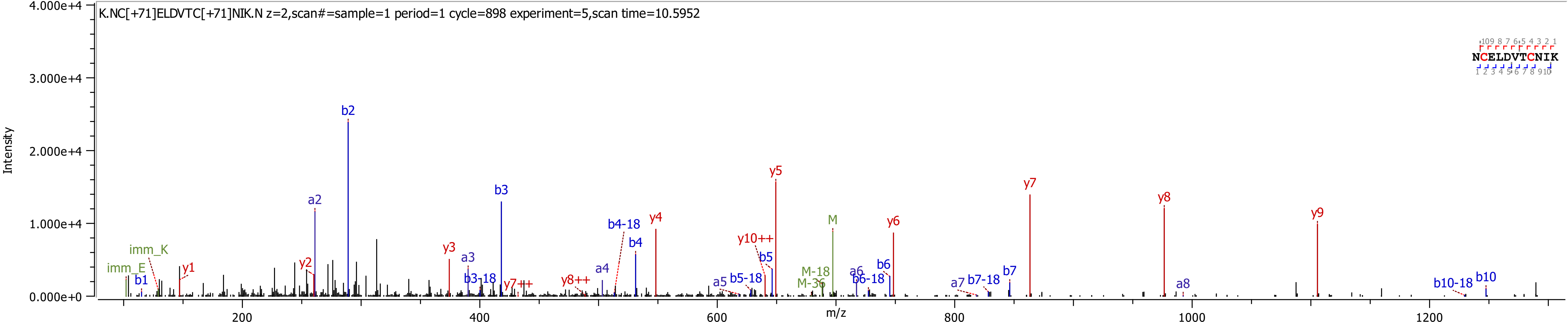

K.NC[+71]ELDVTC[+71]NIK.N z=2,scan#=sample=1 period=1 cycle=898 experiment=5,scan time=10.5952
Intensity
m/z
4.000e+4
3.000e+4
2.000e+4
1.000e+4
0.000e+0
200
400
600
800
1000
1200
NCELDVTCNIK
imm_E
imm_K
b1
y1
a2
y2
b2
y3
a3
b3-18
b3
y7++
y8++
a4
b4-18
b4
y4
a5
b5-18
y10++
b5
y5
M-18
M-36
M
a6
b6-18
b6
y6
a7
b7-18
b7
y7
y8
a8
y9
b10-18
b10

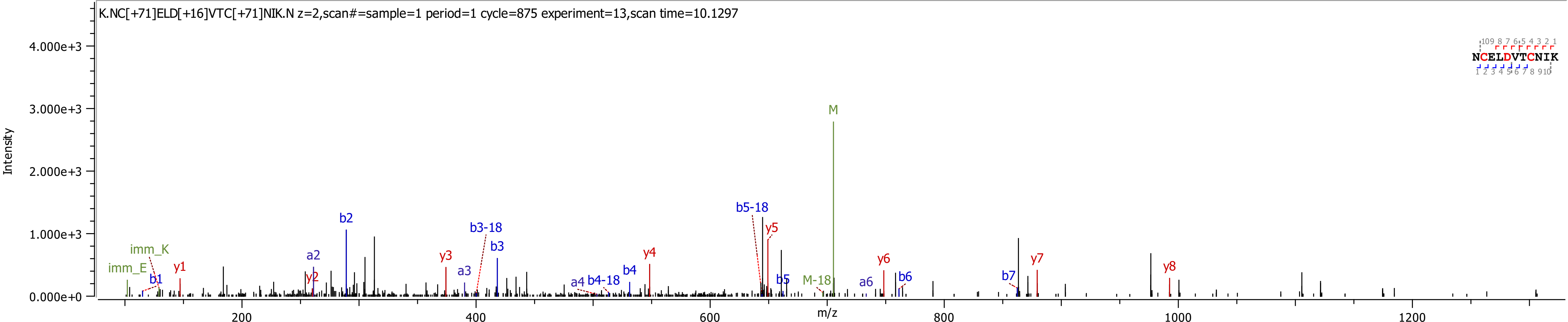
K.NC[+71]ELD[+16]VTC[+71]NIK.N z=2,scan#=sample=1 period=1 cycle=875 experiment=13,scan time=10.1297
NCELDVTCNIK
Intensity
4.000e+3
3.000e+3
2.000e+3
1.000e+3
0.000e+0
m/z
200
400
600
800
1000
1200
M
b5-18
y5
b2
b3-18
b3
imm_K
imm_E
y1
b1
a2
y2
y3
a3
a4
b4-18
b4
y4
b5
M-18
a6
y6
b6
b7
y7
y8

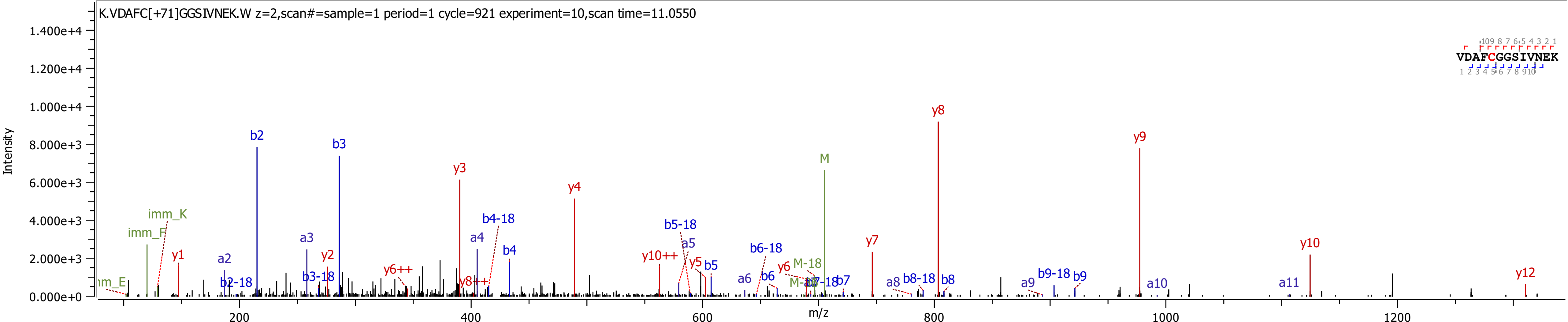
K.VDAFC[+71]GGSIVNEK.W z=2,scan#=sample=1 period=1 cycle=921 experiment=10,scan time=11.0550
Intensity
m/z
VDAFCGGSIVNEK
y1
y2
y3
y4
y5
y6
y7
y8
y9
y10
y12
y6++
y8++
y10++
b2
b3
b4
b5
b6
b7
b8
b9
b2-18
b3-18
b4-18
b5-18
b6-18
b7-18
b8-18
b9-18
a2
a3
a4
a5
a6
a8
a9
a10
a11
M
M-18
M-17
imm_K
imm_F

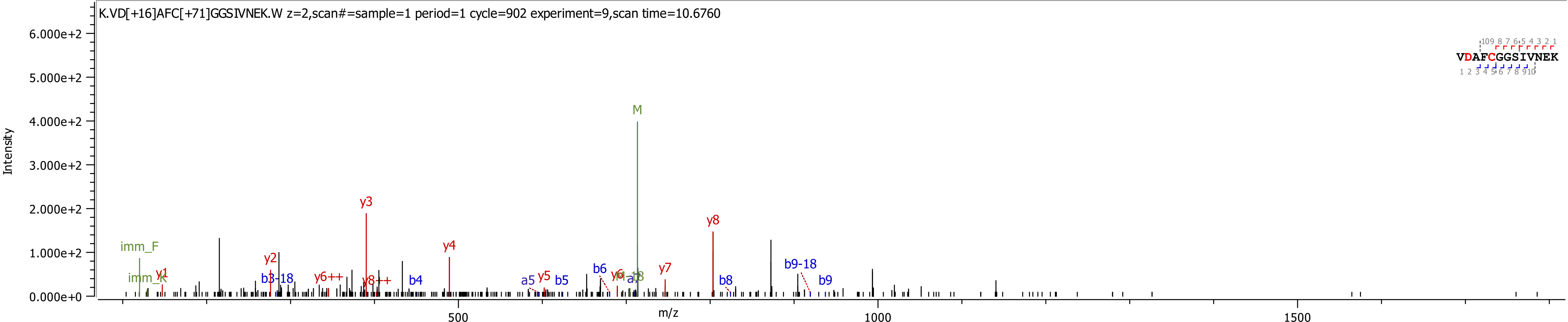

K.VD[+16]AFC[+71]GGSIVNEK.W z=2,scan#=sample=1 period=1 cycle=902 experiment=9,scan time=10.6760
VDAFCGGSIVNEK
Intensity
m/z
6.000e+2
5.000e+2
4.000e+2
3.000e+2
2.000e+2
1.000e+2
0.000e+0
500
1000
1500
M
y3
y8
y4
imm_F
imm_K
y1
y2
b3-18
y6++
y8++
b4
a5
y5
b5
b6
y7
b8
b9-18
b9

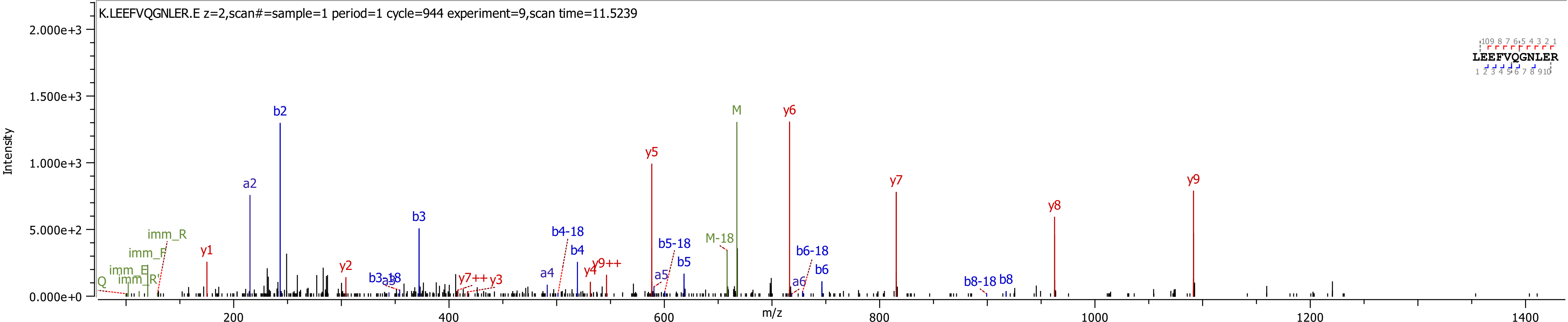
K.LEEFVQGNLER.E z=2,scan#=sample=1 period=1 cycle=944 experiment=9,scan time=11.5239
Intensity
2.000e+3
1.500e+3
1.000e+3
5.000e+2
0.000e+0
m/z
200
400
600
800
1000
1200
1400
LEEFVQGNLER
b2
a2
b3
M
y6
y5
y7
y9
y8
M-18
imm_R
imm_F
imm_E
imm_R'
Q
y1
y2
b3-18
a3
y7++
y3
a4
b4-18
b4
y4
y9++
b5-18
a5
b5
b6-18
b6
a6
b8-18
b8

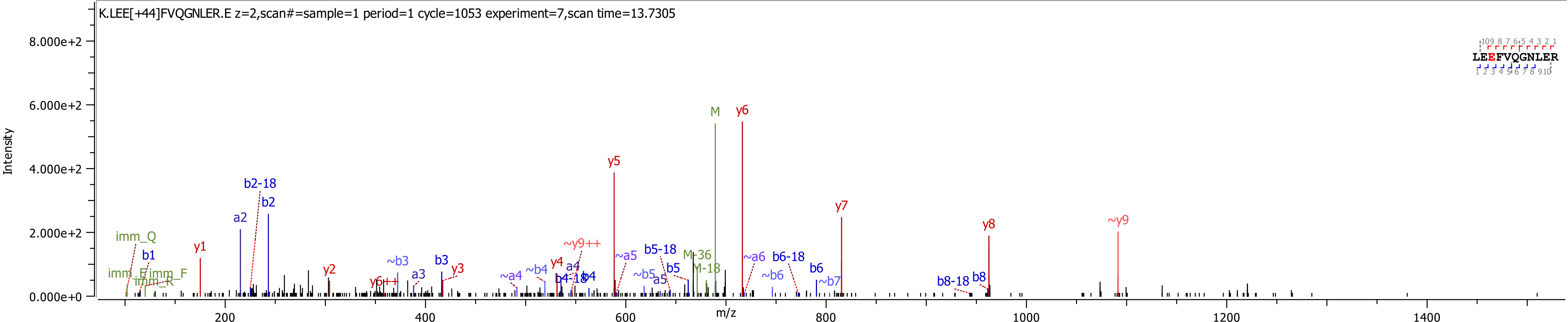
K.LEE[+44]FVQGNLER.E z=2,scan#=sample=1 period=1 cycle=1053 experiment=7,scan time=13.7305
Intensity
8.000e+2
6.000e+2
4.000e+2
2.000e+2
0.000e+0
m/z
200
400
600
800
1000
1200
1400
LEEFVQGNLER
M
y6
y5
y7
~y9
y8
b2-18
b2
a2
imm_Q
b1
y1
imm_E
imm_F
imm_R
y2
~b3
y6++
a3
b3
y3
~a4
~b4
y4
a4
b4-18
b4
~y9++
~a5
b5-18
~b5
b5
a5
M-36
M-18
~a6
b6-18
~b6
b6
~b7
b8-18
b8

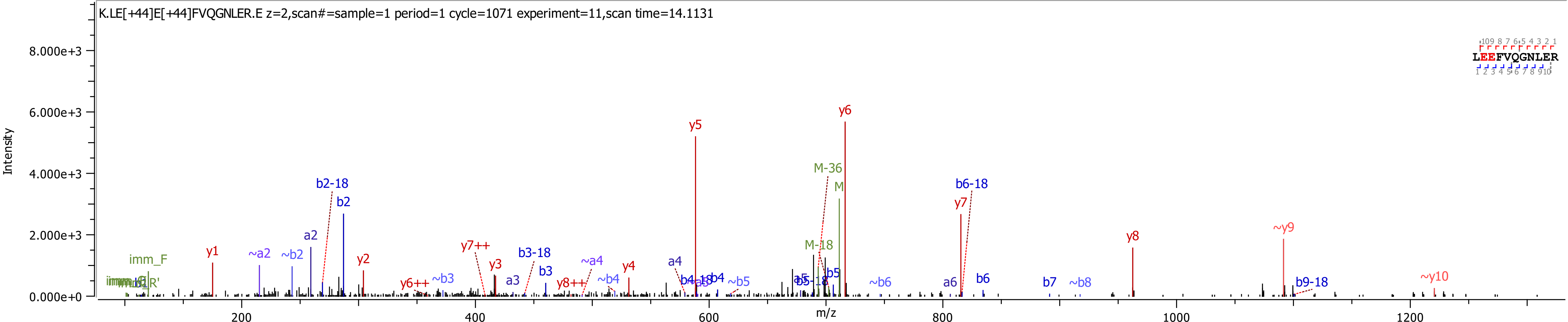

K.LE[+44]E[+44]FVQGNLER.E z=2,scan#=sample=1 period=1 cycle=1071 experiment=11,scan time=14.1131
Intensity
m/z
LEEFVQGNLER
y1
~a2
~b2
a2
b2-18
b2
y2
y6++
~b3
y7++
y3
a3
b3-18
b3
y8++
~a4
~b4
y4
a4
b4-18
a5
b4
~b5
b5-18
b5
M-36
M
M-18
y5
y6
~b6
a6
b6
y7
b6-18
b7
~b8
y8
~y9
b9-18
~y10
imm_F

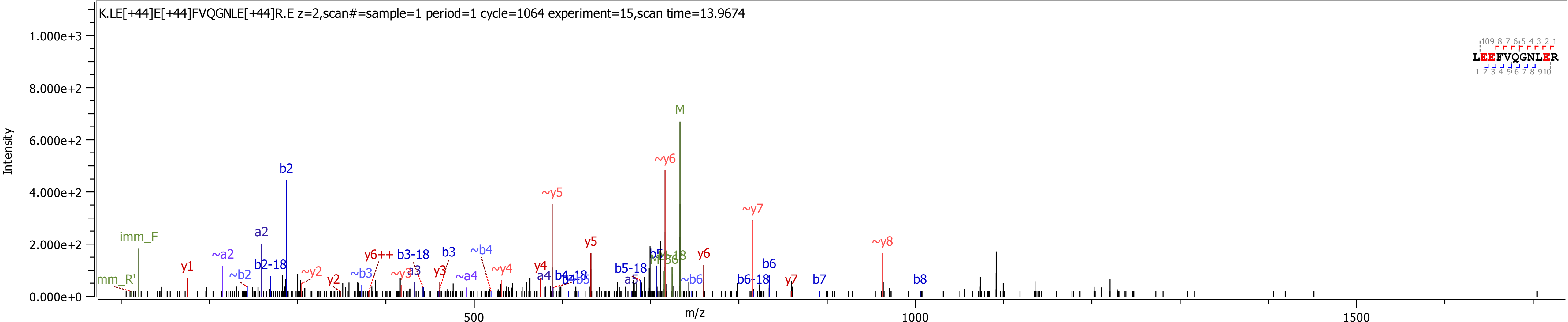
K.LE[+44]E[+44]FVQGNLE[+44]R.E z=2,scan#=sample=1 period=1 cycle=1064 experiment=15,scan time=13.9674
Intensity
m/z
1.000e+3
8.000e+2
6.000e+2
4.000e+2
2.000e+2
0.000e+0
500
1000
1500
LEEFVQGNLER
M
~y6
b2
~y5
~y7
~y8
a2
imm_F
y5
y6
b6
y1
~a2
b2-18
~b2
~y2
y2
y6++
b3-18
b3
~b4
~y4
y4
a4
b4-18
b5-18
a5
~b6
b6-18
y7
b7
b8
~b3
~y3
a3
y3
~a4
mm_R'

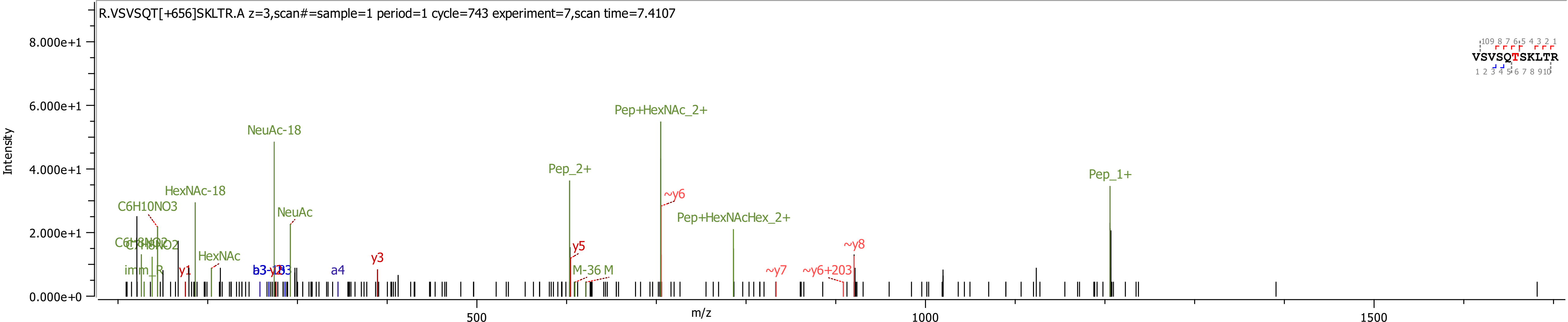
R.VSVSQT[+656]SKLTR.A z=3,scan#=sample=1 period=1 cycle=743 experiment=7,scan time=7.4107
Intensity
8.000e+1
6.000e+1
4.000e+1
2.000e+1
0.000e+0
m/z
500
1000
1500
VSVSQTSKLTR
Pep+HexNAc_2+
NeuAc-18
Pep_2+
Pep_1+
HexNAc-18
C6H10NO3
NeuAc
~y6
Pep+HexNAcHex_2+
HexNAc
imm_R
y1
a4
y3
y5
M-36 M
~y7
~y6+203
~y8

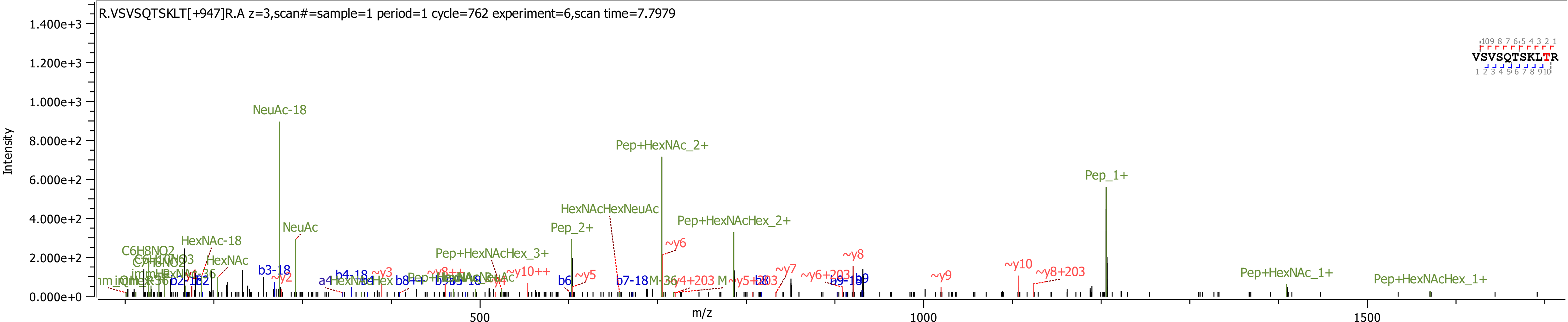
R.VSVSQTSKLT[+947]R.A z=3,scan#=sample=1 period=1 cycle=762 experiment=6,scan time=7.7979
Intensity
m/z
VSVSQTSKLTR
NeuAc-18
Pep+HexNAc_2+
Pep_1+
HexNAcHexNeuAc
Pep+HexNAcHex_2+
NeuAc
Pep_2+
HexNAc-18
C6H8NO2
HexNAc
b3-18
Pep+HexNAcHex_3+
~y6
~y8
~y10
~y8+203
~y9
~y7
~y5
~y3
~y2
b6
b7-18
b8
a9-18
b9
b4-18
a4
~y10++
Pep+HexNAc_1+
Pep+HexNAcHex_1+
500
1000
1500
0.000e+0
2.000e+2
4.000e+2
6.000e+2
8.000e+2
1.000e+3
1.200e+3
1.400e+3

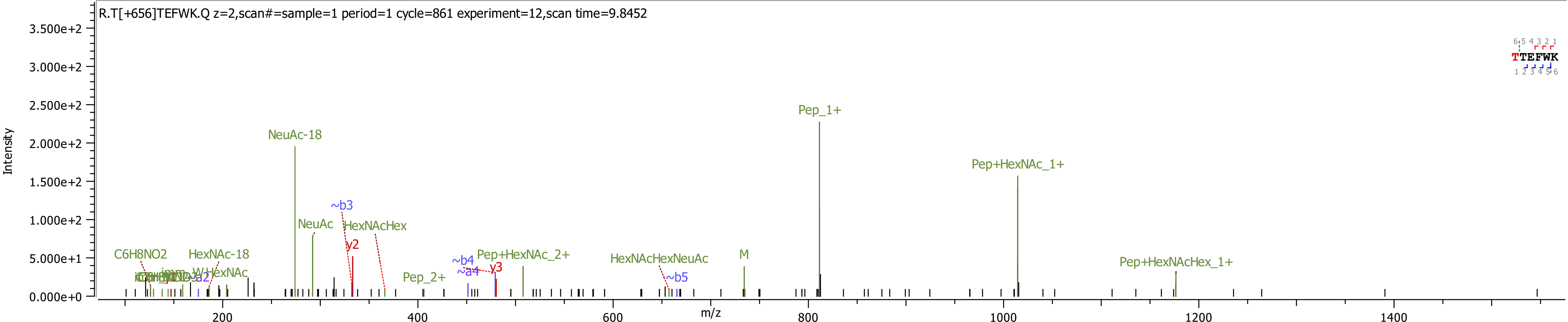
R.T[+656]TEFWK.Q z=2,scan#=sample=1 period=1 cycle=861 experiment=12,scan time=9.8452
Intensity
m/z
NeuAc-18
NeuAc
HexNAcHex
~b3
y2
C6H8NO2
HexNAc-18
Pep_2+
~b4
~a4
y3
Pep+HexNAc_2+
HexNAcHexNeuAc
~b5
M
Pep_1+
Pep+HexNAc_1+
Pep+HexNAcHex_1+
TTEFWK

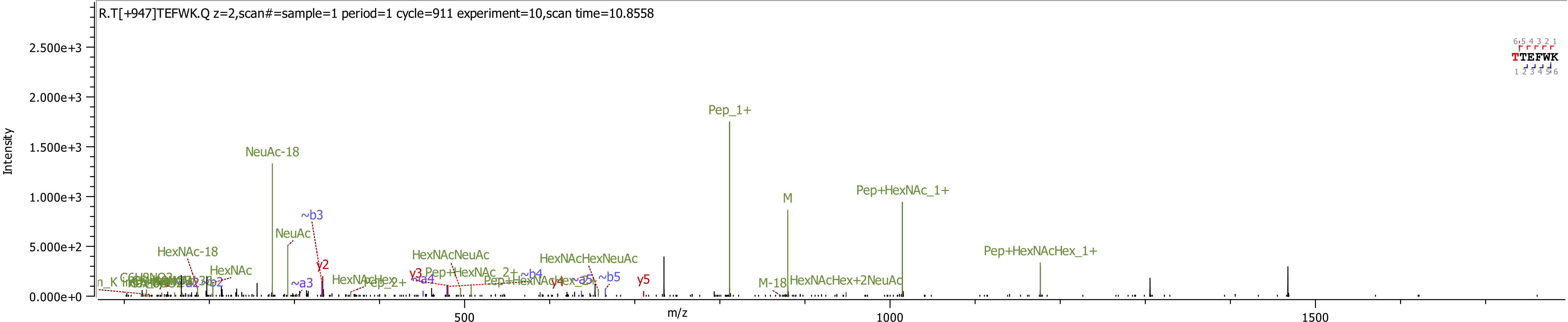

R.T[+947]TEFWK.Q z=2,scan#=sample=1 period=1 cycle=911 experiment=10,scan time=10.8558
Intensity
m/z
2.500e+3
2.000e+3
1.500e+3
1.000e+3
5.000e+2
0.000e+0
500
1000
1500
Pep_1+
NeuAc-18
M
Pep+HexNAc_1+
~b3
NeuAc
HexNAc-18
HexNAc
y2
HexNAcNeuAc
HexNAcHexNeuAc
Pep+HexNAcHex_1+
Pep+HexNAc_2+
~b4
~b5
y5
~a3
y3
~a4
y4
M-18
HexNAcHex+2NeuAc
TTEFWK

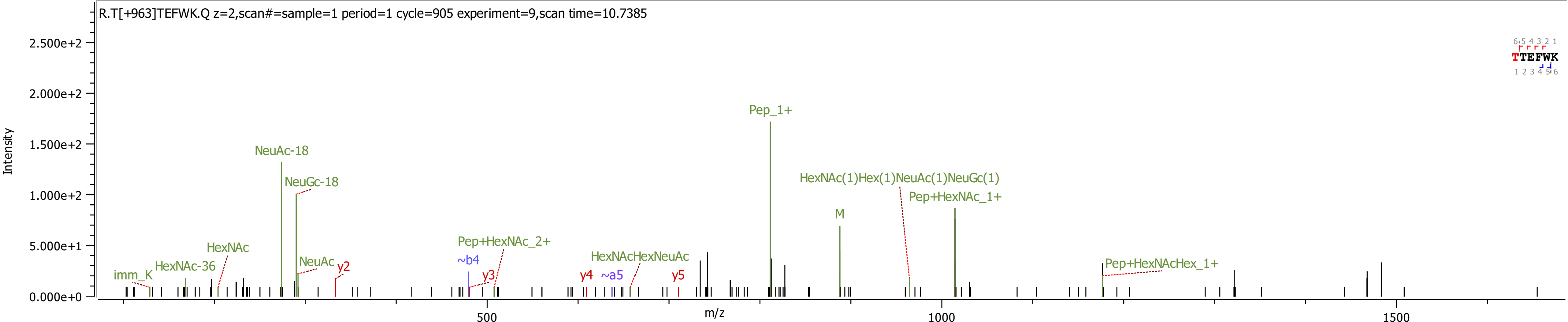
R.T[+963]TEFWK.Q z=2,scan#=sample=1 period=1 cycle=905 experiment=9,scan time=10.7385
Intensity
m/z
TTEFWK
NeuAc-18
NeuGc-18
Pep_1+
HexNAc(1)Hex(1)NeuAc(1)NeuGc(1)
Pep+HexNAc_1+
M
Pep+HexNAc_2+
HexNAcHexNeuAc
Pep+HexNAcHex_1+
imm_K
HexNAc-36
HexNAc
NeuAc
y2
~b4
y3
y4
~a5
y5

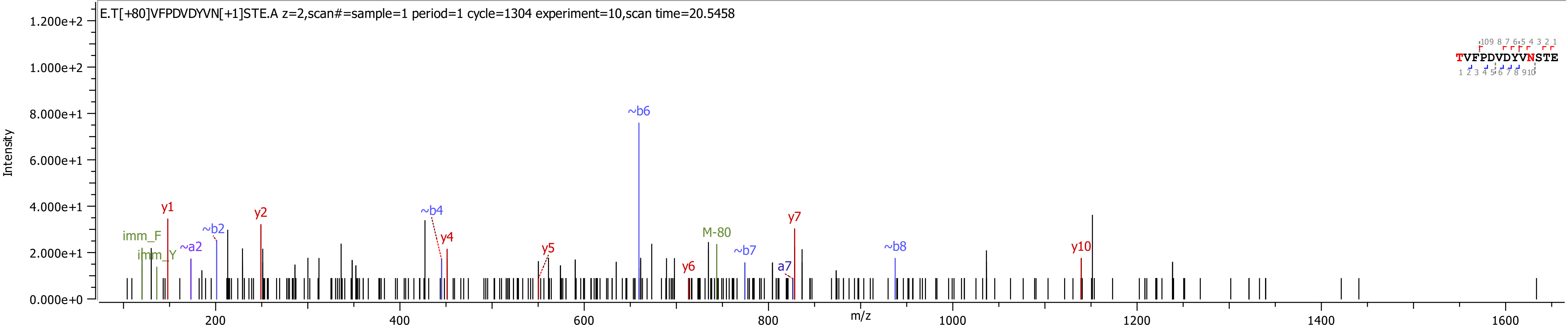

E.T[+80]VFPDVDYVN[+1]STE.A z=2,scan#=sample=1 period=1 cycle=1304 experiment=10,scan time=20.5458
Intensity
m/z
TVFPDVDYVNSTE
imm_F
imm_Y
y1
~a2
~b2
y2
~b4
y4
y5
~b6
y6
M-80
~b7
a7
y7
~b8
y10

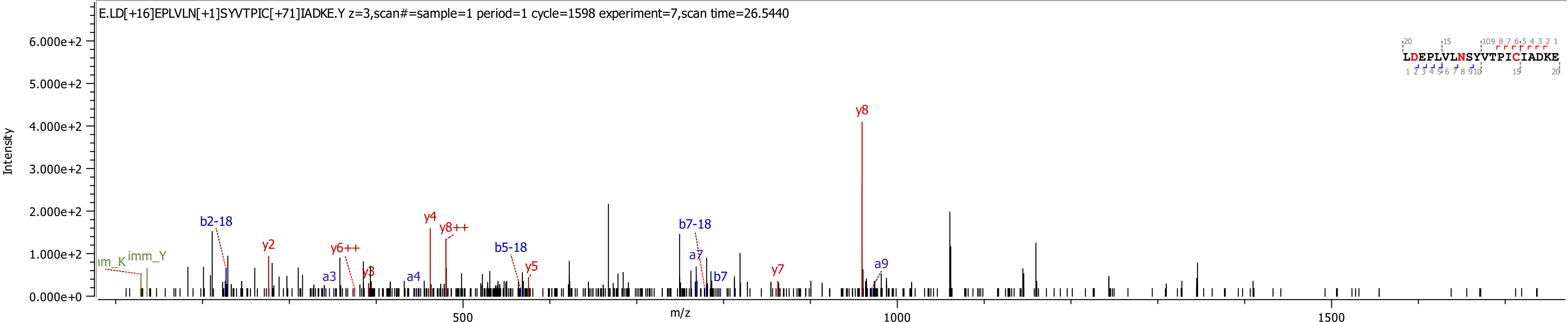

E.LD[+16]EPLVLN[+1]SYVTPIC[+71]IADKE.Y z=3,scan#=sample=1 period=1 cycle=1598 experiment=7,scan time=26.5440
Intensity
6.000e+2
5.000e+2
4.000e+2
3.000e+2
2.000e+2
1.000e+2
0.000e+0
m/z
500
1000
1500
LDEPLVLNSYVTPICIADKE
imm_Y
b2-18
y2
a3
y3
y6++
a4
y4
y8++
b5-18
y5
b7-18
a7
b7
y7
y8
a9

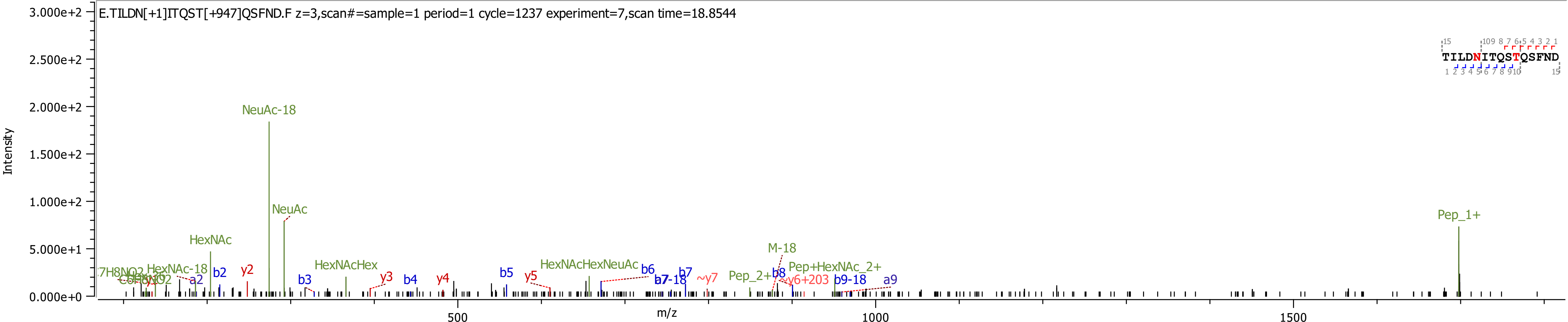
E.TILDN[+1]ITQST[+947]QSFND.F z=3,scan#=sample=1 period=1 cycle=1237 experiment=7,scan time=18.8544
Intensity
3.000e+2
2.500e+2
2.000e+2
1.500e+2
1.000e+2
5.000e+1
0.000e+0
m/z
500
1000
1500
TILDNITQSTQSFND
NeuAc-18
NeuAc
HexNAc
HexNAc-18
HexNAcHex
HexNAcHexNeuAc
Pep_1+
M-18
Pep_2+
Pep+HexNAc_2+
a2
b2
y2
b3
y3
b4
y4
b5
y5
b6
b7
b8
b9-18
a9
~y7
~y6+203

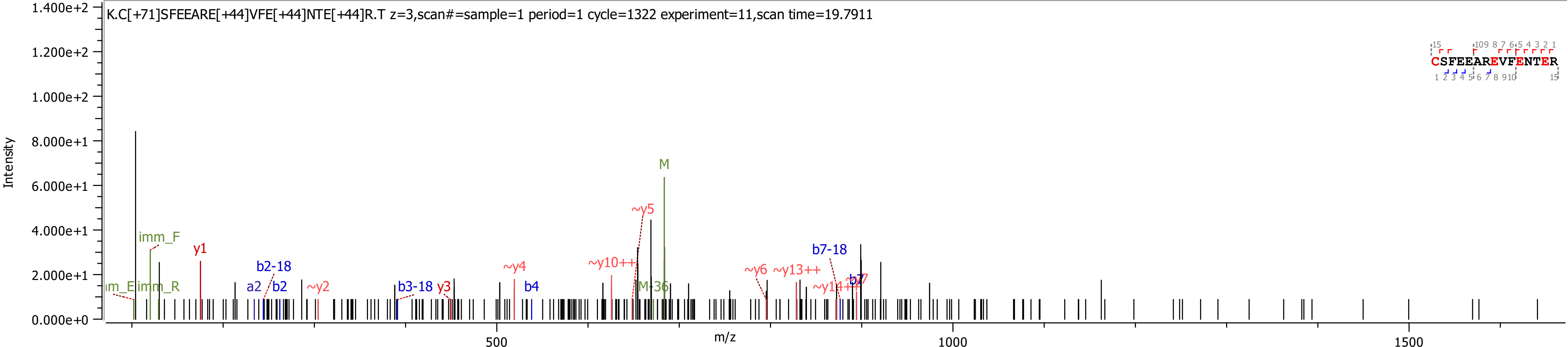
K.C[+71]SFEEARE[+44]VFE[+44]NTE[+44]R.T z=3,scan#=sample=1 period=1 cycle=1322 experiment=11,scan time=19.7911
CSFEEAREVFENTER
Intensity
1.400e+2
1.200e+2
1.000e+2
8.000e+1
6.000e+1
4.000e+1
2.000e+1
0.000e+0
m/z
500
1000
1500
M
~y5
imm_F
y1
b2-18
~y4
~y10++
~y6
~y13++
b7-18
a2
b2
~y2
b3-18
y3
b4
M-36
~y14++
b7
imm_R

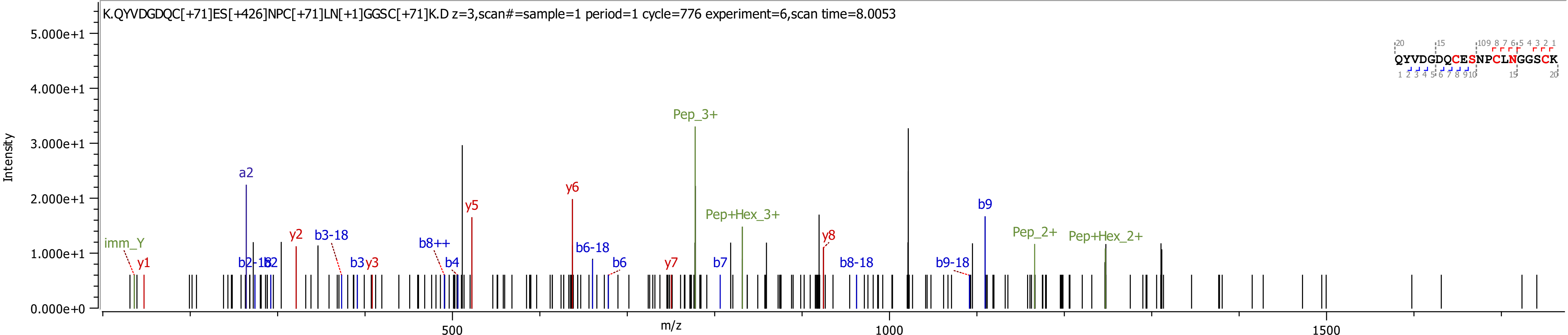

K.QYVDGDQC[+71]ES[+426]NPC[+71]LN[+1]GGSC[+71]K.D z=3,scan#=sample=1 period=1 cycle=776 experiment=6,scan time=8.0053
Intensity
m/z
0.000e+0
1.000e+1
2.000e+1
3.000e+1
4.000e+1
5.000e+1
500
1000
1500
QYVDGDQCESNPCLNGGSCK
imm_Y
y1
a2
b2-18
b2
y2
b3-18
b3
y3
b8++
b4
y5
y6
b6-18
b6
y7
Pep_3+
b7
Pep+Hex_3+
y8
b8-18
b9-18
b9
Pep_2+
Pep+Hex_2+

ProteinPilot™ Software - [Result - D:\2018\20181030_Schulz_Luci_FIXH1H2\20181106_Schulz_Luci_H2_TTP.group]

File Configure Window Help

Workflow Tasks

Identify Proteins
- LC...
- Spot-Based (MS only)...
- Spot-Based (MS and MS/MS)...

View
- Analysis Log...
- Result...

Export
- Peptide Summary...
- Distinct Peptide Summary...
- Protein Summary...
- Spectrum Summary...
- MGF Peaklist(s)...
- mzIdentML...
- Features...

Protein ID | Features | Spectra | Summary Statistics

Spectrum List

Spectrum	Acq Time	Obs MW	Obs m/z	Obs z	Prot N	Best Sequence	Modifications	Conf	Theor MW	z
3.1.1.919.3	11.303	854.3748	428.1947	2	1, 3	TTEFWK	Carboxy(E)@3	60.8	854.3810	2

Peptide ID Hypotheses - 3.1.1.919.3

Conf	Sc	Prot N	Sequence	Modifications	Theor MW	Theor m/z	Obs m/z	z	ΔMass
60.8	7	1, 3	TTEFWK	Carboxy(E)@3	854.3810	428.1978	428.1947	2	-0.0063

Precursor MS Region

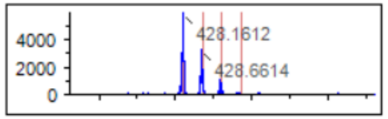

Fragmentation Evidence for Peptide

TTE[Cox]FWK

Residue	b	b+2	y	y+2
T	102.0550	51.5311	855.3883	428.1
T	203.1026	102.0550	754.3406	377.6
E[Cox]	376.1351	188.5712	653.2930	327.1
F	523.2035	262.1054	480.2605	240.6
W	709.2828	355.1450	333.1921	167.0
K	837.3777	419.1925	147.1128	74.0

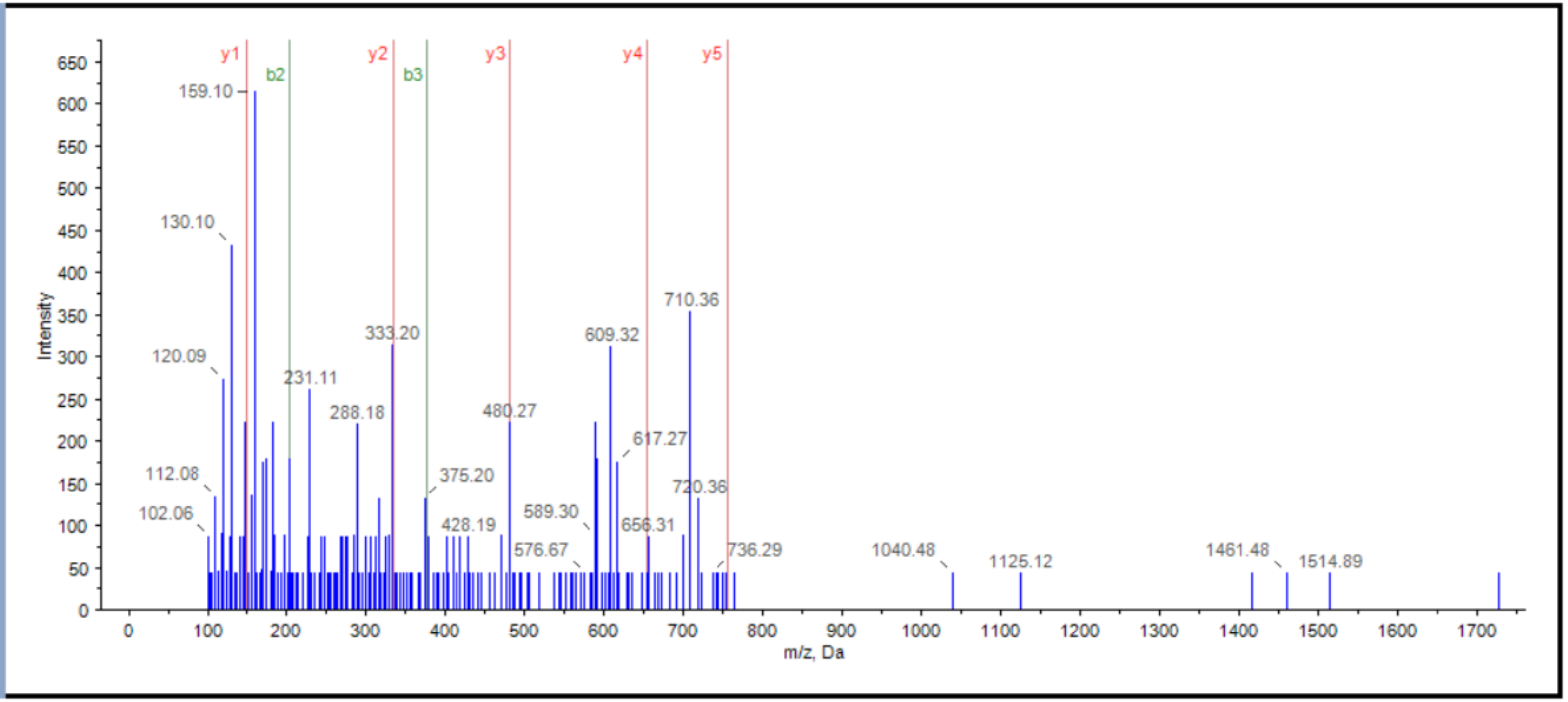

Type here to search

ENG 11:11 PM 2/04/2020
