# Supplementary material for: Coagulation factor IX analysis in bioreactor cell culture supernatant predicts quality of the purified product
Source: Commun Biol. 2021 Mar 23;4:390. doi: 10.1038/s42003-021-01903-x (PMC7988164; doi:10.1038/s42003-021-01903-x)
Supplement: Supplementary file 28 — Reporting Summary [file 42003_2021_1903_MOESM28_ESM.pdf]

nature research

Corresponding author(s): Yi Yean Lee, Benjamin L. Schulz, Christopher B. Howard

Last updated by author(s): Jul 20, 2020

Reporting Summary

Nature Research wishes to improve the reproducibility of the work that we publish. This form provides structure for consistency and transparency in reporting. For further information on Nature Research policies, see our Editorial Policies and the Editorial Policy Checklist.

Statistics

For all statistical analyses, confirm that the following items are present in the figure legend, table legend, main text, or Methods section.

n/a	Confirmed	
☐	☒	The exact sample size (*n*) for each experimental group/condition, given as a discrete number and unit of measurement
☐	☒	A statement on whether measurements were taken from distinct samples or whether the same sample was measured repeatedly
☐	☒	The statistical test(s) used AND whether they are one- or two-sided<br>*Only common tests should be described solely by name; describe more complex techniques in the Methods section.*
☒	☐	A description of all covariates tested
☒	☐	A description of any assumptions or corrections, such as tests of normality and adjustment for multiple comparisons
☐	☒	A full description of the statistical parameters including central tendency (e.g. means) or other basic estimates (e.g. regression coefficient) AND variation (e.g. standard deviation) or associated estimates of uncertainty (e.g. confidence intervals)
☐	☒	For null hypothesis testing, the test statistic (e.g. *F*, *t*, *r*) with confidence intervals, effect sizes, degrees of freedom and *P* value noted<br>*Give P values as exact values whenever suitable.*
☒	☐	For Bayesian analysis, information on the choice of priors and Markov chain Monte Carlo settings
☒	☐	For hierarchical and complex designs, identification of the appropriate level for tests and full reporting of outcomes
☒	☐	Estimates of effect sizes (e.g. Cohen's *d*, Pearson's *r*), indicating how they were calculated

Our web collection on statistics for biologists contains articles on many of the points above.

Software and code

Policy information about availability of computer code

Data collection	Total cell density, cell viability, and cell size were measured using a cell counter (Vi-cell Beckman Coulter, Fullerton, CA). Glucose, lactate, ammonium, glutamine, and other metabolites were measured with a NOVA Flex BioProfile analyzer (NOVA biomedical). pH was measured using Seven Excellence Multiparameter (METTLER TOLEDO). Oxygen (pO2) and carbon dioxide (pCO2) partial pressure were measured using a Siemens Blood Gas Analyzer RapidLab 248. Recombinant FIX was purified by ÄKTA Pure (GE Healthcare) chromatography controlled with UNICORN 7.0 software. Mass spectrometry data was obtained in a TripleTOF 5600 mass spectrometer controlled by Analyst TF1.7 (SCIEX).
Data analysis	All the software used in this study to analyze the data is either published and cited, commercially available, including Byonic (Protein Metrics), ProteinPilot and PeakView (SCIEX), and GraphPad Prism (GraphPad Software, San Diego, California USA), or can be found in Supplementary information.

For manuscripts utilizing custom algorithms or software that are central to the research but not yet described in published literature, software must be made available to editors and reviewers. We strongly encourage code deposition in a community repository (e.g. GitHub). See the Nature Research guidelines for submitting code & software for further information.

Data

Policy information about availability of data

All manuscripts must include a data availability statement. This statement should provide the following information, where applicable:

- Accession codes, unique identifiers, or web links for publicly available datasets
- A list of figures that have associated raw data
- A description of any restrictions on data availability

The mass spectrometry raw data and ProteinPilot results are available in the ProteomeXchange Consortium (http://proteomecentral.proteomexchange.org) [64] via the PRIDE partner repository [65] with the dataset identifier PXD018229. The content of each uploaded file is described in Supplementary Table S17. A summary

diagram of the proteomic workflows used in this work can be found in Supplementary Fig. S1. The results from the Byonic searches are compiled in the Supplementary material.

Field-specific reporting

Please select the one below that is the best fit for your research. If you are not sure, read the appropriate sections before making your selection.

- [x] Life sciences
- [ ] Behavioural & social sciences
- [ ] Ecological, evolutionary & environmental sciences

For a reference copy of the document with all sections, see nature.com/documents/nr-reporting-summary-flat.pdf

Life sciences study design

All studies must disclose on these points even when the disclosure is negative.

Sample size	Mass spectrometry analysis was implemented as described in Methods using triplicates to reduce retention time variation and improve data quality.
Data exclusions	No sample was excluded from the analysis. However, specific measurements were excluded if the false discovery rate was above 1%, which is standard in the field.
Replication	Our analysis of purified plasma derived FIX confirmed previous findings. Mass spectrometry characterization of rFIX was performed multiple times, with similar or different proteases, providing reproducible results. The quantitative data was obtained using independently prepared samples that were analysed together.
Randomization	Samples were randomized before loading into the mass spectrometer.
Blinding	Blinding was not necessary for this study.

Reporting for specific materials, systems and methods

We require information from authors about some types of materials, experimental systems and methods used in many studies. Here, indicate whether each material, system or method listed is relevant to your study. If you are not sure if a list item applies to your research, read the appropriate section before selecting a response.

Materials & experimental systems

n/a	Involved in the study	
[ ]	[x]	Antibodies
[ ]	[x]	Eukaryotic cell lines
[x]	[ ]	Palaeontology and archaeology
[x]	[ ]	Animals and other organisms
[x]	[ ]	Human research participants
[x]	[ ]	Clinical data
[x]	[ ]	Dual use research of concern

Methods

n/a	Involved in the study	
[x]	[ ]	ChIP-seq
[x]	[ ]	Flow cytometry
[x]	[ ]	MRI-based neuroimaging

Antibodies

Antibodies used	Mouse IgG1 anti human Factor IX primary antibody 1C2 (AbCam, 1:1000 dilution) and HRP-conjugated anti mouse IgG (BioRAD, 1:1000 dilution).
Validation	Mouse IgG1 anti human Factor IX primary antibody 1C2 (AbCam), validated by manufacturer https://www.abcam.com/factor-ixptc-antibody-1c2-ab17196.html. Our results agree with the expected western blot results for this antibody, and we used it to detect purified recombinant Factor IX.

Eukaryotic cell lines

Policy information about cell lines

Cell line source(s)	The cell line was created from a Lonza CHO-K1SV host cell using their GS expression vector by at CSL Marburg Research facilities (Germany).
Authentication	The cell line was not authenticated, but we are confident of its identity due to strict traceability procedures that are standard in CSL.
Mycoplasma contamination	Cell lines tested negative for Mycoplasma

Commonly misidentified lines<br>(See ICLAC register)	No commonly misidentified cell line was used in this study.
